# A Triple‐Catalytic, Fully Biogenic Synthesis of Cyclic Carbonates

**DOI:** 10.1002/cssc.202501973

**Published:** 2026-01-14

**Authors:** Robin Stuhr, Leon Liu, Axel Jacobi von Wangelin

**Affiliations:** ^1^ Department of Chemistry University of Hamburg Hamburg Germany

**Keywords:** biogenic materials, biomass chemicals, CO_2_ valorization, photocatalysis, photochemistry

## Abstract

Organic carbonates play a central role as functional platform molecules for the manufacture of materials and chemicals. The atom‐economical formation of cyclic carbonates from epoxides and CO_2_ under mild catalytic conditions is a prime example of the concept of green chemistry. However, the sustainability of such strategies is often limited by the unfavorable parameters of the epoxide formation from olefin oxidation. Herein, a new, highly sustainable, triple‐catalytic approach to the formation of biogenic cyclic carbonates from all‐natural building blocks is documented. Three biogenic resources (fatty acid derivatives, O_2_, CO_2_) are combined with 100% atom‐economy in the presence of easily accessible catalysts (porphyrin, VO(acac)_2_, pyridine). Key step is a photo‐oxygenation with full incorporation of O_2_ into hydrocarbons. Use of the resultant cyclic carbonates in the synthesis of environmentally benign non‐isocyanate polyurethanes is demonstrated.

## Graphical abstract

A three‐step synthesis of cyclic carbonates from biogenic resources (fatty acids, O_2_, CO_2_) is reported. The process operates via a visible light‐mediated hydrocarbon oxidation, with an overall 100% atom‐economy, in the presence of easily accessible catalysts (porphyrin, VO(acac)_2_, pyridine) but without intermediate work‐up procedures.

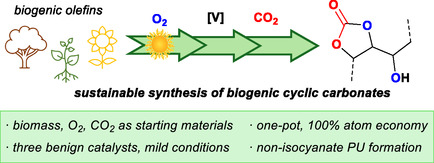

## 1 Introduction

Cyclic organic carbonates are valuable platform molecules in the context of green chemical strategies by virtue of their synthetically versatile functionalities, the utilization of carbon dioxide as chemical feedstock, their typically efficient synthesis from epoxides and CO_2_ under catalytic mild conditions, and a benign toxicological profile [1]. Ethylene and propylene carbonate, the most important examples, find widespread use as solvents [2, 3, 4], electrolyte components [5, 6], or precursors to various chemicals and materials [7]. Low‐molecular weight cyclic carbonates are generally considered nontoxic and biodegradable [8]. Organic carbonates are also common motifs in functional molecules, as diol protecting groups, and as synthetic intermediates for further derivatizations. By incorporation of reactive substituents in direct proximity to the cyclic carbonate (e.g., vinyl [9, 10], vinylene [11], hydroxyl [12]), high functional density can be obtained that enable especially diverse chemical uses. Among them, molecules with multiple cyclic carbonate moieties are especially attractive as monomers in the formation of cross‐linked materials. For example, polycarbonates easily undergo reactions with bifunctional amines to polyhydroxyurethanes (PHU), a subclass of non‐isocyanate polyurethanes (NIPU). These polymers obviate the need for the highly reactive but toxic diisocyanates in favor of the environmentally and operationally benign carbonates [13, 14, 15].

**SCHEME 1:**
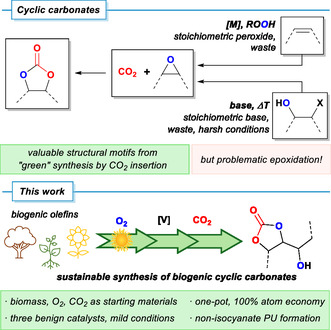
Cyclic carbonates as functional building blocks prepared by CO_2_ incorporation into epoxides. Selected epoxide‐forming reactions based on stoichiometric peroxide reagents, base‐mediated ring‐closure (*top right*), or photo‐oxygenation with O_2_ (*bottom*). The latter strategy has been implemented into a catalytic three‐step synthesis of cyclic carbonates with 100% atom economy.

The synthesis of cyclic carbonates from epoxides and CO_2_, a steadily growing area of research and manufacture, is often considered a prime example for the implementation of green chemistry concepts into industrial synthesis (Scheme 1). Indeed, the reaction features high atom economy and utilization of the waste gas CO_2_ as reagent and can be conducted in neat solution, thus adhering to the *“12 Principles of Green Chemistry”* [16, 17]. A wide range of catalysts (e.g., metal complexes or salts, acidic or basic organocatalysts, ionic liquids) has been reported [18, 19, 20]. Dual activation modes by co‐catalytic Lewis acids and nucleophiles are most common [21, 22, 23]; biogenic and recyclable catalyst have also been reported [24]. However, the epoxides employed as starting materials in carbonate syntheses are often highly reactive and toxic and require laborious and problematic synthesis routes themselves (Scheme 1). Alkenes commonly undergo epoxidation by oxygen transfer from peroxyacids (e.g., *meta*‐chloroperoxy‐benzoic acid, *m*CPBA) or via metal‐catalyzed reactions with alkyl hydroperoxides (e.g., cumyl hydroperoxide, *tert*‐butylhydro‐peroxide) [25]. Many processes require handling of sensitive and hazardous reagents, additional isolation, and purification steps and generate stoichiometric amounts of undesired byproducts, while some progress has been made with more sustainable methods such as catalytic reactions with aqueous H_2_O_2_ [26]. Alternatively, epoxides can be formed by intramolecular base‐mediated substitution of 1,2‐functionalized alcohols (diols, halohydrins) under often harsh conditions in the presence of a stoichiometric base generating stoichiometric waste [25]. These unfavorable sustainability profiles of epoxidations also pose significant limitations to applications of epoxide‐derived cyclic carbonates [27, 28]. Alternative synthesis routes toward epoxides utilizing milder reaction conditions and reagents would greatly benefit the overall impact of C–O bond formation. One approach is the photo‐oxygenation of alkenes followed by a peroxide rearrangement giving access to epoxy alcohols: Oxygen can be excited to its first singlet state (^1^O_2_) by photo‐sensitization using inexpensive organic dyes and LEDs [29]. The reactive singlet‐oxygen can then undergo a Schenck‐ene reaction with an alkene and form allyl hydroperoxides [30]. This reaction mode is well‐established and can be performed quantitatively in short reaction times in flow reactors. Our group reported the entirely solvent‐free photo‐oxygenation of several readily available alkenes [31]. The obtained allylic hydroperoxide can undergo facile self‐epoxidation reactions into epoxy alcohols in the presence of inexpensive and nontoxic Ti or V catalysts [32]. Both reactions have been successfully combined into one‐pot processes [31, 33, 34, 35]. This transformation of alkenes into epoxy alcohols using only oxygen, light, and a first‐row transition metal catalyst is exceptionally mild and presents an attractive alternative toward common epoxidation strategies. Such reactions utilize both oxygen atoms of O_2_ for the chemical functionalization of alkenes in two formal insertion steps, the CH oxygenation and a subsequent oxygen atom transfer. The resultant epoxy alcohols are well‐established substrates for CO_2_ insertion and yield hydroxy‐functionalized cyclic carbonates. The most prominent example is the transformation of glycidol into 1,2‐glycerol carbonate [36, 37, 38]; CO_2_ insertions into alkyl‐substituted epoxy alcohols are generally more challenging. The role of the neighboring free hydroxy group in the insertion mechanism and possible isomerization reactions of the resulting cyclic carbonates have been investigated (*vide infra*) [39, 40]. To the best of our knowledge, there are no reports on the use of vicinal epoxy alcohols obtained from photo‐oxygenation approaches in the CO_2_ insertion reaction. In this work, we report a fully biogenic, 100% atom‐economic three‐step conversion of unsaturated biomolecules into hydroxy‐substituted cyclic carbonates. The process utilizes inexpensive and abundant biogenic molecules (biomass‐olefins, oxygen, and carbon dioxide) as stoichiometric reagents and operates under mild conditions. The resultant cyclic carbonates were applied to the synthesis of non‐isocyanate polyurethanes, NIPU (Scheme 1).

## 2 Results and Discussion

### 2.1 Starting Materials

**SCHEME 2:**
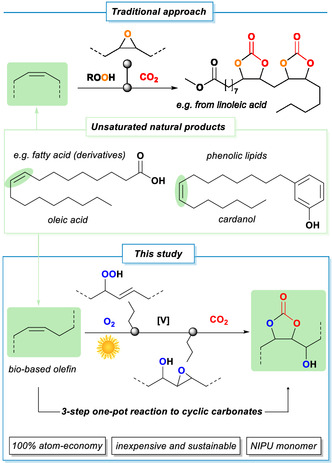
Conversion of biogenic olefins to cyclic carbonates via epoxidations with stoichiometric peroxides (*top*) or via photocatalytic O_2_ insertion (*bottom*).

We sought to combine three catalytic transformations into a fully atom‐economic preparation of cyclic carbonate building blocks for further use as platform molecules or co‐monomers. The proposed three‐step one‐pot reaction should consist of sequential photo‐oxygenation, epoxidation, and CO_2_‐insertion steps and should be based on biomass‐derived alkenes (Scheme 2). The three individual reaction steps were first optimized separately and then combined into a unified process that avoids isolations of sensitive or hazardous intermediates and waste‐generating purification steps. The choice of olefinic starting materials was strongly governed by sustainability criteria. Unsaturated molecules are ubiquitous in nature (Scheme 2); prominent examples are unsaturated fatty acids such as oleic acid. They are accessible in large quantities from vegetable oils; however, their chemical use is in competition with food production. Therefore, secondary uses of waste cooking oils are especially attractive [41, 42]. Further sustainable sources of unsaturated biomass are non‐edible oils (e.g., ricinoleic acid), phenolic lipids (e.g., cardanol waste from the cashew nut production), terpenes (limonene, pinene, etc.), and terpenoids (citronellol, geraniol, etc.). In this work, fatty acid derivatives were employed as precursors for hydroxy functionalized cyclic carbonates. Initial optimization experiments were conducted with (*Z*)‐4‐octene and methyl oleate as model substrates.

While cyclic carbonates have been previously prepared from biogenic olefins [15] (e.g., triglycerides [43, 44], castor oil [45], terpenes [46, 47]), the intermediate epoxides were formed by reactions with (often super‐stoichiometric amounts of) hazardous peroxide reagents (*vide supra*). Such conditions dramatically compromised the sustainability of the overall carbonate synthesis, so that we sought to develop an alternative strategy that would obviate any uses of hazardous reagents, accumulation of sensitive intermediates, and production of waste. Hence, we turned our attention to the use of aerial oxygen as stoichiometric oxidant and a photocatalytic activation mechanism. Such photo‐oxygenations of alkenes toward allylic hydroperoxides (Schenck‐ene reactions) operate under very mild conditions (Scheme 2). Our group previously reported both solvent‐based and solvent‐free photo‐oxidation procedures in a home‐built flow reactor to ensure effective mixing, low hazard potential, and optimal control of reaction conditions and reaction progress [48, 49]. The solvent‐free photo‐oxygenation reaction benefits from higher sustainability due to the absence of solvents, waste prevention, and higher space‐time yields. The use of only liquid substrates and products and the higher concentration of peroxide intermediates are somewhat limiting factors. The photo‐oxygenation operating in organic solvents decreases the environmental sustainability and reaction productivity but enhances the scope of substrates (to include also solid substrates and products) and the operational safety of the oxidation event. Metal‐free tetraphenylporphyrins are biodegradable photo‐sensitizers that are commonly employed in photo‐oxidation reactions. We previously demonstrated that *para*‐alkyl substituents increase the solubility in nonpolar solvents more than tenfold, from ≤0.2 to >2 mM [31]. Therefore, tetra‐(4‐isopropylphenyl)porphyrin (iPrTPP) was used as photocatalyst for the solvent‐free reaction. The stable, violet solid is easily accessible by Rothemund reaction of pyrrole and cuminaldehyde. The organic photocatalyst was directly dissolved in the liquid substrate (1.5 mM). In the solvent‐based process, the biogenic alkene (0.1 M) and the photocatalyst methylene blue (1 mM) were dissolved in the solvent (acetonitrile; dichloromethane for nonpolar substrates).

### 2.2 Step 1: Photo‐Oxygenation

**SCHEME 3:**
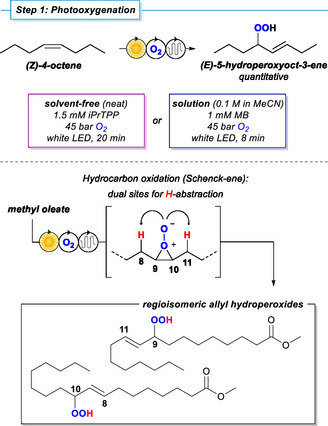
Photo‐oxygenation of olefins to allyl hydroperoxides exemplified by the two model substrates 4‐octene and methyl oleate.

The photo‐oxygenation of the olefin‐bearing hydrocarbons was performed with gaseous oxygen in a capillary tube flow reactor with the following specifications [48]: The reaction mixture (with or without solvent) was pumped through a long FEP capillary tubing (1/16″ outer diameter, 1/32″ inner diameter; irradiated tube length 12.8 m) which was coiled around a glass cylinder (outer diameter 65 mm). Flow rates of 0.20–0.50 mL/min were adjusted by an HPLC pump to enable residence times of 8–20 min. Pressurized O_2_ gas was supplied from a gas cylinder through a mass‐flow control unit, and the total system pressure was adjusted to 45 bar by an IDEX back‐pressure cartridge. The O_2_ flow rate was tuned so that a laminar slug flow resulted (0.2 mL/min for neat, 0.5 mL/min for solvent reactions; which could be easily observed by the visual inspection of the transparent O_2_ segments and colored liquid phase segments), and oxygen was not completely consumed at the downstream terminus of the tubing. Temperature control (20°C) was applied from the outside of the reactor coil by a temperature‐controlled oil bath. The reactor coil was irradiated from the inside of the glass cylinder by 24 water‐cooled white LEDs (*λ*_max_ = 630 nm) mounted on an aluminum rod. The product was collected at the outlet and analyzed by GC‐FID, GC‐MS, and NMR spectroscopy. Full conversion of the two model substrates 4‐octene and methyl oleate to the corresponding allyl hydroperoxides was achieved after 8 min for the reaction in solution and after 20 min for the solvent‐free reaction, respectively (Scheme 3, see also the Supporting Information) [49]. It is important to note that this photo‐oxygenation is a prime example of a fully atom‐economical reaction that delivers both atoms of O_2_ into the product structure by an O_2_‐insertion mechanism into a CH bond. The gaseous reagent O_2_ can easily be separated from the reaction (and reused). No byproducts were detected by ^1^H NMR and GC analysis. The isolated hydroperoxides of both model substrates were stable at room temperature for several days and could be stored at −30°C indefinitely. Generally, internal alkenes may form mixtures of regioisomers in rather equal amounts due to the presence of nearly identical allylic positions (Scheme 3) [29]. Such regioisomeric products obtained from fatty acid derivatives could not be separated nor distinguished by ^1^H NMR spectroscopy but gave two sets of signals with equal intensities in their ^13^C NMR spectra. The quantitative product formation (and absence of byproducts) allowed direct follow‐up reactions of the hydroperoxides without work‐up.

The adoption of a solvent‐based or solvent‐free synthesis strategy was governed by considerations of sustainability, productivity, and safety. Space‐time yields (STY) are important efficiency parameters of the chemical reaction and reactor setups. STY is defined as mass of the formed product *m*_p_ per reactor volume *V* and residence time *τ* (Equation (1)) [50]

(1) $$\text{Space time yields (STY)}=\frac{m_{p}}{V\cdot \tau }$$

The active oxygen content (AO) describes the reactivity/stability of an oxygen‐rich compound and is given as the number of peroxide units (*n*) per molecular weight *M* (Equation (2)).

(2) $$\text{Active oxygen content (AO)}=\frac{n\cdot 16g\cdot {\mathrm{mol}}^{-1}}{M}$$

Both parameters, STY and AO, were calculated for the photo‐oxygenations of the two model substrates, 4‐octene and methyl oleate, respectively (Table 1). The space‐time yields (STY) of the solvent‐free photo‐oxygenations were more than 10 times higher than the STY of the solvent‐based reactions (the higher STY of methyl oleate vs. 4‐octene is due to the higher molecular weight). The active oxygen content (AO) of the octene hydroperoxides is 11%; of the methyl oleate hydroperoxide 5% (*cf.* H_2_O_2_, 47%). Lower AO values, peroxides with >6 carbons, and handling of peroxides in solution (vs. solids) generally provide higher operational safety [51, 52]. Consequently, the fatty acid‐derived hydroperoxides of this study are expected to be stable and safe, even in the absence of solvents (neat). The operation of a capillary flow‐reaction avoids better control of reaction conditions and the accumulation of larger volumes of materials, including peroxides, and thus provides higher safety.

**TABLE 1:** Active oxygen content (AO) and space‐time yield (STY) of the photo‐oxygenation products from 4‐octene and methyl oleate, respectively.

Substrate	Active oxygen content (AO) of peroxide [%]	**Space‐time yield (STY) [g L** ^**−1**^ ** h** ^**−1**^ **]**	
		Solvent‐free	0.1 M in MeCN
4‐Octene	11.1	1550	60
Methyl oleate	4.9	1600	140

### 2.3 Step 2: Oxygen Transfer/Epoxidation

**SCHEME 4:**
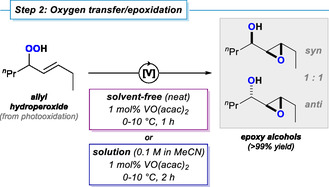
Vanadium‐catalyzed self‐epoxidation of allyl hydroperoxides to epoxy alcohols by an oxygen transfer exemplified by the 4‐octene pathway.

The synthesis of epoxides from olefin‐containing hydrocarbons is considered the critical step of organic carbonate syntheses by virtue of the use of (excess amounts of) hazardous peroxide reagents, the generation of waste materials, and the often adoption of very specific reaction conditions. With our synthesis strategy, the reactive peroxides were generated in solution from aerial dioxygen which obviates the need for the availability, storage, handling, and addition of excess peroxide reagents, their quenching, and potential side product separation. A subsequent oxygen transfer within the oxidized hydrocarbon from step 1 gives an epoxy alcohol motif in the presence of titanium or vanadium catalysts (Scheme 4, see the Supporting Information). This formal self‐epoxidation of the alkene (which serves a dual role as oxidant and substrate) by the vicinal hydroperoxide function is greatly under‐utilized in the literature, but constitutes a transformation of utmost synthetic value as it delivers 1,2,3‐trioxy‐functionalized derivatives [31, 53]. Titanium(IV) and vanadium(V) compounds are abundant, inexpensive, and typically hazard‐free, so that their use as catalysts aligns well with modern sustainability criteria. Previous studies documented the benefits of using the readily available and benign vanadyl(V) catalyst VO(acac)_2_ [49, 50]. Solvent‐free conditions and reactions in solution (0.1 M in acetonitrile, or DCM for nonpolar substrates, see Supporting Information) were investigated with 1 mol% catalyst at 0°C. Full conversion of octene hydroperoxide was observed after 0.5 h (neat) and 2 h (in MeCN), respectively. The isolated epoxy alcohols were obtained as mixtures of diastereomers (*syn*/*anti* = 1:1; by ^1^H NMR). Under the same conditions, the reaction of methyl oleate hydroperoxide, containing two regioisomers (*vide supra*), gave full conversion to a mixture of regio/stereoisomeric epoxy alcohols. Side products (e.g., reduction to allyl alcohol, dehydration to enone) were not observed. The full utilization of the abundant and inexpensive molecule O_2_, the catalytic conditions, and the formation of hydroxy‐functionalized epoxides differentiate this method from conventional syntheses of epoxides.

The solvent‐free self‐epoxidation of the octene hydroperoxide from step 1 occurred highly exothermic and required cooling the reaction to prevent boiling of the reaction mixture. Such exothermicity was not observed for the longer chain methyl oleate. Therefore, the solvent‐free approach is only recommended for small‐scale reactions and/or the use of substrates with low active oxygen content that show moderate exothermicity. The solvent‐free reaction did not proceed with the catalyst VOSO_4_·H_2_O, most likely due its very low solubility. The reaction mechanism of the vanadium‐catalyzed oxygen transfer of hydroperoxides to give epoxy alcohols is not fully elucidated. It is assumed that the reaction operates by a similar mechanism as the epoxidation of allyl alcohols with *tert*‐butyl hydroperoxide, involving an intermediate peroxo‐vanadium species that transfers the metal‐bound oxygen atom to a proximal alkene [54, 55].

### 2.4 Step 3: CO_2_ Insertion

The final transformation of our three‐step carbonate synthesis is the CO_2_ insertion into the epoxy alcohols from step 2 to obtain the desired cyclic carbonates. Most commonly, combinations of Lewis acids and nucleophiles are used which follow a mechanism of dual substrate activation [56]. The epoxide is coordinated to the Lewis acid (e.g., a metal ion) and thus activated for ring‐opening substitution by the nucleophilic co‐catalyst (e.g., bromide). CO_2_ insertion into the metal alkoxide bond delivers a carbonate intermediate that undergoes intramolecular ring‐closing substitution upon elimination of the nucleophilic co‐catalyst [57]. An alternative mechanism can be adopted for epoxides with neighboring hydroxy functions involving a base‐assisted nucleophilic attack on carbon dioxide. The resultant carbonate effects epoxide ring‐opening by an intramolecular substitution. Importantly, both mechanisms form different regioisomers of the cyclic carbonates (see for example the products derived from 4‐octene in Table 2) [36, 37]. Intramolecular substitution may result in isomerization between the regioisomers [39, 40].

**TABLE 2:** Selected optimization experiments.

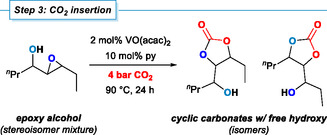		
Entry	**Deviation from optimized conditions** a	**Yield [%]** b
1	5 mol% py, 40 bar CO_2_	93
2	5 mol% Et_3_N (*i/o* py), 40 bar CO_2_	54
3	—	91
4	1 mol% VO(acac)_2_, 5 mol% py	35
5	No VO(acac)_2_, 5 mol% py	19
6	No py	<2
7	No py, 2.5 mol% (^*n*^Octyl)_4_NBr	63

a Optimized conditions: 2 mol% VO(acac)_2_, 10 mol% pyridine, 4 bar CO_2_, 90°C, 24 h.

b ^1^H NMR yield. py = pyridine. *i/o* = instead of.

Optimizations of the CO_2_ insertion reaction were performed in a pressure reactor with the epoxy alcohol derived from (*Z*)‐4‐octene (Table 2). VO(acac)_2_ was employed as Lewis acid catalyst as it may enable a reaction sequence of the vanadium‐catalyzed oxygen transfer (step 2) and the carbonate synthesis (step 3) using the same catalyst species (*vide infra*). Reactions at higher CO_2_ pressure (40 bar) with 2 mol% VO(acac)_2_ and 5 mol% pyridine showed high conversion and high yields of the cyclic carbonates by ^1^H NMR spectroscopy (93%, entry 1). The use of triethylamine, co‐catalytic bromide, or lower catalyst loadings, respectively, afforded significantly lower yields (35%–65%). High yields were also obtained when operating at lower pressure (4 bar CO_2_) with 10 mol% pyridine (91%, entry 3). Very low conversions were observed in the absence of the vanadium catalyst (19%, entry 5) or base (traces, entry 6), respectively. The optimized conditions involved reaction of the neat epoxy alcohol with 2 mol% VO(acac)_2_ and 10 mol% pyridine at 4 bar CO_2_ and 90°C for 24 h.

### 2.5 Three‐Step Reaction Sequence

**SCHEME 5:**
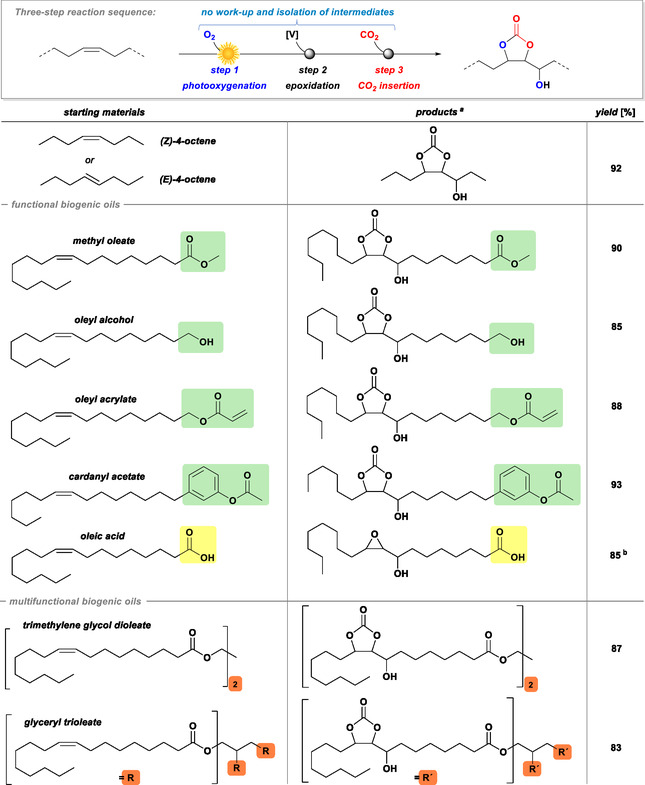
*Top*: Three‐step sequence of photo‐oxygenation (step 1), self‐epoxidation (step 2), and CO_2_ insertion (step 3) without isolation of reaction intermediates. Middle: Substrate scope of octenes, oleic acid, and cardanol derivatives with functional end groups. *Bottom*: Di‐ and tri‐oleates enable convenient access to multifunctional networking agents. (a) mixture of regio‐ and stereoisomers and (b) yield of epoxy alcohol; CO_2_ insertion gave complex product mixture.

We have established optimized conditions for the three‐step synthesis of cyclic carbonates via photo‐oxygenation, self‐epoxidation, and CO_2_ insertion starting from the two model substrates 4‐octene and methyl oleate, respectively. To further increase the efficiency of this sustainable process, we performed the three sequential reactions without isolation and purification of reaction intermediates (Scheme 5), which greatly minimizes the consumption of material and energy (no solvents, additives, adsorbents, labor for work‐up procedures). Solvent‐free reactions were set up in the flow reactor with the sensitizer iPrTPP dissolved in the substrate (1 mM), which reacted for 20 min (residence time) under illumination with white LEDs at 45 bar O_2_ pressure (step 1). The products were collected downstream, cooled to 0°C, and 1 mol% VO(acac)_2_ was added to enable catalytic self‐epoxidation (step 2). After 1 h, 10 mol% pyridine was added and the reaction vial was transferred into a pressure reactor and stirred under 4 bar CO_2_ pressure for 16 h at 90°C. Reactions in solution were conducted in a similar manner: The substrate (0.1 M) and methylene blue (1 mM) were dissolved in dichloromethane or acetonitrile and then reacted in the flow reactor (8 min residence time, white LEDs, 45 bar O_2_). The obtained reaction mixture was cooled to 0°C and 1 mol% VO(acac)_2_ was added. After 2 h, the solvent was evaporated, 10 mol% pyridine added to the neat products, and the CO_2_ insertion reaction was performed as above. All three reaction steps—photo‐oxygenation, self‐epoxidation, and CO_2_ insertion—afford mixtures of regioisomers and stereoisomers. Therefore, this synthesis strategy has great impact on materials applications based on isomer mixtures (e.g., polymers, resins, networks, and surfactants).

### 2.6 Substrate Scope

We extended the scope of this operationally facile three‐step methodology to other inexpensive and readily available biogenic oils (Scheme 5, see also the Supporting Information). Most natural lipids contain double bonds in *Z*‐configuration; *E*‐unsaturated fatty acids (“*trans*‐fats”) can be formed by isomerization during heating or as a side reaction during technical hydrogenation. Importantly, the photo‐oxygenation of (*E*)‐ and (*Z*)‐olefins are stereoconvergent processes that produce the same product mixture. Accordingly, (*E*)‐ and (*Z*)‐4‐octene afforded the same hydroperoxides (*vide supra*). We then employed various biogenic olefins in the three‐step sequence to assess a broader scope and the compatibility with functional groups. Esters, free alcohols, and aromatic moieties (from cardanol) were tolerated in every reaction step and gave very high isolated yields (85%–93%, Scheme 5). Oleyl acrylate was successfully reacted (88%); a radical stabilizer (hydroquinone monomethylether, 15 ppm) was added after photo‐oxidation to inhibit unwanted polymerization. The intermediate hydroperoxides were not worked up but directly subjected to the vanadium‐catalyzed epoxidation. The resultant cyclic carbonate derivative may find applications as bifunctional crosslinking agent in hybrid polymer formulations via polyurethane (at the carbonate unit) or polyacrylate (at the acrylate unit) forming reactions. Free oleic acid was successfully transformed in steps 1 and 2 to the epoxy alcohol. The CO_2_ insertion (step 3, with 1.1 equiv. py) gave a complex product mixture (by NMR) without detectable carbonate and thus not further characterized.

### 2.7 Polymer Synthesis

**SCHEME 6:**
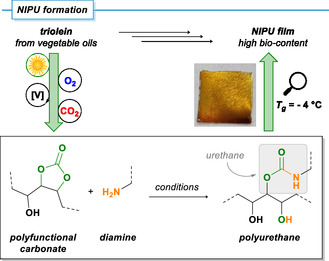
Proof‐of‐concept of a vegetable oil‐to‐polyurethane process: Sequential three‐step reactions of triolein to a polyfunctional hydroxy carbonate and onward reaction with hexamethylene diamine to a biogenic NIPU film.

Molecules with multiple cyclic carbonate functionalities (*f*) were formed from trimethylene glycol dioleate (*f* = 2) and glyceryl trioleate (*f* = 3). These are promising monomers for the formation of various polymers, for example non‐isocyanate polyurethanes (NIPUs). Combinations of difunctional carbonates (*f* = 2) and diamines would give linear polymers; polyfunctional monomers (*f* > 2) lead to the formation of cross‐linked polyurethane networks. The synthesis of NIPUs from carbonated biogenic oils is of utmost importance to technical implementations of green chemistry [58, 59, 60]. Here, we wish to briefly demonstrate as a proof‐of‐concept that the reaction of the prepared cyclic carbonates with amines readily give thermodynamically robust urethanes. The extra hydroxyl function may provide an additional anchor for diversification, cross‐linking, or stabilization [61, 62, 63]. The cyclic carbonate product of glyceryl trioleate (TO‐CC) was chosen as model compound for NIPU formation (Scheme 6, see also the Supporting Information). The commercial starting material triolein contained saturated stearic acid impurities; therefore, the average number of double bonds and resultant cyclic carbonate moieties was below the theoretical value (*f* = 2.7, determined by ^1^H NMR). A mixture of TO‐CC, hexamethylene diamine (functional group ratio of carbonate/ amine = 1.1/1), and DABCO as catalyst in dichloromethane solution at room temperature was poured into an aluminum dish, the solvent was evaporated, and the polymer cured at 90°C for 48 h. A solvent‐free neat reaction under otherwise identical conditions afforded similar results (see Supporting Information). A clear, slightly orange polymer film was obtained. FTIR analysis proved the formation of urethane bonds (1714, 1530 cm^−1^). Residual carbonate functions were observed due to their excess stoichiometry (1793 cm^−1^) but mostly due to incomplete reaction of the sterically confined internal carbonate groups (which was also proven by reaction of 8‐CC with 1‐hexylamine, ∼40% conversion, by ^1^H NMR, see Supporting Information). Gel contents (54% for DCM‐based thin‐film reaction under ambient condition; 84% for neat reaction under ambient, 94% for neat reaction under inert conditions) indicate successful formation of polymer networks. The presence of air/moisture appeared to diminish gel contents (see Supporting Information). Thermogravimetric analysis (TGA) showed stability of the films to slightly above 200°C (with <5% weight loss). These data align well with reported decompositions of NIPUs at 200°C–250°C [15]. The glass‐transition temperature was between *T*_g_ = −4°C and +2°C (by dynamic scanning calorimetry, DSC). Similar *T*_g_ values were reported for fatty acid‐based NIPUs [63]. Further properties of the polyurethanes (dynamic mechanical analysis, rheometry) will be studied in great detail within a dedicated project on biomass‐derived materials.

## 3 Conclusion

A three‐step process for the transformation of alkenes and unsaturated fatty acid derivatives into hydroxy‐functionalized cyclic carbonates was developed that displays near‐perfect atom economy by the full incorporation of aerial dioxygen and carbon dioxide. The sequence of photo‐oxygenation, self‐epoxidation, and CO_2_ insertion operates under mild reaction conditions with O_2_ and CO_2_ as the only stoichiometric reagents in the presence of low amounts of inexpensive and nonhazardous catalysts (methylene blue or tetraphenylporphyrin, VO(acac)_2_, pyridine). The cyclic carbonates were obtained in high yields via a reaction sequence that was performed without intermediate workup or isolation procedures. Polyfunctional carbonates bearing alcohol, ester, arene, and acrylate functions were prepared. As a proof‐of‐concept, the application of such biogenic carbonates to the synthesis of polyurethane materials was demonstrated. The resultant non‐isocyanate polyurethane (NIPU) exhibited stability to above 200°C (<5% weight loss) and a glass‐transition at *T*_g_ = −4°C to 2°C. The overall chemical valorization of biomass waste oils constitutes an especially successful example of the adoption of green chemistry principles, especially with regard to substrate availability, atom utilization, waste generation, energy consumption, and safety [64]. Further applications of such sustainable biomass‐to‐polymer transformations by the utilization of the abundant reagents O_2_, CO_2_, and biogenic oils/fats, including waste oil mixtures, are currently ongoing.

## 4 Experimental

### 4.1 General

Please see the Supporting Information for full details! The experiments involve pressurized O_2_ and should be conducted in appropriate equipment with sufficiently high pressure ratings. Hydroperoxides need to be handled with great preparative care due to potential explosivity, especially in contact with metal ion impurities. Commercial chemicals (>95% purity) were used as obtained without further purification; triolein (65%), oleyl alcohol (80%), and oleic acid (90%) were of lower purity. Technical NC‐700 CNSL resin was obtained from Cardolite as a free sample. TLC was performed using commercial silica gel‐coated aluminum plates (DC Kieselgel 60 F254, Merck); visualization by UV light. TLC plate staining was realized with a solution of phosphomolybdic acid in ethanol. Product yields were determined from isolated materials after flash chromatography on silica gel (Acros Organics, mesh 35–70). O_2_ gas (99.995%) was applied with a Brooks Instr. mass‐flow control; the system pressure was adjusted by an IDEX back‐pressure cartridge. Commercial FEP tubings (1/16″ OD, 1/32″ ID) were purchased from Bohlender. The internal volume of the reactor containing the substrate during the reaction was used for calculation of the space‐time‐yields. Details on reactor setup, experimental procedures, analytical methods, and spectroscopic data are given in the Supporting Information.

### 4.2 General Procedure for Neat/Solvent‐Free Reactions

iPrTPP (1.5 mM) was dissolved in the liquid olefinic starting material; ultrasonification assured a homogenous, particle‐free solution. The mixture was injected to the micro flow reactor developed by Schachtner et al. [48], at 20°C and irradiated for 20 min with 24 white (*λ*_max_ = 630 nm) LEDs in an approximately 13 m long 1/16 inch (0.79 mm) inner diameter FEP tubing, at 45 bar O_2_. The O_2_ flow rate was adjusted so that a laminar slug flow resulted and the gas was not completely consumed at the back‐pressure cartridge at the end of the tubing. The crude hydroperoxide solution was not worked up nor isolated but directly used in the next step (an aliquot was taken for the determination of conversions by TLC and ^1^H NMR spectroscopy). Then, the crude mixture was cooled to 0°C, stirred vigorously, and VO(acac)_2_ (1 mol%) was added slowly. After 0.5 h, full conversion was confirmed by TLC. For NMR analysis, a small aliquot was taken, dissolved in ethyl acetate, and filtered over silica to remove residual catalyst. The crude epoxy alcohol mixture was used as starting material in the following reaction. A vial, equipped with a stir bar, was charged with the crude epoxy alcohol (0.6 mmol), VO(acac)_2_ (1 mol%, 6·10^−6^ mol, 1.6 mg), and pyridine (10 mol%, 6·10^−5^ mol, 4.8 µL) and sealed with a crimp top, in which a cutoff canula was inserted. A stainless‐steel Parr Instr. autoclave, containing up to seven vials, was then filled with CO_2_ (4 bar) and heated to 90°C while stirring. After 24 h, the autoclave was cooled to ∼8°C in an ice bath and depressurized slowly while stirring. The cyclic carbonate product was obtained by flash chromatography on silica with pentane/ethyl acetate as eluent.

### 4.3 General Procedure for Reactions in Solvent

The olefinic starting material (0.1 M) and methylene blue (1 mM, 1 mol%) were dissolved in acetonitrile or dichloromethane; ultrasonification assured a homogenous and particle‐free solution. The solution was injected to the microflow reactor developed by Schachtner et al. [48], at 20°C, and irradiated for 8 min with 24 white (*λ*_max_ = 630 nm) LEDs in an approximately 13 m long 1/16 inch (0.79 mm) inner diameter FEP tubing, at 45 bar O_2_ pressure. The O_2_ flow rate was adjusted so that a laminar slug flow resulted and the gas was not completely consumed when the solution left the back‐pressure cartridge at the end of the tubing. The crude hydroperoxide solution was not worked up nor isolated but directly used in the next step (an aliquot was taken for the determination of conversions by TLC and ^1^H NMR spectroscopy). Then, the hydroperoxide solution was cooled to 0°C, VO(acac)_2_ (1 mol%) was added slowly under stirring, and the mixture was allowed to warm to room temperature. After 2 h, full conversion was confirmed by TLC. For NMR analysis, a small aliquot was taken and filtered over silica to remove residual catalyst. After solvent removal under reduced pressure, the crude epoxy alcohol product was used as starting material in the following reaction. A vial, equipped with a stir bar, was charged with the corresponding epoxy alcohol (0.6 mmol), VO(acac)_2_ (1 mol%, 6·10^−6^ mol, 1.6 mg), and pyridine (10 mol%, 6·10^−5^ mol, 4.8 µL) and sealed with a crimp top, in which a cutoff canula was inserted. A stainless‐steel Parr Instr. autoclave, containing up to seven vials, was then filled with CO_2_ (4 bar) and heated to 90°C while stirring. After 24 h, the autoclave was cooled to 8°C in an ice bath and depressurized slowly while stirring. The cyclic carbonate was obtained by flash chromatography on silica with pentane/ethyl acetate as eluent.

## Supporting Information

Additional supporting information can be found online in the Supporting Information section. Full details of the synthesis and characterization of substrates, intermediates, and products – including original spectra – are given in the Supporting Information (SI). The authors have cited additional key references in the SI [65–69]. **Supporting Fig. S1:** Emission spectrum of the used white LED. **Supporting Fig. S2:** Flow reactor setup (top left), filled reactor coil (top middle), T‐mixer (top right), schematic overview of the flow setup (bottom). **Supporting Fig. S3:** TGA curves of NIPU films 1 (green) and 3 (orange). **Supporting Fig. S4:** Exemplary DSC measurement of NIPU film 1. **Supporting Fig. S5:** IR‐spectrum of monomer 8‐CC*.* **Supporting Fig. S6:** IR‐spectrum of NIPU film 1. **Supporting Fig. S7:** IR‐spectrum of NIPU film 2. **Supporting Fig. S8:** IR‐spectrum of NIPU film 3. **Supporting Fig. S9:** IR‐spectra of 8‐CC before (black) and after the reaction with hexylamine (orange). **Supporting Fig. S10:** Overlay of the ^1^H NMR spectra (in CDCl_3_) of 8‐CC (red) and its reaction product with 1‐hexylamine (black). The reaction afforded a complex reaction mixture with incomplete conversion and the formation of various regioisomers and stereoisomers. From the integration of the ^1^H resonances at 2.32 ppm (CH_2_—C(=O)—O—) and 0.88 pm (Me‐CH_2_—), a conversion of approx. 40% was determined. **Supporting Table S1:** Optimization experiments for CO_2_ insertion. **Supporting Table S2:** Gel content and *T*_g_ for the differently prepared NIPUs. **Supporting Table S2:** Observed IR‐bands in the monomer and NIPU films and the assigned bond vibrations.

## Conflicts of Interest

The authors declare no conflicts of interest.

## Supporting information

Supplementary Material
