# Supplementary material for: A Triple‐Catalytic, Fully Biogenic Synthesis of Cyclic Carbonates
Source: ChemSusChem. 2026 Jan 14;19(1):e202501973. doi: 10.1002/cssc.202501973 (PMC12802824; doi:10.1002/cssc.202501973)
Supplement: Supplementary file 1 — Supplementary Material [file CSSC-19-e202501973-s001.pdf]

A Triple-Catalytic, Fully Biogenic Synthesis of Cyclic Carbonates

Robin Stuhr, Leon Liu, and Axel Jacobi von Wangelin*

Department of Chemistry, University of Hamburg

Martin-Luther-King-Pl. 6, 20146 Hamburg, Germany

** E-mail: axel.jacobi@uni-hamburg.de*

Table of Contents

1. Materials and Methods 1
2. Synthesis of Starting Materials and Catalysts 3
3. Optimization Experiments 6
4. General Procedures 8
5. Analytical Data of Synthesized Compounds 10
6. NIPU Formation and Analysis 21
7. References 28
8. Experimental Spectra 29

1. Materials and Methods

Commercial chemicals (> 95 % purity) were used as obtained without further purification; triolein (65 %), oleyl alcohol (80 %) and oleic acid (90 %) were of lower purity. Technical NC-700 CNSL resin was obtained from *Cardolite* as a free sample. TLC was performed using commercial silica gel coated aluminum plates (DC Kieselgel 60 F_{254}, *Merck*); visualization was done using UV light. Staining was realized with a solution of phosphomolybdic acid in ethanol. Product yields were determined from isolated materials after flash chromatography on silica gel (*Acros Organics*, mesh 35–70). NMR spectral data was collected on a *Bruker* FourierHD 300 (300 or 400 MHz for ^{1}H; 75 or 151 MHz for ^{13}C) at 25 °C using $CDCl_3$ as solvent. The quantification of ^{1}H cores was obtained by integration of resonance signals. Abbreviations used in ^{1}H NMR spectra: s – singlet, d – doublet, t – triplet, m – multiplet. Low-resolution mass spectroscopy was conducted on an *Agilent* 6890N GC-system coupled to a 5975 MSD unit and H_2 as carrier gas. UV/Vis spectra were measured on the Cary5000 spectrometer by *Agilent*. High resolution mass spectrometry (HRMS) was carried out by the Central Analytics at the department of chemistry, University of Hamburg. IR spectra were obtained on ALPHA Platinum ATR-IR and VERTEX 70 FTIR spectrometer by *Bruker*. TGA was performed on *Netzsch* TG 209 F1 Libra with following temperature program: 25–150 °C with a heating rate of 20 K/min, 150–200 °C with 5 K/min, 200 °C 5 min hold at 200 °C. Dynamic scanning calorimetry was conducted on *Mettler Toledo* DSC 1, the sample was heated to 150 °C, cooled to -70 °C and again heated to 150 °C with a heating/cooling rate of 10 K/min. Data evaluation was performed with STARe software V16.10. The emission spectrum of the used white LEDs and pictures of the flow reactor setup are shown in Figure S1 and S2.

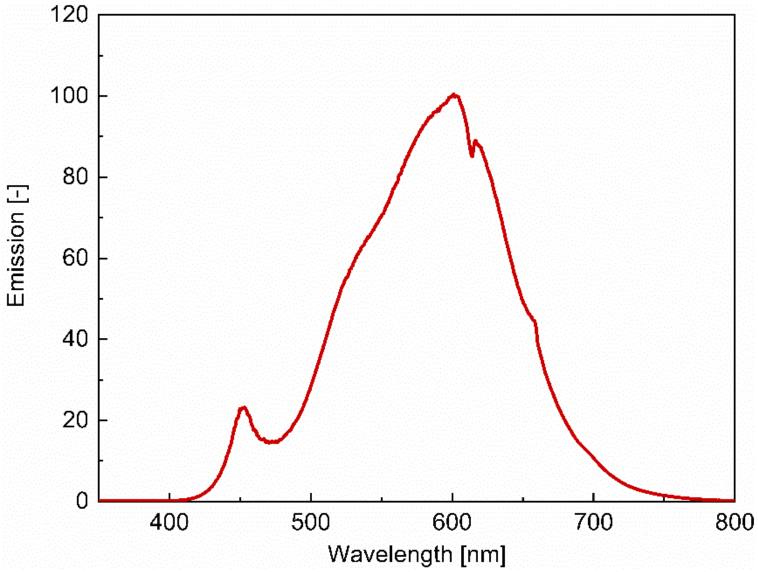

Figure S1. Emission spectrum of the used white LED.

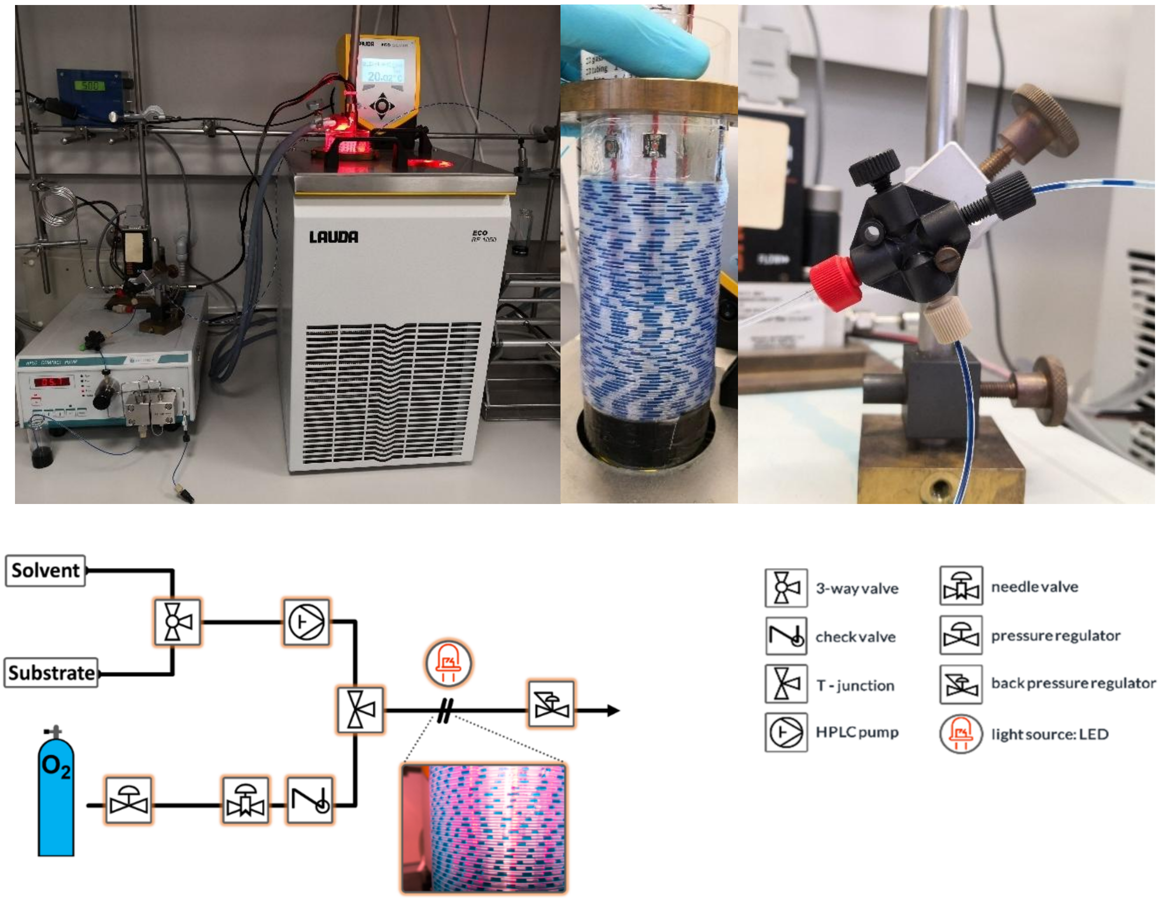

Figure S2. Flow reactor setup (top left), filled reactor coil (top middle), T-mixer (top right), schematic overview of the flow setup (bottom).

2. Synthesis of Starting Materials and Catalysts

Methyl oleate (2)

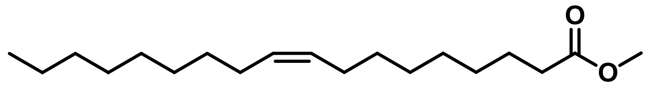

A solution of oleic acid (4.5 g, 16 mmol), trimethyl orthoformate (4.4 mL, 40 mmol) and one drop of sulfuric acid (96 %) in methanol (10 mL) was stirred for 16 hours at room temperature. The solvent was removed under reduced pressure and the red residue was diluted with diethyl ether (60 mL) and washed with sodium bicarbonate solution (2x 20 mL) and water (2x 20 mL). A dark brown oil formed while washing and was discarded. The organic phase was dried over sodium sulfate. After solvent removal under reduced pressure, the product was obtained as slightly yellow liquid (4.6 g, 15 mmol, 94 %).

^{1}H NMR (400 MHz, $CDCl_3$, ppm): δ = 5.40–5.26 (m, 2H), 3.65 (s, 3H), 2.29 (t, J = 7.5 Hz, 2H), 2.09–1.91 (m, 4H), 1.68–1.54 (m, 2H), 1.39–1.20 (m, 20H), 0.87 (t, J = 6.7 Hz, 3H).

The spectral data are consistent with literature values.[65]

Cardanyl acetate (5)

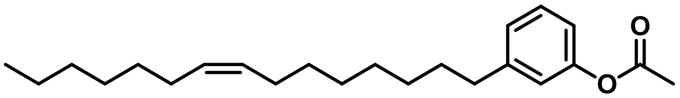

Cardanol monoene (0.51 g, 1.7 mmol) and triethylamine (0.34 mL, 2.4 mmol) were dissolved in dichloromethane (15 mL). Acetyl chloride (0.20 g, 0.18 mL, 2.2 mmol) dissolved in dichloromethane (1.5 mL) was slowly added to the reaction at 0 °C. After 16 hours stirring at room temperature, the solvent was removed from the reaction mixture under reduced pressure. The residue was redissolved in dichloromethane (10 mL) and the organic phase was washed with water (10 mL), hydrochloric acid (0.1 M, 3x 10 mL), half-saturated sodium bicarbonate solution (10 mL) and brine (10 mL). After drying over sodium sulfate, the solvent was removed under reduced pressure. Cardanyl acetate was obtained as orange oil (0.55 g, 1.6 mmol, 94 %).

^{1}H NMR (300 MHz, $CDCl_3$, ppm): δ = 7.30–7.24 (m, 2H), 7.05–7.03 (m, 2H), 6.92–6.87 (m, 2H), 5.38–5.32 (m, 2H), 2.63–2.58 (m, 2H), 2.29 (s, 3H), 2.04–1.98 (m, 4H), 1.63–1.58 (m, 2H), 1.35–1.24 (m, 16H), 0.90–0.86 (m, 3H). **^{13}C NMR** (151 MHz, $CDCl_3$, ppm): δ = 169.7, 150.8, 144.8, 130.1, 130.0, 129.2, 126.1, 121.5, 118.8, 35.9, 31.9, 31.2, 29.9, 29.5, 29.4, 29.3, 29.1, 27.4, 27.3, 22.8, 21.3, 14.3.

Oleyl acrylate (6)

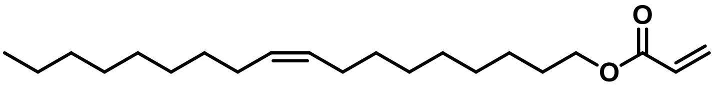

Oleyl alcohol (5.02 g, 18.6 mmol) and triethylamine (3.4 mL, 24 mmol) were dissolved in dichloromethane (185 mL). Acryloyl chloride (2.0 g, 1.8 mL, 22 mmol) dissolved in dichloromethane (15 mL) was slowly added to the reaction at 0 °C. After 16 hours stirring at

room temperature, the solvent was removed from the reaction mixture under reduced pressure. The residue was redissolved in dichloromethane (10 mL) and the organic phase was washed with water (70 mL), hydrochloric acid (0.1 M, 3x 80 mL), half-saturated sodium bicarbonate solution (80 mL) and brine (80 mL). After drying over sodium sulfate, the solvent was removed under reduced pressure. Oleyl acrylate was obtained as yellow oil (5.56 g, 17.2 mmol, 92 %).

^{1}H NMR (500 MHz, $CDCl_3$, ppm): δ = 6.39 (dd, *J* = 17.4, 1.6 Hz, 1H), 6.11 (dd, *J* = 17.4, 10.5 Hz, 1H), 5.80 (dd, *J* = 10.4, 1.5 Hz, 1H), 5.41–5.29 (m, 2H), 4.14 (t, *J* = 6.8 Hz, 2H), 2.15–1.92 (m, 4H), 1.66 (m, 2H), 1.41–1.21 (m, 22H), 0.87 (t, *J* = 6.8 Hz, 3H).

The spectral data are consistent with literature values.[66]

Trimethylene glycol dioleate (7)

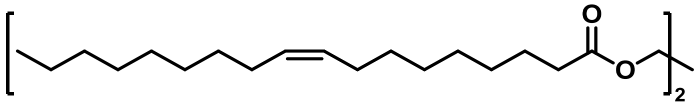

Thionyl chloride (8.4 mL, 115 mmol) was added dropwise to a cooled solution of oleic acid (25 g, 88 mmol) in chloroform (150 mL) and stirred for four hours. After solvent removal under reduced pressure, oleyl chloride was obtained by distillation (200 °C, 10 mbar). The purity was checked by ^{1}H NMR spectroscopy. The oleyl chloride (8.5 g, 28 mmol) was dissolved in chloroform (60 mL) and slowly added to a solution of 1,3-propandiol (1.0 mL, 14 mmol) and pyridine (3.0 mL, 38 mmol) in $CHCl_3$ (20 mL) and stirred for 16 hours at room temperature. The reaction mixture was washed with water (2x 100 mL), diluted hydrochloric acid (5%, 100 mL) and saturated $NaHCO_3$ solution (100 mL), and the solvent was removed under reduced pressure. After flash chromatography on silica with EA/pentane as eluent (gradient 1/500–1/10) trimethylene glycol dioleate was obtained as colorless oil (5.3 g, 8.7 mmol, 62 %)

^{1}H NMR (500 MHz, $CDCl_3$, ppm): δ = 5.36–5.33 (m, 4H), 4.12 (q, *J* = 7.1 Hz, 1H), 2.28 (t, *J* = 7.5 Hz, 1H), 2.06–1.98 (m, 8H), 1.64–1.59 (m, 4H), 1.30–1.25 (m, 42H), 0.97–0.74 (m, 6H). **^{13}C NMR** (75 MHz, $CDCl_3$, ppm): δ = 173.9, 130.0, 129.8, 60.2, 34.4, 31.9, 29.8, 29.7, 29.5, 29.3, 29.2, 29.1, 29.1, 27.2, 27.2, 25.0, 22.7, 14.3, 14.1.

5,10,15,20-Tetrakis(4-*iso*propylphenyl)porphyrin (iPrTPP)

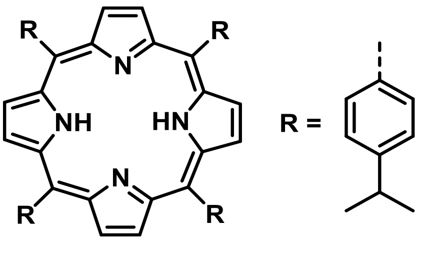

4-Isopropyl-benzaldehyde (1.48 g, 10 mmol) and pyrrole (0.7 mL, 10 mmol) were added to a hot mixture (120 °C) of acetic acid (75 mL) and nitrobenzene (50 mL). The temperature was maintained for 4 h, then the solution was cooled to room temperature to give crystals of the

product porphyrin, which were filtered off and dried. Then, the dye dissolved in dichloromethane, methanol was added, only dichloromethane was carefully removed under reduced pressure, and the product was precipitated at -30 °C. The porphyrin was filtered and washed with methanol until the washing solution was colorless. Finally, the product was dried under reduced pressure to give dark pink crystals (492 mg, 0.63 mmol, 25 % yield).

^{1}H NMR (500 MHz, $CDCl_3$, ppm): δ = 8.87 (s, 8H), 8.15–8.13 (m, 8 H), 7.61–7.59 (m, 8H), 3.26 (sept, J = 7.0 Hz, 4H), 1.55 (d, J = 6.9 Hz, 24H), –2.75 (s, 2H). **^{13}C NMR** (126 MHz, $CDCl_3$, ppm): δ = 148.3, 139.8, 134.9, 124.9, 120.3, 34.3, 24.4. **UV/Vis** ($CHCl_3$): λ_{max} = 421, 518, 554, 593, 650 nm.

The spectral data are consistent with literature values.[67]

3. Optimization Experiments

For the optimization of the CO_2 insertion reaction, a vial, equppied with a stirrer bar, was filled with epoxy alcohol (*EpAlc*, 0.6 mmol), metal catalyst, additive and mesitylene (10 mol%) and sealed with a crimp top, in which a cut-off canula was inserted. The autoclave, containing up to seven vials, was then filled with CO_2 and heated to 90 °C while stirring. After different time spans, the autoclave was cooled to 8°C using an ice bath and depressurized slowly while stirring. The ratios of cyclic carbonate to epoxy alcohol (*CC*:*EpAlc*) is reported in table S1.

Table S1. Optimization experiments for CO_2 insertion.

Metal-catalyst	Additive	CO_2 [bar]	*t* [h]	*T* [°C]	*CC*:*EpAlc*
–	10 mol% DIPEA	40	48	90	79:21
1 mol% $VO(acac)_2$	2.5 mol% TOAB	40	48	90	94:6
1 mol% $VO(acac)_2$	10 mol% DIPEA	40	48	90	95:5
2 mol% $VO(acac)_2$	10 mol% DIPEA	40	48	90	95:5
2 mol% [VO(^{iPr}TPP)]	10 mol% DIPEA	40	48	90	80:20
2 mol% $VO(acac)_2$	10 mol% DIPEA	40	24	90	78:22
2 mol% $VO(acac)_2$	5 mol% DIPEA	40	24	90	70:30
2 mol% $VO(acac)_2$	5 mol% Et_3N	40	24	90	54:46
2 mol% $VO(acac)_2$	5 mol% pyridine	40	24	90	93:7
2 mol% $VO(acac)_2$	5 mol% DBU	40	24	90	97:3
2 mol% $VO(acac)_2$	5 mol% DABCO	40	24	90	94:6
–	5 mol% pyridine	4	24	90	19:81
1 mol% $VO(acac)_2$	5 mol% pyridine	4	24	90	35:65
2 mol% $VO(acac)_2$	10 mol% pyridine	4	24	90	91:9
2 mol% $VO(acac)_2$	2.5 mol% TOAB	4	24	90	63:37
2 mol% $VO(acac)_2$	2 mol% Na_3(citrate) · 2 H_2O	4	24	90	<5:95
5 mol% $Ti(OiPr)_4$	5 mol% pyridine	4	24	90	<5:95
5 mol% $Ti(OiPr)_4$	–	4	24	90	<5:95

4. General Procedures

*General procedure for solvent-free photooxygenation (**GP1**)*

iPrTPP (1.5 mM) was dissolved in the starting material, ultrasonification was used to assure a homogenous and particle-free solution. The solution was then injected to the micro flow reactor developed by Schachtner et al.[48] at 20 °C and irradiated for 20 min with 24 white (λ_{max} = 630 nm) LEDs in an approximately 13 m long 1/16 inch (0.79 mm) inner diameter FEP tubing, together with oxygen at a pressure of roughly 45 bar. The O_2 flow rate was adjusted to 0.2 mL/min so that a laminar slug flow resulted, and the gas was not completely consumed when the solution left the back-pressure cartridge at the end of the tubing. For NMR-analysis, a small portion was taken, dissolved in ethyl acetate and filtered over silica to remove residual catalyst. The crude product was used as starting material in the following reaction. If not stated otherwise, quantitative yields of hydroperoxide were obtained.

*General procedure for oxygenation in solution (**GP2**)*

The starting material was dissolved in a solution of methylene blue (1 mM, in MeCN or dichloromethane) to give a 0.1 M solution of the substrate (1 mol% of MB sensitizer with respect to the substrate), ultrasonification was used to assure a homogenous and particle-free solution. The solution was then injected to the micro flow reactor developed by Schachtner et al.[48] at 20 °C and irradiated for 8 min with 24 white (λ_{max} = 630 nm) LEDs in an approximately 13 m long 1/16 inch (0.79 mm) inner diameter FEP tubing, together with oxygen at a pressure of roughly 45 bar. The O_2 flow rate was adjusted to 0.5 mL/min so that a laminar slug flow resulted and O_2 was not completely consumed when the solution left the back-pressure cartridge at the end of the tubing. For NMR-analysis, a small portion was taken and filtered over silica to remove residual catalyst. The crude product was used as starting material in the following reaction either in solution or neat after solvent removal. If not stated otherwise, quantitative yields of hydroperoxide were obtained.

*General procedure for solvent-free self-epoxidation (**GP3**)*

The substrate was cooled to 0 °C in an ice bath and stirred vigorously, then $VO(acac)_2$ (1 mol%) was added slowly. After 0.5 hours, full conversion was confirmed by TLC. For NMR-analysis a small portion was taken, dissolved in ethyl acetate and filtered over silica to remove residual catalyst. The crude product was used as starting material in the following reaction. If not stated otherwise, quantitative yields of epoxy alcohols were obtained.

*General procedure for self-epoxidation in solution (**GP4**)*

The substrate was dissolved in dichloromethane or acetonitrile (0.1 M) and cooled to 0 °C in an ice bath. $VO(acac)_2$ (1 mol%) was added slowly under stirring. After two hours (the bath had warmed to ~10 °C), and full conversion of the reaction was confirmed by TLC. For NMR-analysis a small portion was taken and filtered over silica to remove residual catalyst. After solvent removal under reduced pressure, the crude product was used as starting material in the following reaction. If not stated otherwise, quantitative yields of epoxy alcohols were obtained.

*General procedure for CO_2 insertion (**GP 5**)*

A vial, equipped with a stirrer bar, was filled with the corresponding epoxy alcohol (0.6 mmol), $VO(acac)_2$ (1 mol%, $6.0 \cdot 10^{-6}$ mol, 1.6 mg) and pyridine (10 mol%, $6.0 \cdot 10^{-5}$ mol, 4.8 μL), and sealed with a crimp top, in which a cut-off canula was inserted. An autoclave, containing up to seven vials, was then filled with CO_2 (4 bar) and heated to 90 °C while stirring, which resulted in a reaction pressure of approx. 4.5 bar. After 24 h, the autoclave was gradually cooled to 8°C using an ice bath and depressurized slowly while stirring.

5. Analytical Data of Synthesized Compounds

Reaction sequence for (Z)-4-octene and (E)-4-octene (1)

***trans*-5-Hydroperoxyoct-3-ene (1-Hyp)**

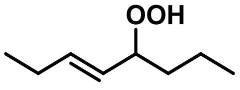

(*Z*)-4-octene (1.12 g, 10.0 mmol) was reacted according to GP2 in acetonitrile. ^{1}H NMR analysis showed quantitative conversion to 1-Hyp. The reaction solution was used in the following step without further purification. The reaction of (*E*)-4-octene (0.11 g, 1.0 mmol) following GP2 in acetonitrile also gave quantitative formation of 1-Hyp.

^{1}H NMR (300 MHz, $CDCl_3$, ppm): δ = 7.71 (br s, 1H), 5.83 (dt, *J* = 15.5, 6.2 Hz, 1H), 5.37 (m, 1H), 4.29 (dt, *J* = 8.3, 6.4 Hz, 1H), 2.18–2.04 (m, 2 H), 1.51–1.28 (m, 4 H), 1.02 (t, *J* = 7.5 Hz, 3H), 0.91 (t, *J* = 7.3, 3H). **^{13}C NMR** (100 MHz, $CDCl_3$, ppm): δ = 138.7, 127.6, 87.0, 34.7, 25.5, 18.7, 14.1, 13.5.
The spectral data are consistent with literature values.[31]

1-(3-Ethyloxiran-2-yl)butan-1-ol (1-EpAlc)

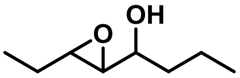

Following GP4, a crude solution of 1-Hyp (10.0 mmol) in acetonitrile was treated with $VO(acac)_2$ (26 mg). ^{1}H NMR analysis showed quantitative conversion to 1-EpAlc. A mixture of diastereomeric products (*syn*/*anti*) can be observed in the NMR spectra.

^{1}H NMR (300 MHz, $CDCl_3$, ppm): δ = 3.85–3.72 (m, 1H, *syn*) 3.50–3.33 (m, 1 H, *anti*), 3.06–2.95 (m, 1H, *syn*), 2.94–2.85 (m, 1H, *anti*), 2.79–2.76 (m, 1H, *syn*), 2.75–2.72 (m, 1H, *anti*), 1.67–1.34 (m, 6 H), 1.04–0.86 (m, 6 H) ppm. **^{13}C NMR** (151 MHz, $CDCl_3$, ppm): δ = 72.8, 71.4, 69.9, 68.3, 61.7, 61.6, 60.9, 60.8, 58.2, 56.9, 56.1, 54.9, 36.6, 35.8, 33.82, 33.81, 27.6, 26.6, 24.9, 24.8, 19.5, 19.4, 18. 7, 14.3, 14.2, 14.1, 10.0, 9.9, 9.84, 9.77 ppm.

4-(1-Hydroxypropyl)-5-propyl-1,3-dioxolan-2-one (1-CC)

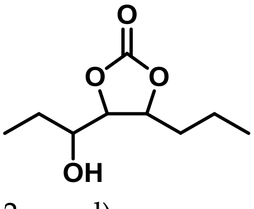

Following GP5, crude 1-EpAlc (4.2 mmol) was used as starting material. After flash chromatography on silica with pentane/ethyl acetate (9/1 to 4/1) as eluent, 1-CC was obtained as yellow oil (0.73 g, 92 % yield).

^{1}H NMR (300 MHz, $CDCl_3$, ppm): δ = 4.75–4.57 (m, 1H), 4.16–4.10 (m, 1H), 3.88–3.73 (m, 1H), 2.22 (br s, 1H), 1.78–1.37 (m, 6H), 1.06–0.92 (m, 6H). **^{13}C NMR** (101 MHz, $CDCl_3$, ppm): δ = 154.9, 83.2, 83.1, 80.3, 80.2, 79.0, 77.9, 72.3, 70.6, 70.0, 37.1, 36.5, 36.3, 35.0, 34.0, 31.1, 28.0, 27.4,

27.3, 26.2, 25.2, 22.5, 19.1, 18.8, 18.7, 18.14, 18.08, 18.0, 14.0, 13.8, 9.9, 8.9. **IR** (ATR, cm^{-1}): 1778 (C=O).

Reaction sequence for methyl oleate (2)

Methyl *trans*-9-hydroperoxyoctadec-10-enoate and
Methyl *trans*-10-hydroperoxyoctadec-8-enoate (2-Hyp)

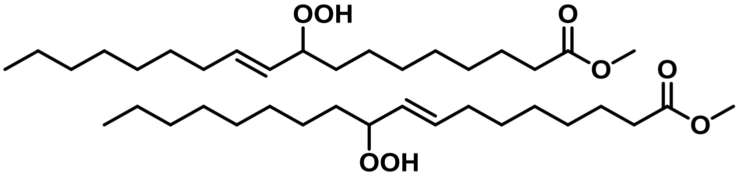

Methyl oleate (2.96 g, 10.0 mmol) was reacted according to GP1. ^{1}H NMR analysis showed quantitative conversion to 2-Hyp. The crude reaction mixture was used in the following step without further purification.

^{1}H NMR (300 MHz, $CDCl_3$, ppm): δ = 8.06 (d, J = 18.6 Hz, 1H), 5.81–5.64 (m, 1H), 5.42–5.28 (m, 1H), 4.27–4.20 (m, 1H), 3.64 (s, 3H), 2.33–2.23 (m, 2H), 2.11–1.99 (m, 2H), 1.69–1.16 (m, 22H), 0.91–0.81 (m, 3H). **^{13}C NMR** (126 MHz, $CDCl_3$, ppm): δ = 137.3, 136.8, 129.0, 128.6, 87.1, 87.1, 51.7, 51.6, 34.2, 34.1, 32.6, 32.5, 32.5, 32.3, 29.6, 29.6, 29.39, 29.35, 29.24, 29.19, 29.15, 29.1, 28.9, 28.84, 28.76, 25.5, 25.3, 24.98, 24.95, 22.8, 14.2. **GC-MS** (EI): m/z (%): 281 (18), 211 (2) [M^+–H_2O–(C_2H_4COOMe)], 167 (24), 153 (13), 137 (16), 97 (21), 81 (25), 67 (36), 55 (58).

The spectral data are consistent with literature values.[68]

Methyl 9-(3-heptyloxiran-2-yl)-9-hydroxynonanoate and
Methyl 7-(3-(-1-hydroxynonyl)oxiran-2-yl)heptanoate (2-EpAlc)

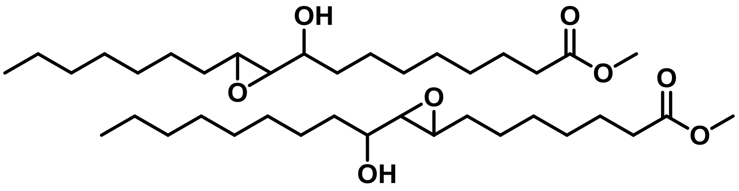

Following GP3, crude 2-Hyp (10.0 mmol) was treated with VO(acac)$_2$ (26 mg) in acetonitrile. ^{1}H NMR analysis showed quantitative conversion to 2-EpAlc. A mixture of diastereomeric products (*syn*/*anti: 1*/*1*) can be observed in the NMR spectra. A fraction of the product mixture was separated by flash chromatography on silica with pentane/ethyl acetate as eluent yielding the clean *syn*- and *anti*-diastereomers.

^{1}H NMR (300 MHz, $CDCl_3$, ppm): δ = 3.81–3.73 (m, 1H, *syn*), 3.64 (s, 3H), 3.48–3.37 (m, 1H, *anti*), 3.01–2.94 (m, 1H, *syn*), 2.87 (td, J = 5.5, 2.3 Hz, 1H, *anti*), 2.75–2.74 (m, 1H, *syn*), 2.70 (dd, J = 5.3, 2.3 Hz, 1H, *anti*), 2.32–2.27 (m, 2H), 2.09–1.98 (m, 1H), 1.71–1.19 (m, 24H), 0.87 (t, J = 6.6 Hz). **^{13}C NMR** (101 MHz, $CDCl_3$, ppm): δ = 174.3, 71.4, 68.7, 68.6, 61.92, 61.88, 61.0, 57.0, 56.9, 55.1, 55.0, 51.40, 51.38, 34.3, 34.2, 34.0, 33.9, 33.6, 33.5, 31.8, 31.7, 31.60, 31.57, 31.52, 31.49, 29.64, 29.58, 29.5, 29.40, 29.35, 29.32, 29.31, 29.2, 29.14, 29.09, 29.00, 28.98, 28.9, 26.0, 25.9, 25.8, 25.7, 25.28, 25.25, 25.2, 24.82, 24.75, 22.61, 22.57, 14.04, 14.01.

Methyl *syn*-9-(3-heptyloxiran-2-yl)-9-hydroxynonanoate and
Methyl *syn*-7-(3-(-1-hydroxynonyl)oxiran-2-yl)heptanoate (2 *syn*-EpAlc)

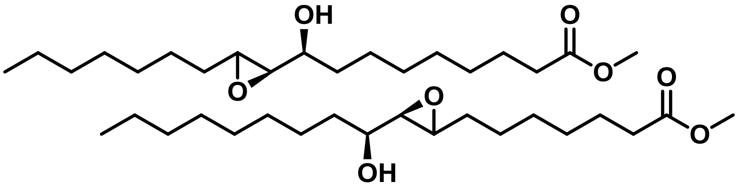

^{1}H NMR (300 MHz, $CDCl_3$, ppm): δ = 3.81–3.73 (m, 1H), 3.66 (s, 3H), 3.01–2.94 (m, 1H), 2.75–2.74 (m, 1H), 2.32–2.27 (m, 2H), 1.88 (br s, 1H), 1.71–1.19 (m, 24H), 0.87 (t, *J* = 6.6 Hz). **^{13}C NMR** (101 MHz, $CDCl_3$, ppm): δ = 174.4, 174.3, 68.72, 68.65, 61.1, 55.11, 55.04, 51.60, 51.58, 34.2, 34.1, 33.7, 33.6, 32.0, 31.9, 31.74, 31.66, 29.8, 29.63, 29.57, 29.5, 29.4, 29.32, 29.26, 29.2, 29.1, 26.2, 26.0, 25.44, 25.37, 25.0, 24.9, 22.79, 22.75, 14.2. **GC-MS** (EI): m/z (%): 207 (30), 187 (12), 155 (47) [M^+–H_2O–(C_8H_{16}COOMe)], 141 (70), 111 (23), 96 (42), 95 (60), 83 (48), 81 (65), 69 (54), 67 (64), 55 (100).

Methyl *anti*-9-(3-heptyloxiran-2-yl)-9-hydroxynonanoate and
Methyl *anti*-7-(3-(-1-hydroxynonyl)oxiran-2-yl)heptanoate (2 *anti*-EpAlc)

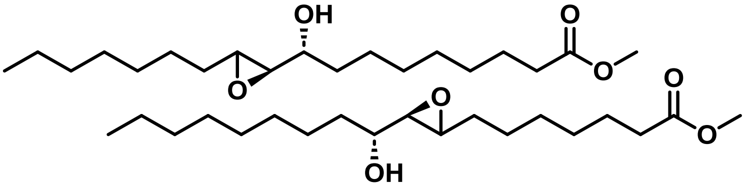

^{1}H NMR (300 MHz, $CDCl_3$, ppm): δ = 3.66 (s, 3H), 3.50–3.37 (m, 1H), 2.94–2.84 (m, 1H), 2.71 (dd, *J* = 5.1, 2.3 Hz, 1H), 2.30 (t, *J* = 7.5 Hz, 2H), 1.89 (br s, 1H), 1.70–1.49 (m, 8H), 1.48–1.99 (m, 16H), 0.87 (t, *J* = 6.5 Hz, 2H). **^{13}C NMR** (101 MHz, $CDCl_3$, ppm): δ = 174.3, 71.5, 61.9, 57.1, 57.0, 51.6, 34.6, 34.5, 34.20, 34.15, 32.0, 31.9, 31.8, 31.7, 29.7, 29.6, 29.5, 29.4, 29.32, 29.27, 29.2, 29.1, 26.1, 25.9, 25.43, 25.35, 25.0, 24.9, 22.8, 22.8, 14.2. **GC-MS** (EI): m/z (%): 187 (8), 173 (17), 155 (25), 141 (100), 95 (48), 81 (29), 57 (42), 55 (58).

Methyl 9-(5-heptyl-2-oxo-1,3-dioxolan-4-yl)-9-hydroxynonanoate and regioisomers **(2-CC)**

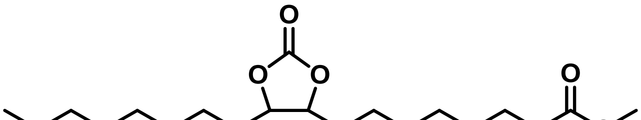

Following GP5, crude 2-EpAlc (4.2 mmol) was used as starting material. After flash chromatography on silica with pentane/ethyl acetate (9/1 to 2/1) as eluent, 2-CC was obtained as yellow oil (1.41 g, 90 % yield).

^{1}H NMR (300 MHz, $CDCl_3$, ppm): δ = 4.68–4.58 (m, 1H), 4.11–4.05 (m, 1H), 3.87–3.78 (m, 1H), 3.65 (s, 3H), 2.29 (t, *J* = 7.5 Hz, 2H), 1.81–1.12 (m, 24H), 0.86 (t, *J* = 6.4 Hz, 3H). **^{13}C NMR**

(101 MHz, $CDCl_3$, ppm): δ = 174.5, 155.0, 83.6, 78.0, 70.8, 51.6, 35.0, 34.1, 32.1, 32.0, 31.8, 29.5, 29.31, 29.29, 29.2, 29.03, 28.98, 28.9, 25.4, 24.9, 24.6, 22.74, 22.70, 14.2. **GC-MS** (EI): m/z (%): 207 (35), 173 (37), 155 (14), 141 (100), 95 (54), 83 (18), 69 (28), 55 (34). **IR** (ATR, cm^{-1}): 1794,1738 (C=O).

Reaction sequence for oleyl alcohol (3)

***trans*-9-Hydroperoxyoctadec-10-en-1-ol** and ***trans*-10-Hydroperoxyoctadec-8-en-1-ol (3-Hyp)**

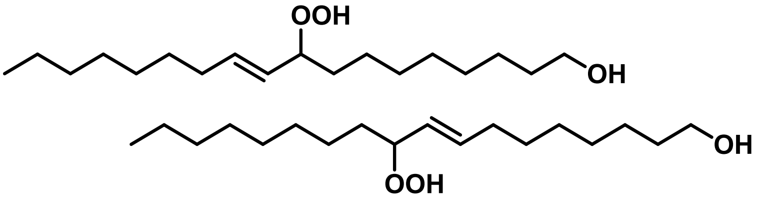

Oleyl alcohol (0.81 g, 3.0 mmol) was reacted according to GP1. ^1H NMR analysis showed quantitative conversion to 3-Hyp. The reaction mixture was used in the following step without further purification.

^1H NMR (300 MHz, $CDCl_3$, ppm): δ = 8.56 (s, br, 1H), 5.81–5.72 (m, 1H), 5.36 (dd, J = 15.4, 8.2, 1H), 4.25–4.18 (m, 1H), 3.64 (t, J = 6.7, 2H), 2.15–1.98 (m, 2H), 1.64–1.18 (m, 24H), 0.85 (t, J = 6.4 Hz, 3H). **^{13}C NMR** (101 MHz, $CDCl_3$, ppm): δ = 137.1, 136.9, 128.9, 128.7, 87.1, 63.2, 63.1, 32.8, 32.7, 32.59, 32.57, 32.5, 32.4, 31.98, 31.95, 29.82, 29.78, 29.74, 29.66, 29.6, 29.5, 29.41, 29.35, 29.3, 29.23, 29.17, 29.1, 29.0, 25.78, 25.75, 25.5, 25.4, 22.81, 22.78, 14.2.

1-(3-Heptyloxiran-2-yl)nonane-1,9-diol and **1-(3-(7-Hydroxyheptyl)oxiran-2-yl)nonan-1-ol (3-EpAlc)**

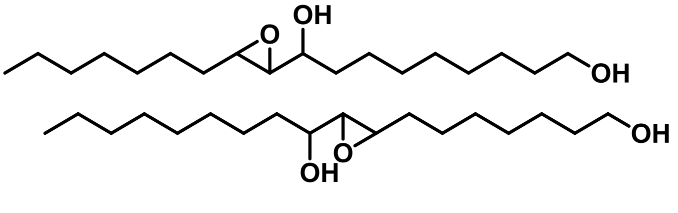

Following GP3, crude 3-Hyp (3.0 mmol) was treated with $VO(acac)_2$ (8.0 mg) in acetonitrile. ^1H NMR analysis showed quantitative conversion to 3-EpAlc. A mixture of diastereomeric products (*syn*/*anti: 2/3*) can be observed in the NMR spectra.

^1H NMR (300 MHz, $CDCl_3$, ppm): δ = 3.81–3.73 (m, 1H, *syn*), 3.62 (t, J = 6.6 Hz, 2H), 3.46–3.40 (m, 1H, *anti*), 2.98 (td, J = 5.5, 2.2 Hz, 1H, *syn*), 2.89 (td, J = 6.7, 2.2 Hz, 1H, *anti*), 2.76–2.74 (m, 1H, *syn*), 2.71 (dd, J = 5.2, 2.3 Hz, 1H, *anti*), 1.99 (s, br, 1H), 1.62–1.21 (m, 26H), 0.92–0.82 (m, 3H). **^{13}C NMR** (101 MHz, $CDCl_3$, ppm): δ = 71.5, 68.73, 68.68, 63.2, 63.10, 63.06, 62.01, 61.99, 61.2, 57.2, 57.1, 55.18, 55.16, 34.51, 34.48, 33.9, 33.70, 33.65, 32.91, 32.86, 32.8, 32.1, 32.0, 31.9, 31.8, 31.74, 31.71, 29.81, 29.78, 29.74, 29.70, 29.63, 29.59, 29.56, 29.5, 29.44, 29.40, 29.37, 29.3, 29.23, 26.15, 26.1, 26.0, 25.9, 25.82, 25.75, 25.4, 22.81, 22.78, 22.7, 14.22, 14.19.

4-(1-Hydroxyoctyl)-5-(8-hydroxyoctyl)-1,3-dioxolan-2-one and regioisomers **(3-CC)**

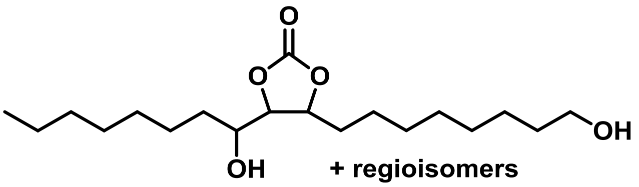

Following GP5, crude 3-EpAlc (1.8 mmol) was used as starting material. After flash chromatography on silica with pentane/ethyl acetate (9/1 to 2/1) as eluent, 3-CC was obtained as yellow oil (0.53 g, 85 % yield).

^{1}H NMR (300 MHz, $CDCl_3$, ppm): δ = 4.67–4.56 (m, 1H), 4.16–4.13 (m, 1H, *diast.*), 4.11–4.08 (m, 1H, *diast.*), 3.87–3.82 (m, 1H, *diast.*), 3.63 (t, J = 6.6 Hz, 2 H), 3.60–3.55 (m, 1H, *diast.*), 1.90–1.80 (m, 2H), 1.78–1.70 (m, 1H), 1.69–1.62 (m, 2H), 1.59–1.44 (m, 5H), 1.40–1.20 (m, 19H), 0.88 (t, J = 6.7 Hz, 3H). **^{13}C NMR** (101 MHz, $CDCl_3$, ppm): δ = 155.1, 84.04, 84.01, 83.8, 79.13, 79.08, 78.31, 78.28, 71.3, 71.1, 71.0, 63.4, 63.3, 35.34, 35.28, 34.6, 34.1, 33.33, 33.30, 33.1, 33.0, 32.3, 32.2, 32.1, 29.8, 29.7, 29.6, 29.5, 29.4, 26.1, 26.02, 25.96, 25.9, 25.81, 25.75, 25.6, 25.0, 24.9, 24.8, 23.03, 22.99, 14.5. **HRMS** (ESI): *m/z* calculated for $C_{19}H_{36}O_5$+Na^+: 367.2460 $[M+Na]^+$; found 367.2449. **IR** (ATR, cm^{-1}): 1778 (C=O).

Reaction sequence for oleic acid (4)

***trans*-9-Hydroperoxyoctadec-10-enoic acid** and ***trans*-10-Hydroperoxyoctadec-8-enoic acid (4-Hyp)**

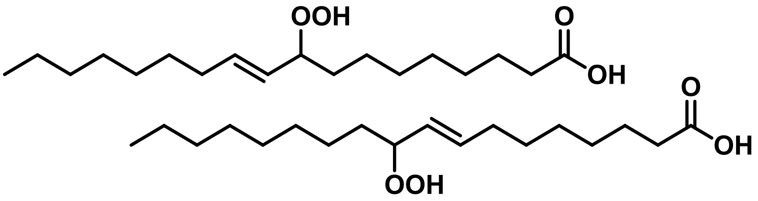

Oleic acid (0.85 g, 3.0 mmol) was reacted according to GP1 in dichloromethane. ^{1}H NMR analysis showed quantitative conversion to 4-Hyp. The reaction solution was used in the following step without further purification.

^{1}H NMR (300 MHz, $CDCl_3$, ppm): δ = 5.81–5.69 (m, 1H), 5.39–5.30 (m, 1H), 4.30–4.23 (m, 1H), 2.38–2.30 (m, 2H), 2.09–2.07 (m, 2H), 1.69–1.56 (m, 3H), 1.48–1.19 (m, 19H), 0.92–0.83 (m, 3H). **^{13}C NMR** (101 MHz, $CDCl_3$, ppm): δ = 180.1, 180.0, 137.4, 136.9, 128.9, 128.5, 87.21, 87.18, 34.12, 34.07, 32.6, 32.51, 32.46, 32.3, 31.98, 31.95, 29.7, 29.56, 29.39, 29.36, 29.3, 29.19, 29.16, 29.1, 28.9, 28.83, 28.75, 25. 5, 25.4, 24.73, 24.68, 22.8, 14.2.

The spectral data are consistent with literature values.[69]

9-(3-Heptyloxiran-2-yl)-9-hydroxynonanoic acid and
7-(3-(1-Hydroxynonyl)oxiran-2-yl)-heptanoic acid (4-EpAlc)

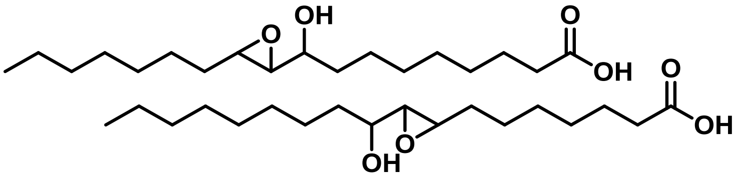

Following GP4, a crude solution of 4-Hyp (3.0 mmol) in dichloromethane was treated with VO(acac)$_2$ (8.0 mg). ^{1}H NMR analysis showed quantitative conversion to 4-EpAlc. A mixture of diastereomeric products (*syn/anti: 3/2*) can be observed in the NMR spectra. 4-EpAlc was isolated by flash chromatography on silica with pentane/ethyl acetate as eluent as off-white solid (0.80 g, 85 % yield).

^{1}H NMR (300 MHz, CDCl$_3$, ppm): δ = 3.84–3.73 (m, 1H, *syn*), 3.50–3.38 (m, 1H, *anti*), 3.05 – 2.96 (m, 1H, *syn*), 2.95–2.87 (m, 1H, *anti*), 2.79–2.70 (m, 1H), 2.33 (t, *J* = 7.3 Hz, 2H), 1.70–1.17 (m, 24H), 0.87 (t, *J* = 6.4 Hz, 3H). **^{13}C NMR** (151 MHz, CDCl$_3$, ppm): δ = 179.7, 179.6, 71.5, 71.4, 68.7, 68.6, 62.14, 62.10, 61.3, 61.2, 57.3, 57.2, 55.3, 55.2, 34. 5, 34.4, 34.10, 34.08, 34.05, 33.7, 33.6, 33.5, 31.99, 31.96, 31.9, 31.73, 31.70, 31.66, 31.64, 31.61, 29.82, 29.79, 29.75, 29.7, 29.63, 29.60, 29.53, 29.48, 29.4, 29.33, 29.31, 29.23, 29.20, 29.11, 29.08, 29.0, 26.13, 26.05, 25.9, 25.9, 25.43, 25.36, 25.3, 25.2, 24.8, 24.70, 24.65, 22.78, 22.75, 14.23, 14.21.

Reaction sequence for cardanyl acetate (5)

***trans*-3-(8-Hydroperoxypentadec-9-en-1-yl)phenyl acetate** and
***trans*-3-(9-Hydroperoxy-pentadec-7-en-1-yl)phenyl acetate (5-Hyp)**

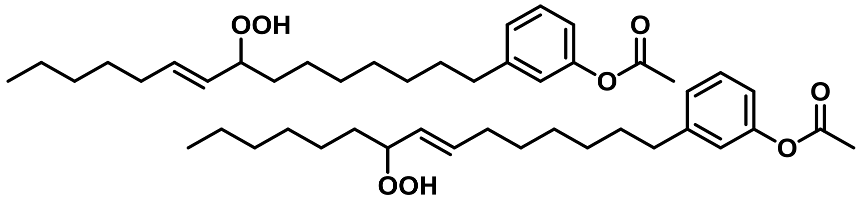

Cardanyl acetate (0.5 g, 1.5 mmol) was reacted according to GP2 in acetonitrile. ^{1}H NMR analysis showed quantitative conversion to 5-Hyp. The reaction solution was used in the following step without further purification.

^{1}H NMR (300 MHz, CDCl$_3$, ppm): δ = 7.66 (br s, 1H), 7.33–7.23 (m, 1H), 7.03 (d, *J* = 7.8 Hz, 1H), 6.93–6.86 (m, 2H), 5.85–5.69 (m, 1H), 5.42–5.30 (m, 1H), 4.30 – 4.23 (m, 1H), 2.63 – 2.57 (m, 2H), 2.29 (s, 3H), 2.12–1.99 (m, 2H), 1.70–1.20 (m, 18H), 0.93–0.83 (m, 3H). **^{13}C NMR** (101 MHz, CDCl$_3$, ppm): δ = 150.7, 144.8, 137.3, 137.1, 129.3, 128.8, 128.6, 126.1, 121.6, 118.88, 118.85, 87.23, 87.21, 35.8, 32.60, 32.57, 32.44, 32.41, 31.9, 31.5, 31.24, 31.18, 29.5, 29.4, 29.33, 29.25, 29.12, 29.08, 29.0, 28.9, 25.9, 22.7, 22.6, 21.3, 14.2.

3-(8-Hydroxy-8-(3-pentyloxiran-2-yl)octyl)phenyl acetate and
3-(6-(3-(1-Hydroxyheptyl)-oxiran-2-yl)hexyl)phenyl acetate (5-EpAlc)

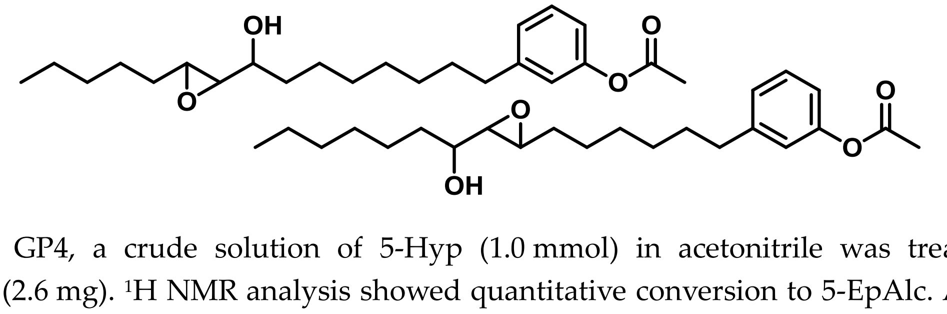

Following GP4, a crude solution of 5-Hyp (1.0 mmol) in acetonitrile was treated with VO(acac)$_2$ (2.6 mg). ^{1}H NMR analysis showed quantitative conversion to 5-EpAlc. A mixture of diastereomeric products (*syn/anti: 1/1*) can be observed in the NMR spectra.

^{1}**H NMR** (300 MHz, $CDCl_3$, ppm): *δ* = 7.30−7.23 (m, 1H), 7.05–7.02 (m, 1H), 6.90–6.89 (m, 2H), 3.83−3.73 (m, 1H, *syn*), 3.49−3.39 (m, 1H, *anti*), 3.03−2.95 (m, 1H, *syn*), 2.93−2.86 (m, 1H *anti*), 2.78−2.69 (m, 1H), 2.60 (t, *J* = 7.8 Hz, 2H), 2.29 (s, 3H), 1.80 (s, br, 1H), 1.69−1.18 (m, 20H), 0.93−0.84 (m, 3H). ^{13}**C NMR** (151 MHz, $CDCl_3$, ppm): *δ* = 169.7, 150.5, 144.7, 129.3, 126.1, 121.6, 118.9, 71.5, 68.69, 68.66, 61.88, 61.86, 61.1, 57.12, 57.05, 55.10, 55.05, 35.82, 35.78, 34.60, 34.56, 33.7, 33.6, 31.9, 31.7, 31.2, 31.1, 29.7, 29.6, 29.5, 29.4, 29.4, 29.3, 29.3, 29. 2, 26.14, 26.07, 25.41, 25.39, 22.7, 21.3, 14.2, 14.1.

3-(7-(5-Hexyl-2-oxo-1,3-dioxolan-4-yl)-7-hydroxyheptyl)phenyl acetate and regioisomers **(5-CC)**

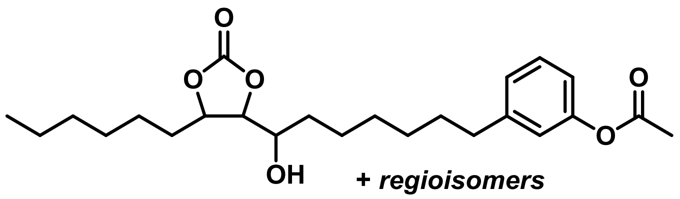

Following GP5, crude 5-EpAlc (0.6 mmol) was used as starting material. After flash chromatography on silica with pentane/ethyl acetate (9/1 to 4/1) as eluent, O-CC was obtained as yellow oil (0.23 g, 93 % yield).

^{1}**H NMR** (300 MHz, $CDCl_3$, ppm): *δ* = 7.29–7.25 (m, 1H), 7.04–7.03 (m, 1H), 6.90–6.88 (m, 2H), 4.65–4.59 (m, 1H), 4.10–4.06 (m, 1H), 3.86–3.78 (m, 1H), 2.62–2.59 (m, 2H), 2.29 (s, 3H), 1.73–1.59 (m, 5H), 1.40–1.25 (m, 19H), 0.91–0.87 (m, 3H). ^{13}**C NMR** (101 MHz, $CDCl_3$, ppm): *δ* = 151.1, 144.9, 144.7, 129.59, 129.56, 126.4, 121.9, 121.8, 119.20, 119.16, 119.1, 83.7, 78.3, 71.2, 71.0, 36.02, 35.94, 35.34, 35.30, 35.27, 35.2, 32.4, 32.3, 32.2, 32.1, 32.0, 31.7, 31.4, 31.3, 31.2, 29.54, 29.52, 29.45, 29.3, 29.1, 25.7, 25.6, 25.5, 25.4, 24.92, 24.87, 24.8, 24.6, 23.0, 22.9, 22.8, 21.6, 14.44, 14.38. **IR** (ATR, cm^{-1}): 1796, 1767 (C=O).

Reaction sequence for oleyl acrylate (6)

***trans*-9-Hydroperoxyoctadec-10-en-1-yl acrylate** and
***trans*-10-Hydroperoxyoctadec-8-en-1-yl acrylate (6-Hyp)**

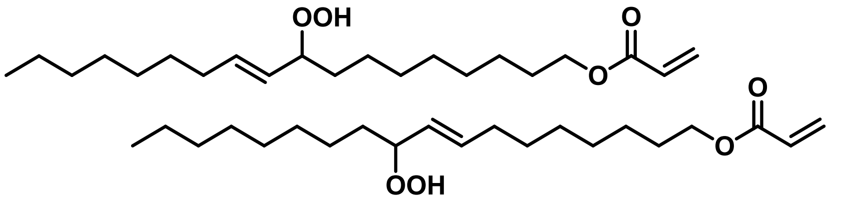

Oleyl acrylate (0.97 g, 3.0 mmol) was reacted according to GP2 in acetonitrile. ^{1}H NMR analysis showed quantitative conversion to 6-Hyp. The reaction solution was used in the following step without further purification. Hydroquinone monomethyl ether (15 ppm) was added as stabilizer to prevent radical polymerization.

^{1}**H NMR** (300 MHz, $CDCl_3$, ppm): *δ* = 7.75–7.66 (m, 1H), 6.40 (d, *J* = 17.3 Hz, 1H), 6.12 (dd, *J* = 17.3, 10.4 Hz, 1H), 5.85–5.70 (m, 2H), 5.43–5.30 (m, 1H), 4.30–4.23 (m, 1H), 4.15 (t, *J* = 6.7 Hz, 2H), 2.11–2.04 (m, 2H), 1.72−1.56 (m, 4H), 1.49−1.18 (m, 20H), 0.88 (t, *J* = 6.4 Hz, 3H). ^{13}**C NMR** (151 MHz, $CDCl_3$, ppm): *δ* = 166.5, 137.4, 137.0, 130.7, 130.6, 128.84, 128.77, 128.75, 128.6, 87.2,

64.83, 64.79, 32.59, 32.56, 32.5, 32.4, 32.00, 31.96, 29.7, 29.6, 29.54, 29.47, 29.4, 29.3, 29.19, 29.15, 29.12, 29.06, 28.72, 28.70, 26.01, 25.97, 25.5, 25.4, 22.8, 14.2.

9-(3-Heptyloxiran-2-yl)-9-hydroxynonyl acrylate and
7-(3-(1-Hydroxynonyl)oxiran-2-yl)-heptyl acrylate (6-EpAlc)

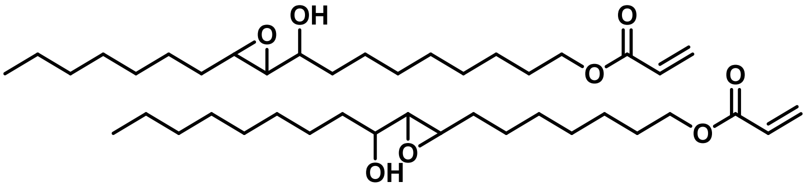

Following GP4, a crude solution of 6-Hyp (3.0 mmol) in acetonitrile was treated with $VO(acac)_2$ (8.0 mg). ^1H NMR analysis showed quantitative conversion to 6-EpAlc. A mixture of diastereomeric products (*syn/anti: 5/4*) can be observed in the NMR spectra.

^{1}H NMR (300 MHz, $CDCl_3$, ppm): δ = 6.39 (dd, *J* = 17.3, 1.6 Hz, 1H), 6.11 (dd, *J* = 17.3, 10.4 Hz, 1H), 5.81 (dd, *J* = 10.4, 1.6 Hz, 1H), 4.14 (t, *J* = 6.7 Hz, 2H), 3.83–3.75 (m, 1H, *syn*), 3.49–3.39 (m, 1H, *anti*), 3.01–2.96 (m, 1H, *syn*), 2.92–2.87 (m, 1H, *anti*), 2.76–2.74 (m, 1H, *syn*), 2.73–2.70 (m, 1H, *anti*), 1.84 (s, br, 1H), 1.72–1.21 (m, 26H), 0.93–0.83 (m, 3H). **^{13}C NMR** (151 MHz, $CDCl_3$, ppm): δ = 166.5, 130.62, 130.59, 128.77, 128.75, 71.5, 68.68, 68.65, 64.8, 64.7, 61.90, 61.87, 61.1, 57.1, 57.0, 55.1, 55.0, 34.58, 34.55, 33.7, 33.6, 32.0, 31.9, 31.78, 31.75, 31.7, 29.82, 29.75, 29.7, 29.6, 29.52, 29.49, 29.41, 29.38, 29.33, 29.30, 29.26, 28.73, 28.70, 26.2, 26.1, 26.03, 25.95, 25.5, 25.43, 25.39, 22.80, 22.76, 14.24, 14.21.

8-(5-(1-Hydroxyoctyl)-2-oxo-1,3-dioxolan-4-yl)octyl acrylate and regioisomers **(6-CC)**

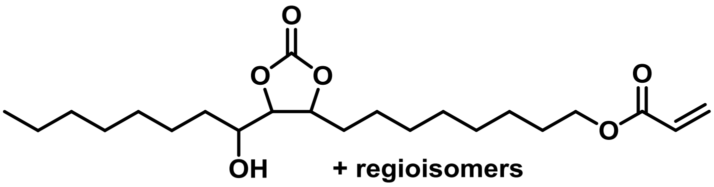

Following GP5, crude 6-EpAlc (1.2 mmol) was used as starting material. After flash chromatography on silica with pentane/ethyl acetate (9/1 to 2/1) as eluent, O-CC was obtained as yellow oil (0.42 g, 88 % yield). Hydroquinone monomethyl ether (15 ppm) was added as stabilizer to prevent radical polymerization.

^{1}H NMR (300 MHz, $CDCl_3$, ppm): δ = 6.33 (d, *J* = 17.3 Hz, 1H), 6.05 (dd, *J* = 17.3, 10.4 Hz, 1H), 5.75 (d, *J* = 10.4 Hz, 1H), 4.62–4.56 (m, 1H), 4.10–4.02 (m, 3H), 3.82–3.76 (m, 1H), 2.55–2.54 (m, 1H), 1.62–1.21 (m, 26H), 0.83–0.79 (m, 3H). **^{13}C NMR** (101 MHz, $CDCl_3$, ppm): δ = 166.5, 155.0, 130.7, 128.7, 83.6, 78.0, 70.8, 64.7, 35.0, 32.1, 31.8, 29.5, 29.3, 29.2, 29.1, 29.0, 28.7, 25.9, 25.4, 24.6, 22.7, 14.2. **IR** (ATR, cm^{-1}): 1797, 1722 (C=O).

Reaction sequence for trimethylene glycol dioleate (7)

Propane-1,3-diyl (10-*trans*,10'-*trans*)-bis(9-hydroperoxyoctadec-10-enoate) and isomers **(7-Hyp)**

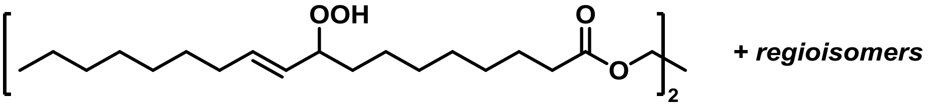

Trimethylene glycol dioleate (1.8 g, 3.0 mmol) was reacted according to GP2 in dichloromethane. ^{1}H NMR analysis showed quantitative conversion to 7-Hyp. The reaction solution was used in the following step without further purification.

^{1}H NMR (300 MHz, $CDCl_3$, ppm): δ = 7.82 (d, *J* = 29.6 Hz, 1H), 5.76 (dq, *J* = 15.5, 6.5 Hz, 1H), 5.36 (dd, *J* = 15.5, 8.2 Hz, 1H), 4.26 (dt, *J* = 7.3, 6.8 Hz, 2H), 4.15 (t, *J* = 6.3 Hz, 4 H), 2.30 (td, *J* = 7.5, 2.1 Hz, 4H), 2.13–1.91 (m, 4H), 1.68–1.55 (m, 4H), 1.47–1.17 (m, 40H), 0.93–0.82 (m, 6H). **^{13}C NMR** (151 MHz, $CDCl_3$, ppm): δ = 174.00, 173.99, 137.3, 136.8, 129.0, 128.6, 87.2, 87.1, 61.0, 34.4, 34.3, 32.61, 32.57, 32.5, 32.3, 32.00, 31.96, 29.8, 29.7, 29.6, 29.42, 29.37, 29.27, 29.26, 29.20, 29.19, 29.1, 29.0, 28.9, 28.8, 28.1, 25.5, 25.4, 25.01, 24.97, 22.80, 22.79, 14.3.

Propane-1,2-diyl bis(9-(3-heptyloxiran-2-yl)-9-hydroxynonanoate) and regioisomers **(7-EpAlc)**

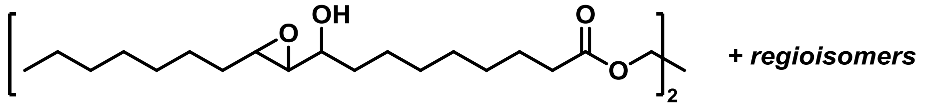

Following GP4, a crude solution of 7-Hyp (3.0 mmol) in dichloromethane was treated with $VO(acac)_2$ (8.0 mg). ^{1}H NMR analysis showed quantitative conversion to O-EpAlc. A mixture of diastereomeric products (*syn/anti: 8/7*) can be observed in the NMR spectra.

^{1}H NMR (300 MHz, $CDCl_3$, ppm): δ = 4.12 (t, *J* = 6.3 Hz, 4H), 3.79–3.69 (m, 1H, *syn*), 3.47–3.36 (m, 1H, *anti*), 3.02–2.92 (m, 1H, *syn*), 2.89–2.83 (m, 1H, *anti*), 2.75–2.67 (m, 2H), 2.28 (t, *J* = 7.4 Hz, 4H,), 1.94 (p, *J* = 6.3 Hz, 2H), 1.66 – 1.18 (m, 48H), 0.86 (t, *J* = 6.7 Hz, 6H). **^{13}C NMR** (151 MHz, $CDCl_3$, ppm): δ = 173.8, 173.8, 71.44, 71.37, 68.8, 68.7, 62.0, 61.9, 61.1, 61.0, 57.1, 57.0, 55.2, 55.1, 34.5, 34.4, 34.3, 34.2, 33.7, 33.6, 32.0, 31.8, 31.7, 31.71, 31.65, 31.6, 29.8, 29.73, 29.70, 29.66, 29.6, 29.53, 29.48, 29.46, 29.4, 29.34, 29.29, 29.27, 29.2, 29.12, 29.07, 28.1, 26.1, 26.0, 25.94, 25.87, 25.4, 25.34, 25.32, 25.0, 24.9, 22.74, 22.71, 14.19, 14.17.

7-CC

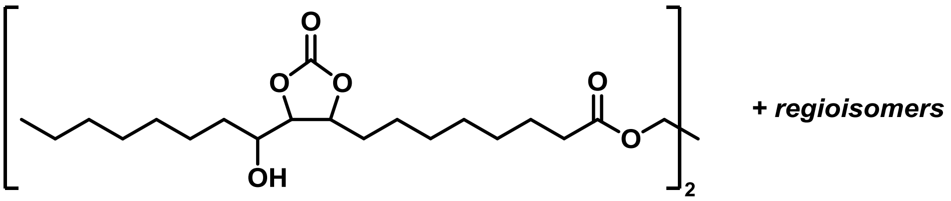

Following GP5, crude 7-EpAlc (1.8 mmol) was used as starting material. After flash chromatography on silica with pentane/ethyl acetate (9/1 to 1/1) as eluent, O-CC was obtained as yellow oil (1.2 g, 87 % yield).

^{1}H NMR (300 MHz, $CDCl_3$, ppm): δ = 4.60–4.57 (m, 2H), 4.09–4.07 (m, 4H), 4.04–4.02 (m, 2H), 2.27–2.21 (m, 4H), 1.92–1.89 (m, 2H), 1.62–1.56 (m, 8H), 1.33–1.18 (m, 2H), 0.82–0.80 (m, 6H). **^{13}C NMR** (101 MHz, $CDCl_3$, ppm): δ = 173.9, 155.0, 153.2, 83.5, 78.1, 70.8, 60.4, 35.1, 35.0, 34.4, 34.3, 33.9, 32.1, 32.0, 31.91, 31.85, 31.8, 29.81, 29.77, 29.7, 29.6, 29.5, 29.4, 29.3, 29.2, 29.0, 28.8, 28.1, 25.4, 25.2, 25.1, 24.90, 24.85, 24.7, 24.6, 22.80, 22.76, 22.7, 14.2. **IR** (ATR, cm^{-1}): 1799, 1731 (C=O).

Reaction sequence for triolein (8)

8-Hyp

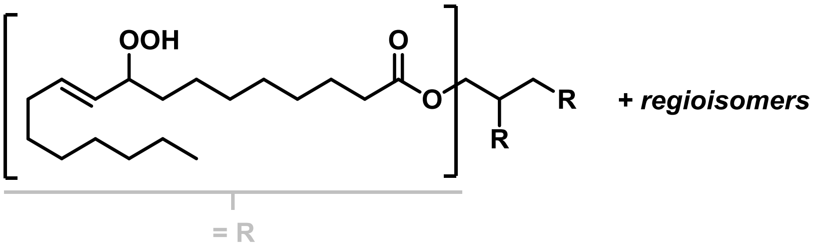

Triolein (2.7 g, 3.0 mmol) was reacted according to GP1. ^{1}H NMR analysis showed quantitative conversion to 8-Hyp. The reaction mixture was used in the following step without further purification.

^{1}H NMR (300 MHz, $CDCl_3$, ppm): δ = 7.85–7.71 (m, 3H), 5.75–5.63 (m, 3H), 5.30 (dd, *J* = 15.5, 8.1 Hz, 3H), 5.22–5.16 (m, 1H), 4.27–4.15 (m, 3H), 4.11–4.03 (m, 2H), 2.25 (t, *J* = 7.4 Hz, 1H), 2.05–1.95 (m, 5H), 1.61–1.46 (m, 10H), 1.36–1.14 (m, 57H), 0.81 (t, *J* = 6.5 Hz, 1H).

8-EpAlc

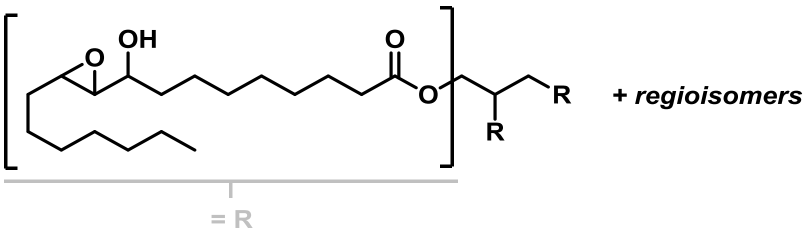

Following GP3, crude 8-Hyp (3.0 mmol) was treated with $VO(acac)_2$ (24 mg) in dichloromethane. ^{1}H NMR analysis showed quantitative conversion to 8-EpAlc. A mixture of diastereomeric products (*syn/anti: 4/5*) can be observed in the NMR spectra.

^{1}H NMR (300 MHz, $CDCl_3$, ppm): δ = 5.26–5.22 (m, 1H), 4.29–4.26 (m, 2H), 4.14–4.11 (m, 2H), 3.75–3.73+3.43–3.41 (m, 3H), 2.98–2.87 (m, 3H), 2.74–2.70 (m, 3H), 2.34–2.24 (m, 6H), 1.60–1.23 (m, 75H), 0.87–0.85 (m, 3H). **^{13}C NMR** (151 MHz, $CDCl_3$, ppm): δ = 173.7, 173.2, 71.7, 69.3, 69.1, 61.4, 57.4, 57.3, 55.4, 34.8, 34.6, 34.5, 34.4, 34.3, 34.0, 33.9, 32.31, 32.25, 32.23, 32.19, 32.15, 32.03, 32.01, 31.95, 30.09, 30.07, 30.01, 29.96, 29.90, 29.87, 29.77, 29.75, 29.64, 29.60, 29.58, 29.5, 29.4, 29.3, 26.42, 26.35, 26.2, 25.7, 25.2, 23.1, 23.0, 14.5.

8-CC

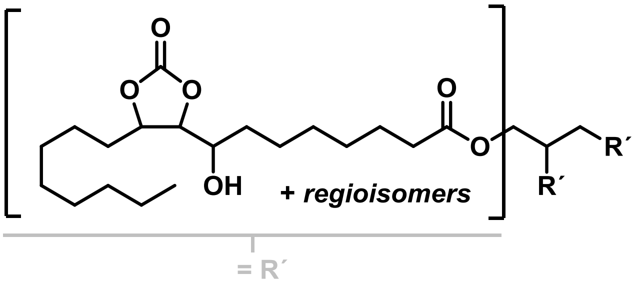

Following GP5, crude 8-EpAlc (2.4 mmol) was used as starting material. After flash chromatography on silica with pentane/ethyl acetate (9/1 to 1/1) as eluent, O-CC was obtained as orange oil (2.2 g, 83 % yield).

^{1}H NMR (300 MHz, $CDCl_3$, ppm): δ = 5.29–5.23 (m, 1H), 4.72–3.58 (m, 12H), 2.35–2.28 (m, 6H), 1.73–1.24 (m, 76H), 0.88–0.85 (m, 9H). **^{13}C NMR** (151 MHz, $CDCl_3$, ppm): δ = 173.3, 172.8, 154.6, 83.6, 83.4, 80.5, 78.8, 78.6, 78.1, 70.7, 70.4, 68.9, 68.6, 62.1, 34.94, 34.86, 34.2, 34.1, 34.0, 32.9, 32.0, 31.9, 31.8, 31.74, 31.66, 29.70, 29.66, 29.5, 29.4, 29.3, 29.2, 29.0, 28.9, 28.8, 25.7, 25.5, 25.3, 24.7, 24.6, 24.54, 24.48, 22.7, 22.6, 14.1. **IR** (ATR, cm^{-1}): 1796, 1738 (C=O).

6. NIPU Formation and Analysis

Reaction with solvent

8-CC (0.15 g, 0.13 mmol), hexamethylenediamine (0.019 g, 0.16 mmol) and DABCO (1.0 mg, 8.9 μmol) were dissolved in dichloromethane (3 mL) under vigorous stirring. The solution was poured into an aluminum pan, and the solvent was slowly evaporated. Afterwards, the reaction mixture was cured for 12 hours at 90 °C. An orange NIPU film (NIPU 1) was obtained. For DSC, the sample was stored at atmospheric conditions until the measurement.

Neat reaction under inert atmosphere

The following procedure was conducted in dry glassware under Schlenk conditions (N_2 atmosphere). 8-CC (0.15 g, 0.13 mmol), hexamethylenediamine (0.019 g, 0.16 mmol) and DABCO (1.0 mg, 8.9 μmol) were heated to 60 °C and stirred slowly for two hours to achieve a homogenous mixture. Then, the reaction mixture was cured for 12 hours at 90 °C. An orange NIPU film (NIPU 3) was obtained. For DSC, the sample was stored under nitrogen until the measurement.

Neat reaction under ambient atmosphere

8-CC (0.15 g, 0.13 mmol), hexamethylenediamine (0.019 g, 0.16 mmol) and DABCO (1.0 mg, 8.9 μmol) were heated to 60 °C and stirred slowly for two hours to achieve a homogenous mixture. Then, the reaction mixture was cured for 12 hours at 90 °C. An orange NIPU film (NIPU 2) was obtained. For DSC, the sample was stored at atmospheric conditions until the measurement.

The NIPU films were characterized by TGA (Figure S3) and DSC (Exemplary measurement in Figure S4). The Tg and gel content were determined (Table S1). IR-spectra of the monomer 8-CC (Figure S5) and the NIPU-films (Figure S6-S8) were recorded and compared (Table S2). The intensity of the C=O stretch vibration of the cyclic carbonate (1793 cm^{-1}) decreased, and characteristic bands of urethanes (1714 cm^{-1}, 1530 cm^{-1}) appeared.

Gel content

An aliquot of the NIPU was left to swell in dry THF for 24 h in a N_2 filled glove box. Afterwards, the residue was filtered and dried at 60 °C for 24 h under nitrogen atmosphere. The gel content equals the ratio of recovered to initial weight.

Test reaction with 1-hexylamine

8-CC (0.15 g, 0.13 mmol), 1-hexylamine (41 mg, 0.41 mmol, 1.2 equiv. per carbonate unit) and DABCO (1.0 mg, 8.9 μmol) were dissolved in dichloromethane (3 mL) under vigorous stirring. The solution was poured into an aluminum pan, and the solvent was slowly evaporated. Afterwards, the reaction mixture was cured for 12 hours at 90 °C. A dark oil was obtained. The IR spectrum of the product resembles the NIPUs (Figure S9), the gel content is 0 %. ^{1}H NMR analysis of this reaction is given in Figure S10.

Table S2. Gel content and T_g for the differently prepared NIPUs.

Sample	Preparation method	Tg [°C]	Gel content
1	Mixing in solution, ambient atmosphere	-4 °C	54 %
2	Neat, ambient atmosphere	+2 °C	84 %
3	Neat, inert atmosphere	-2 °C	93 %
Reaction product 8-CC with 1-hexylamine		n.d.	0 %

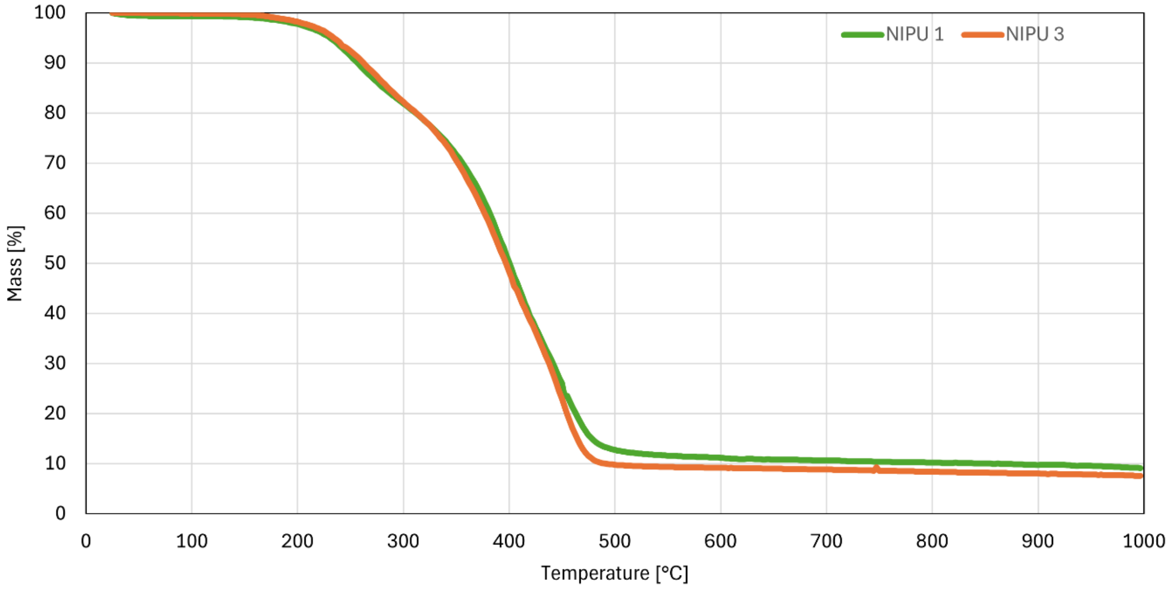

Figure S3. TGA curves of NIPU films 1 (green) and 3 (orange).

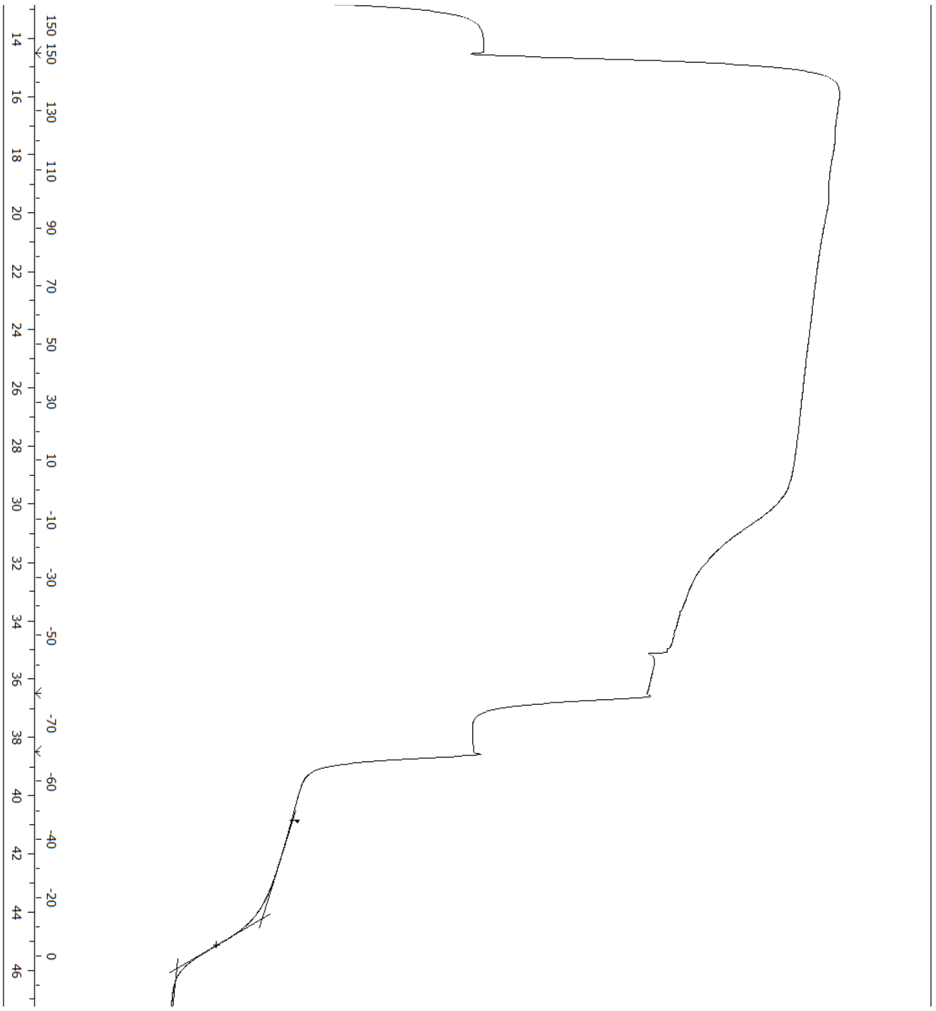

Figure S4. Exemplary DSC measurement of NIPU film 1.

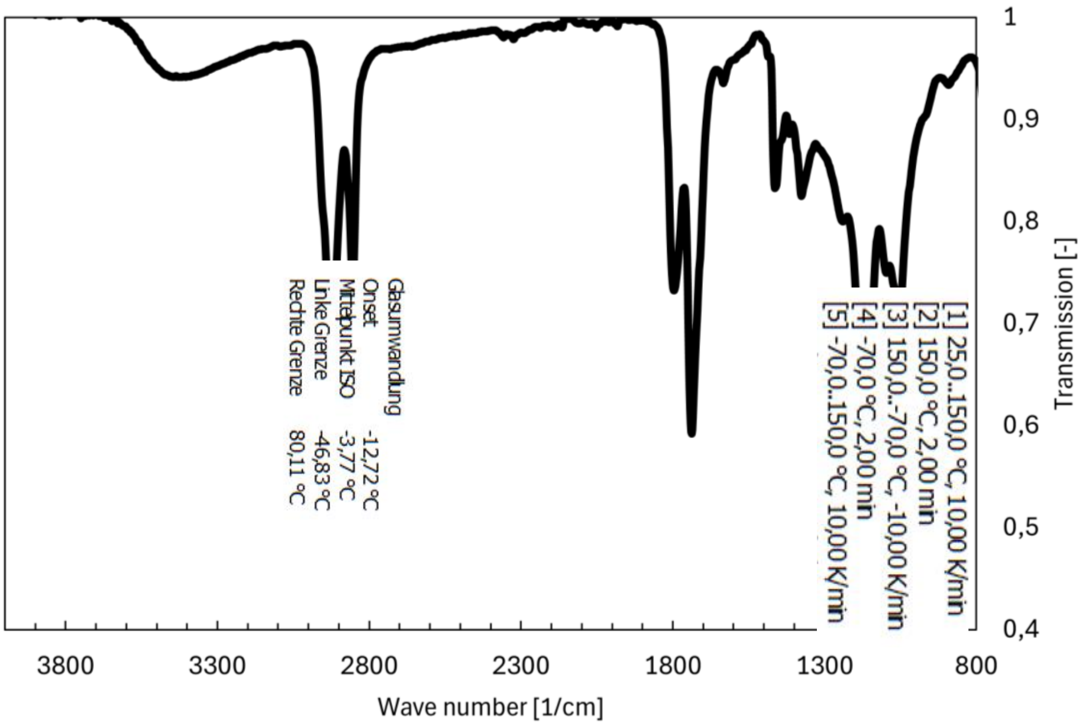

Figure S5. IR-spectrum of monomer 8-CC.

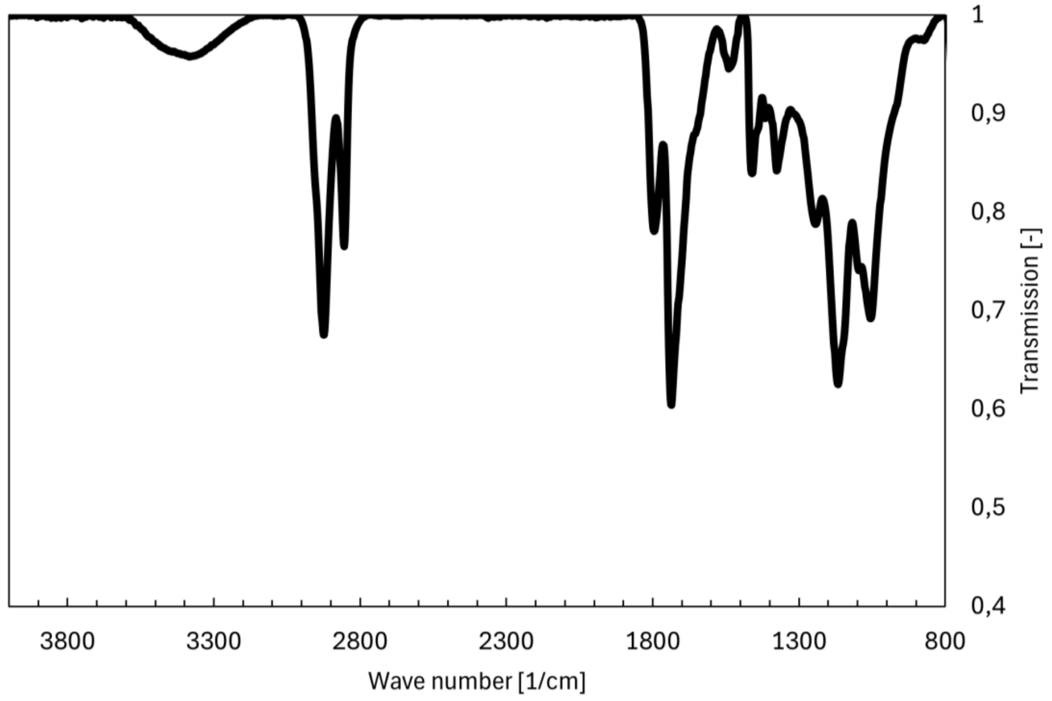

Figure S6. IR-spectrum of NIPU film 1.

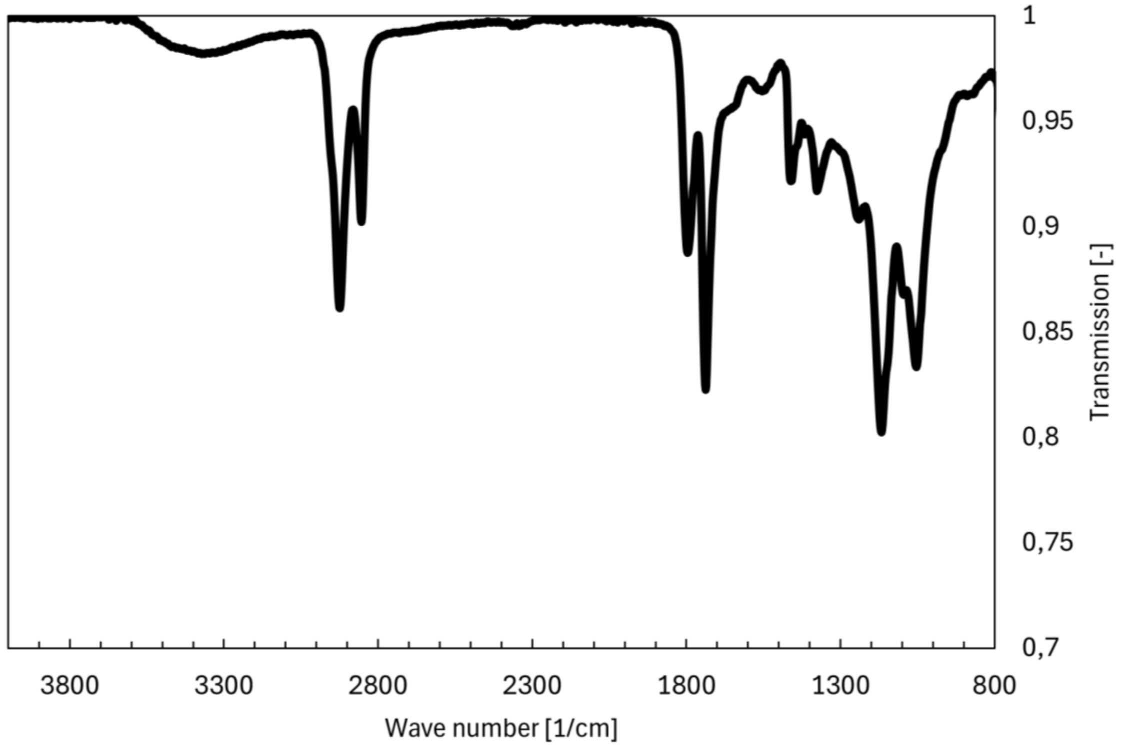

Figure S7. IR-spectrum of NIPU film 2.

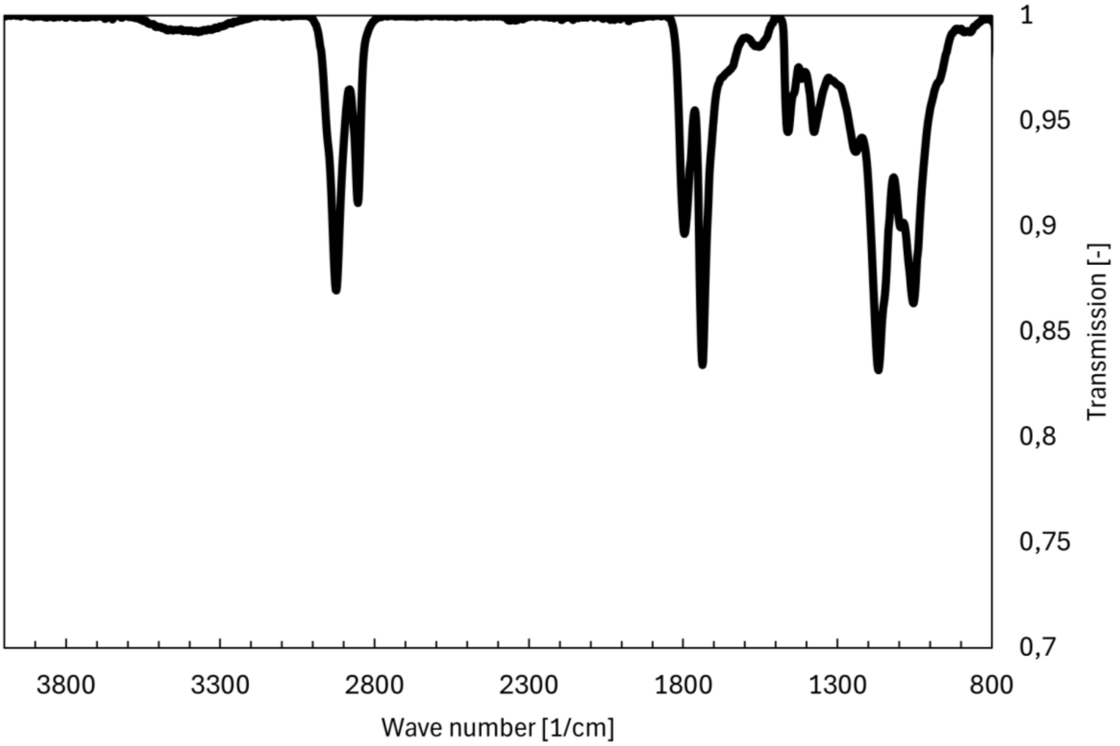

Figure S8. IR-spectrum of NIPU film 3.

Table S2. Observed IR-bands in the monomer and NIPU films and the assigned bond vibrations.

Observed IR-bands in monomer [cm^{-1}]	Observed IR-bands in NIPU film [cm^{-1}]	Assigned bond vibration
3480	3440	OH-stretch
/	3370	NH-stretch (urethane)
2926	2925	CH-stretch
2854	2854	CH-stretch
1793	1793	C=O stretch (cyclic carbonate)
1738	1735	C=O stretch (ester)
/	~1714	C=O stretch (urethane)
/	1530	NH-bend (urethane)
1460	1458	CH-bend
1377	1375	CH-rock
1230	1230	C-O stretch
1170	1166	C-O stretch
1087	1081	C-O stretch
1054	1053	C-O stretch

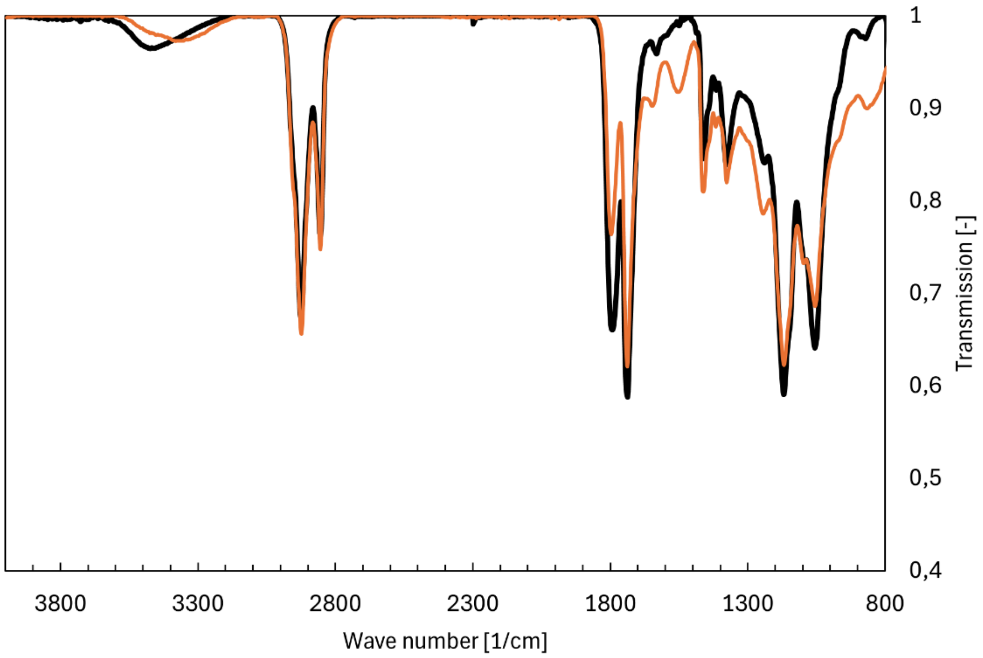

Figure S9. IR-spectra of 8-CC before (black) and after the reaction with hexylamine (orange).

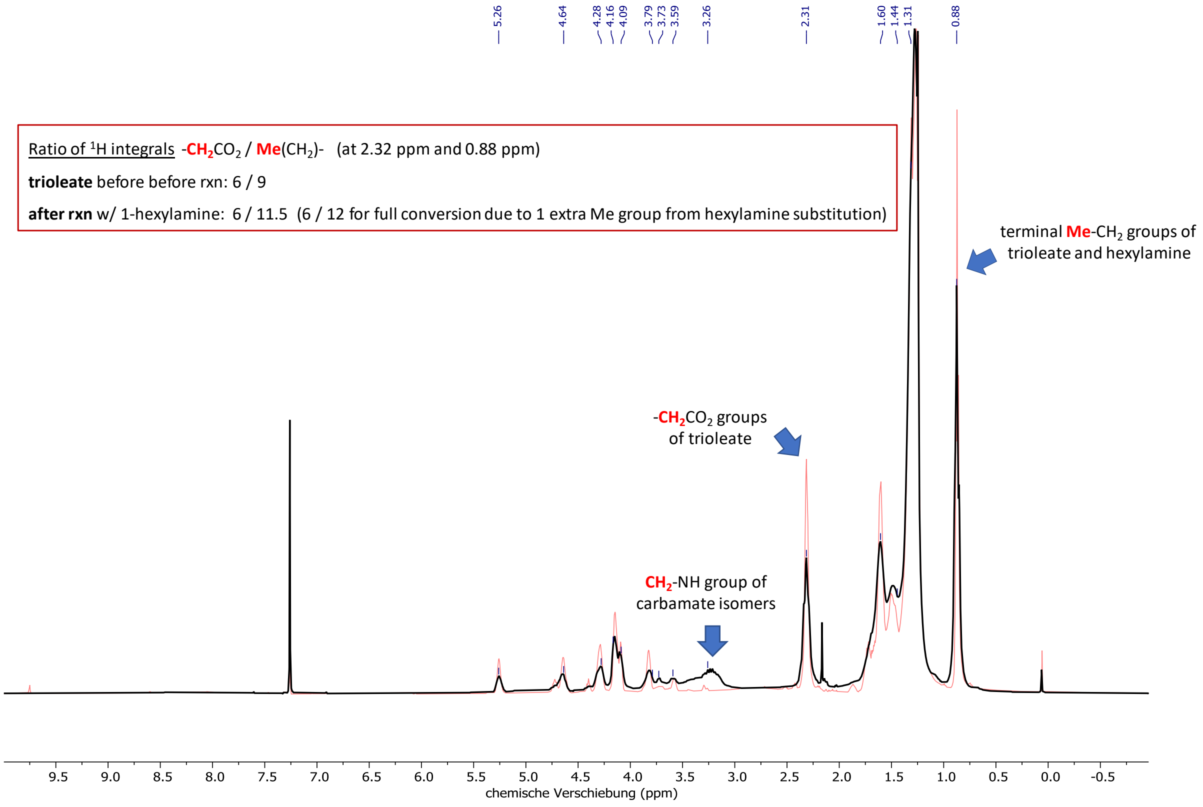

Figure S10. Overlay of the ^{1}H NMR spectra (in $CDCl_3$) of 8-CC (red) and its reaction product with 1-hexylamine (black). The reaction afforded a complex reaction mixture with incomplete conversion and

the formation of various regioisomers and stereoisomers. From the integration of the ^{1}H resonances at 2.32 ppm ($\underline{CH_2}$-C(=O)-O-) and 0.88 pm ($\underline{Me}$-CH_2-), a conversion of approx. 40% was determined.

7. References

[31] P. Bayer, A. Jacobi von Wangelin, *Green Chem.* **2020**, *22*, 2359–2364

[48] J. Schachtner, P. Bayer, A. Jacobi von Wangelin, *Beilstein J. Org. Chem.* **2016**, *12*, 1798–1811

[65] Y. Fu, Y. Weng, W. X. Hong, Q. Zhang, *Synlett* **2011**, *6*, 809–812.

[66] A. Das, N. Chatani, *Chem. Sci.* **2021**, *12*, 3202–3209.

[67] H. Sharghi, A. Hassani Nejad, *Tetrahedron* **2004**, *60*, 1863–1868.

[68] N. A. Porter, K. A. Mills, S. E. Caldwell, G. R. Dubay, *J. Am. Chem. Soc.* **1994**, *116*, 6697–6705.

[69] N. A. Porter, J. S. Wujek, *J. Org. Chem*. **1987**, *52*, 5085–5089.

8. Experimental Spectra

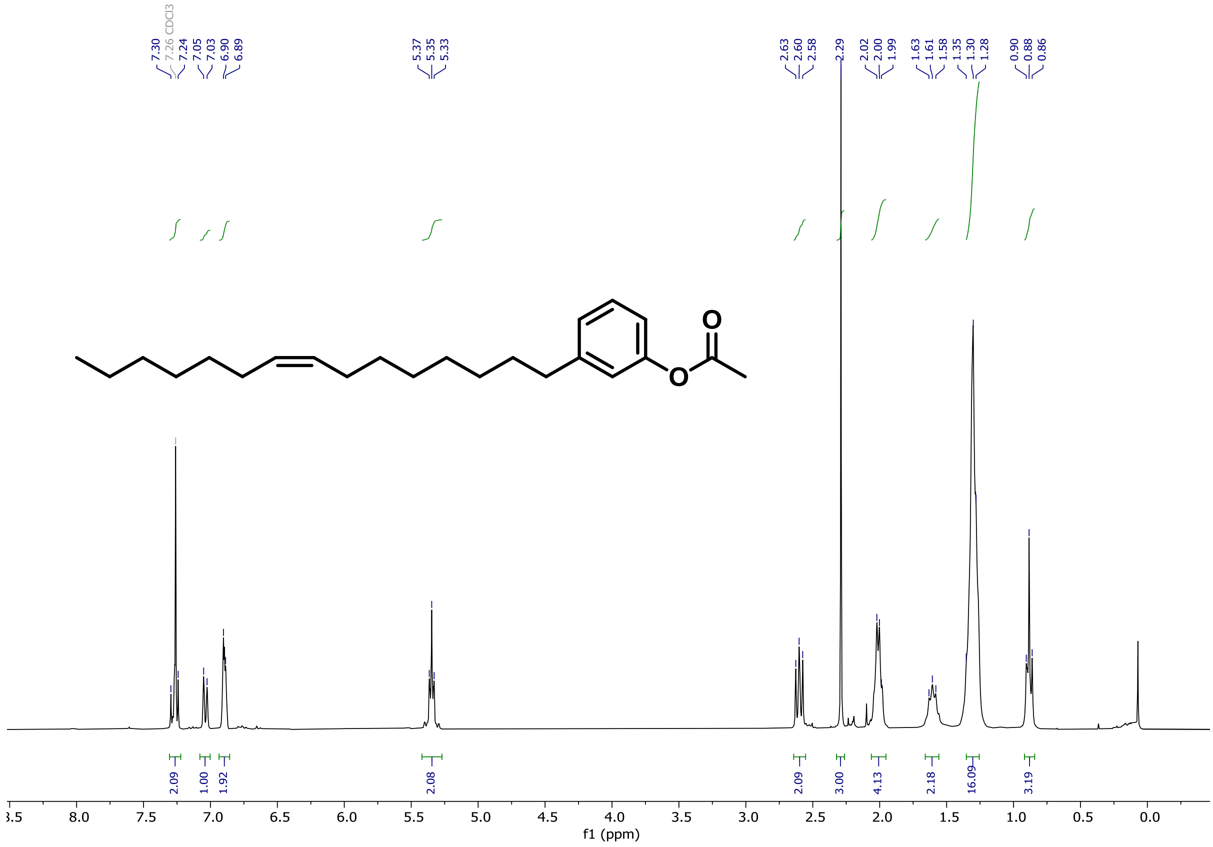

^1H NMR (300 MHz, $CDCl_3$) of cardanyl acetate (CA).

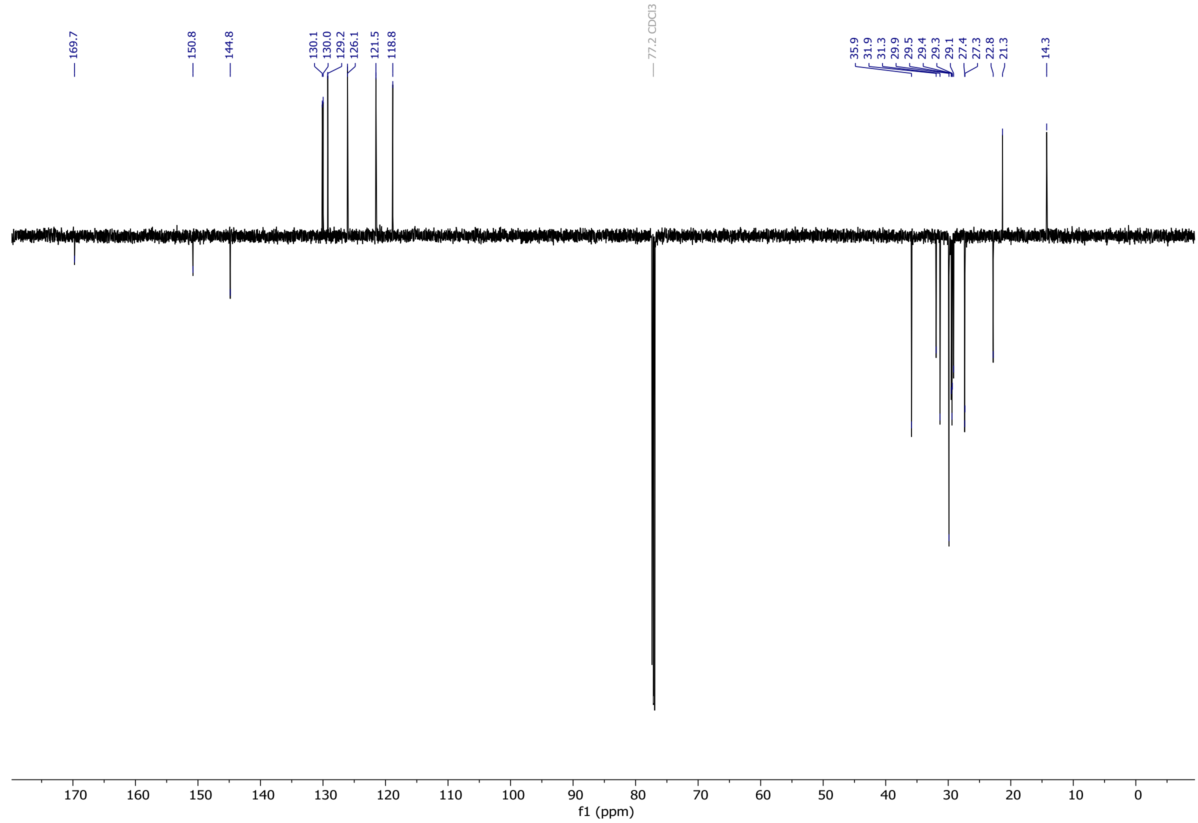

^{13}C NMR (151 MHz, $CDCl_3$) of cardanyl acetate (CA).

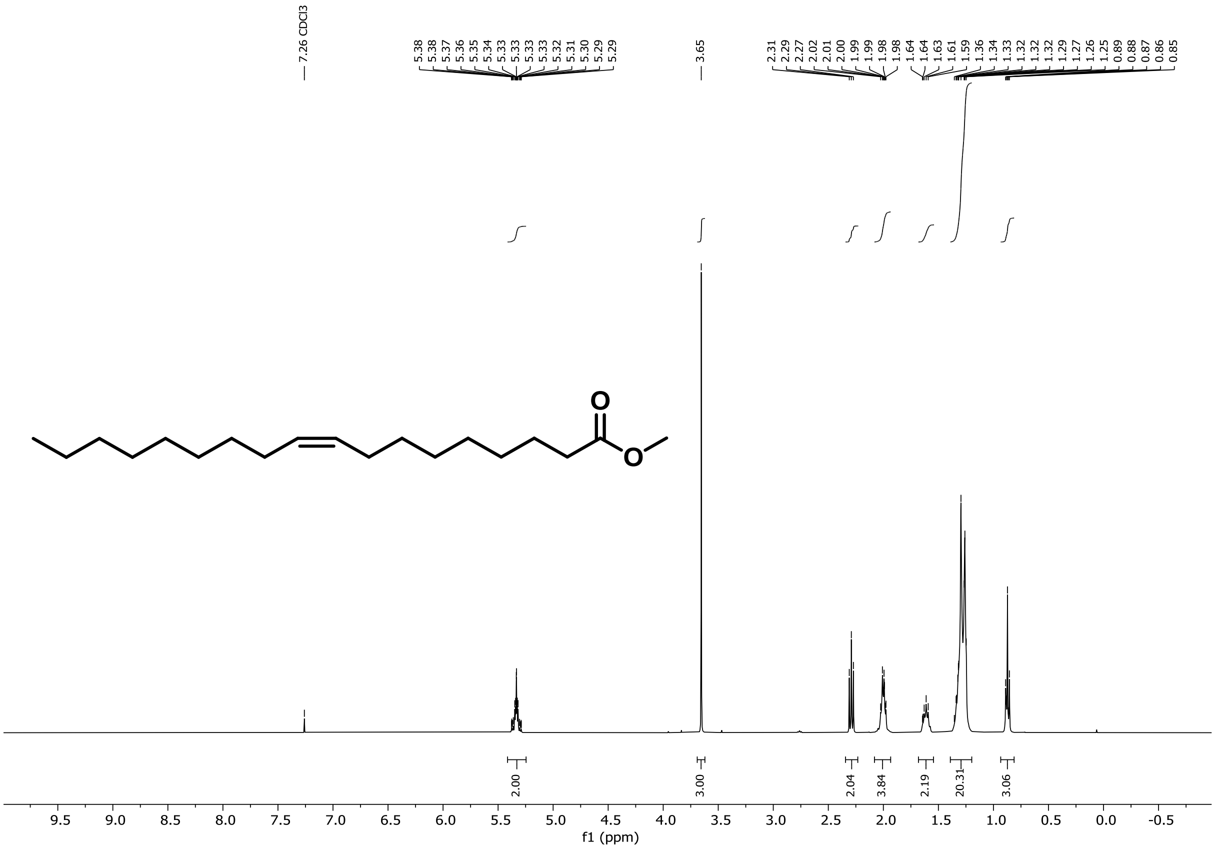

^{1}H NMR (400 MHz, $CDCl_3$) of methyl oleate (MO).

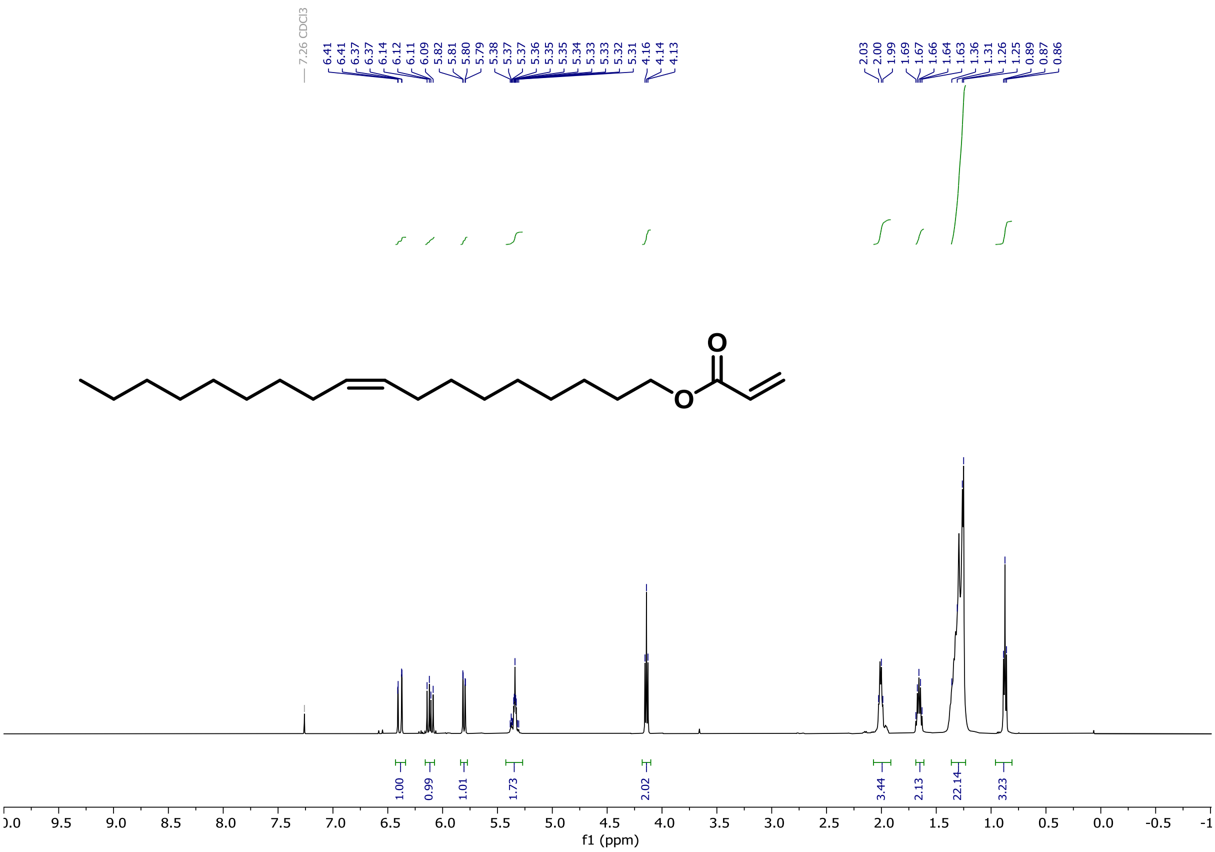

^{1}H NMR (500 MHz, $CDCl_3$) of oleyl acrylate (OA).

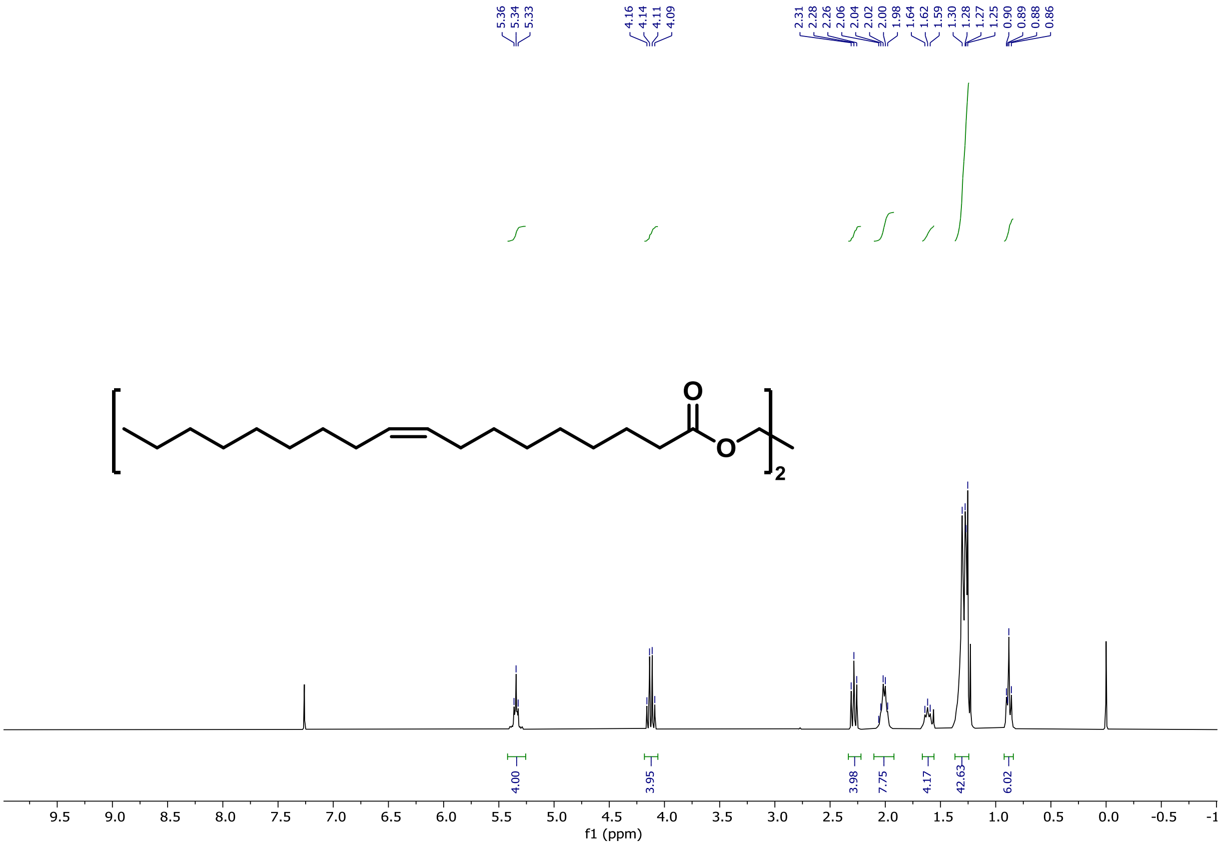

^{1}H NMR (300 MHz, $CDCl_3$) of trimethylene glycol dioleate (DO).

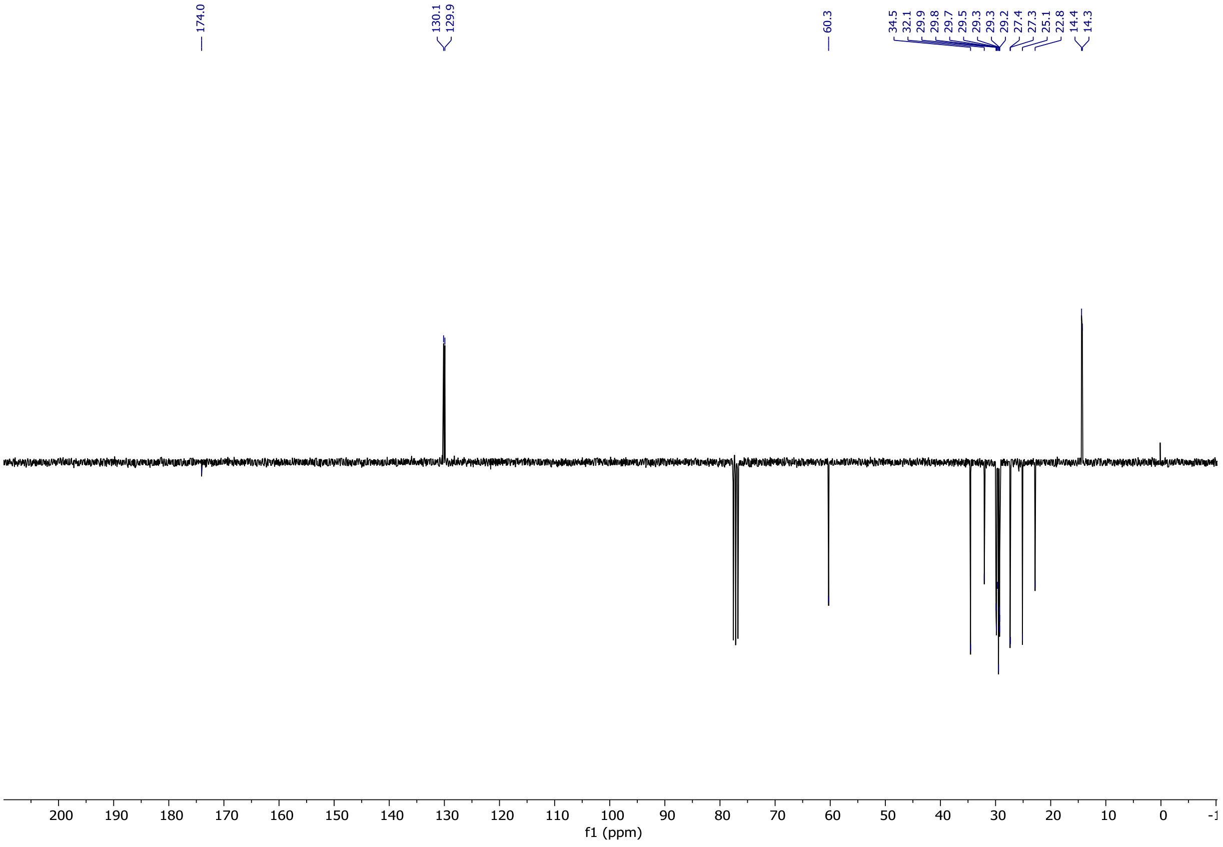

^{13}C NMR (75 MHz, $CDCl_3$) of trimethylene glycol dioleate (DO).

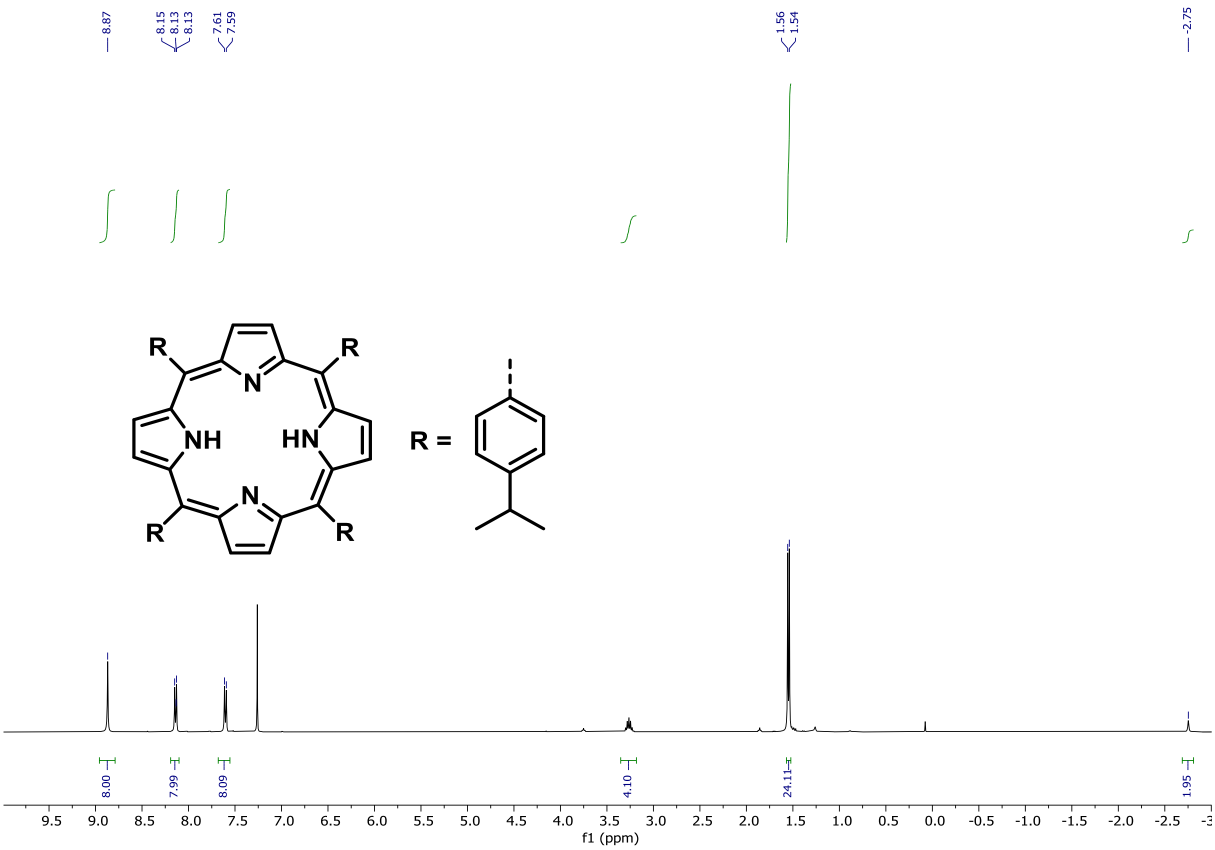

^{1}H NMR (400 MHz, $CDCl_3$) of iPrTPP.

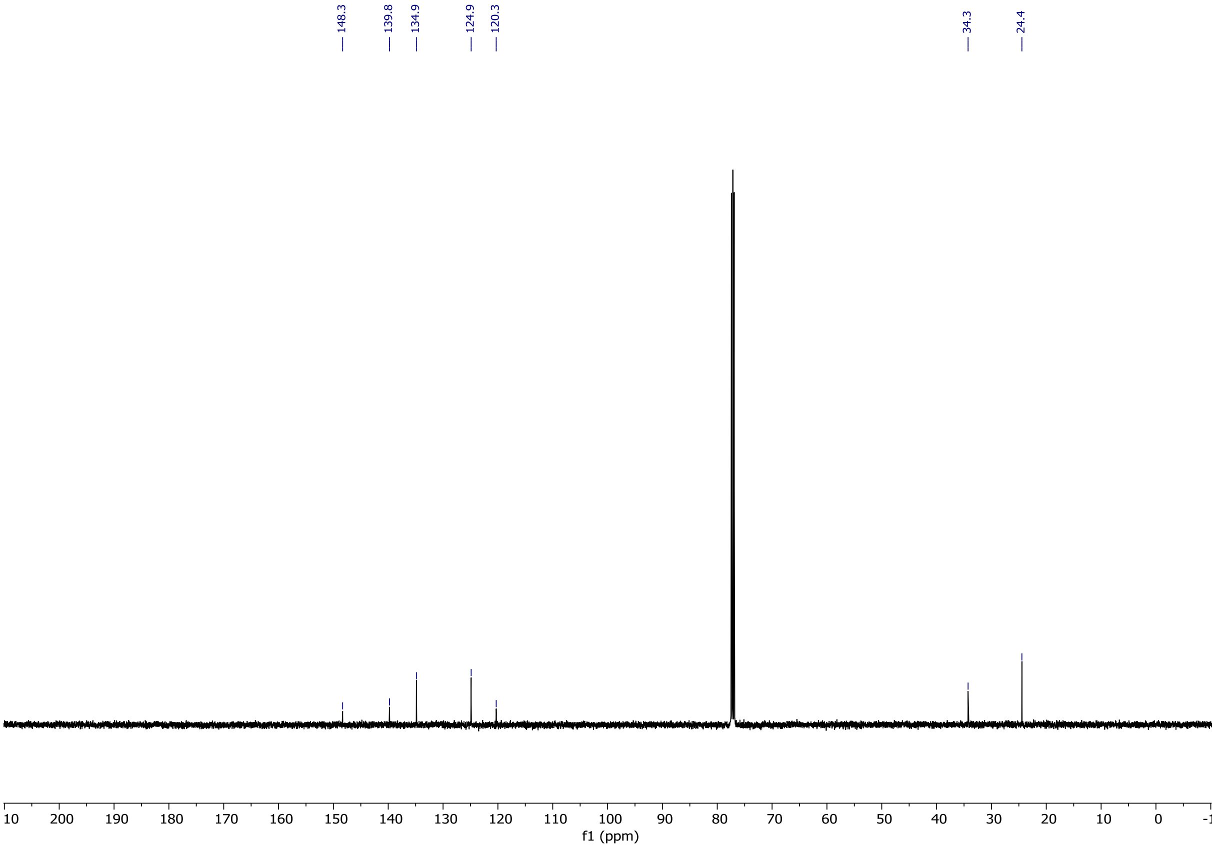

^{13}C NMR (126 MHz, $CDCl_3$) of iPrTPP.

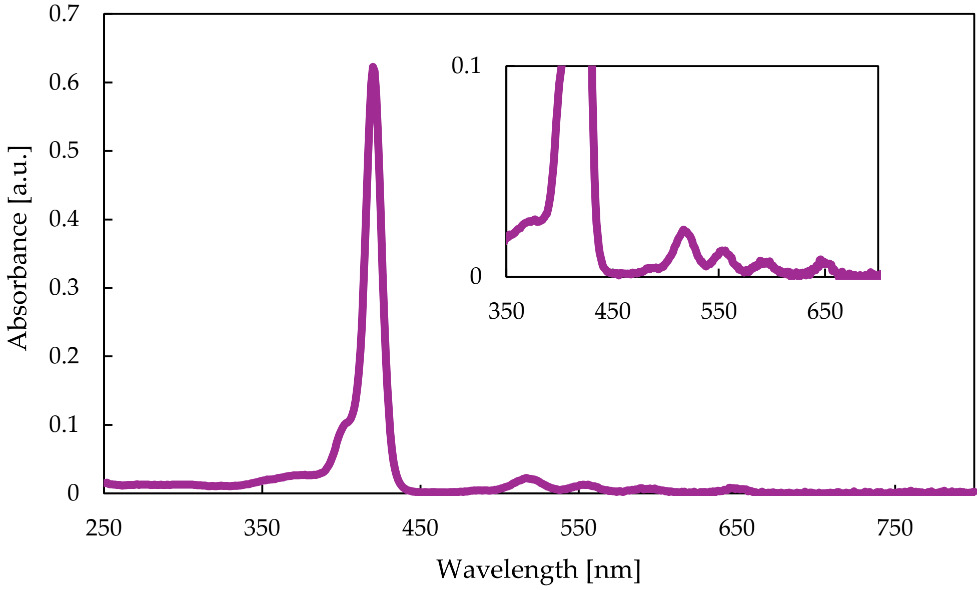

UV/Vis spectrum of iPrTPP in $CHCl_3$.

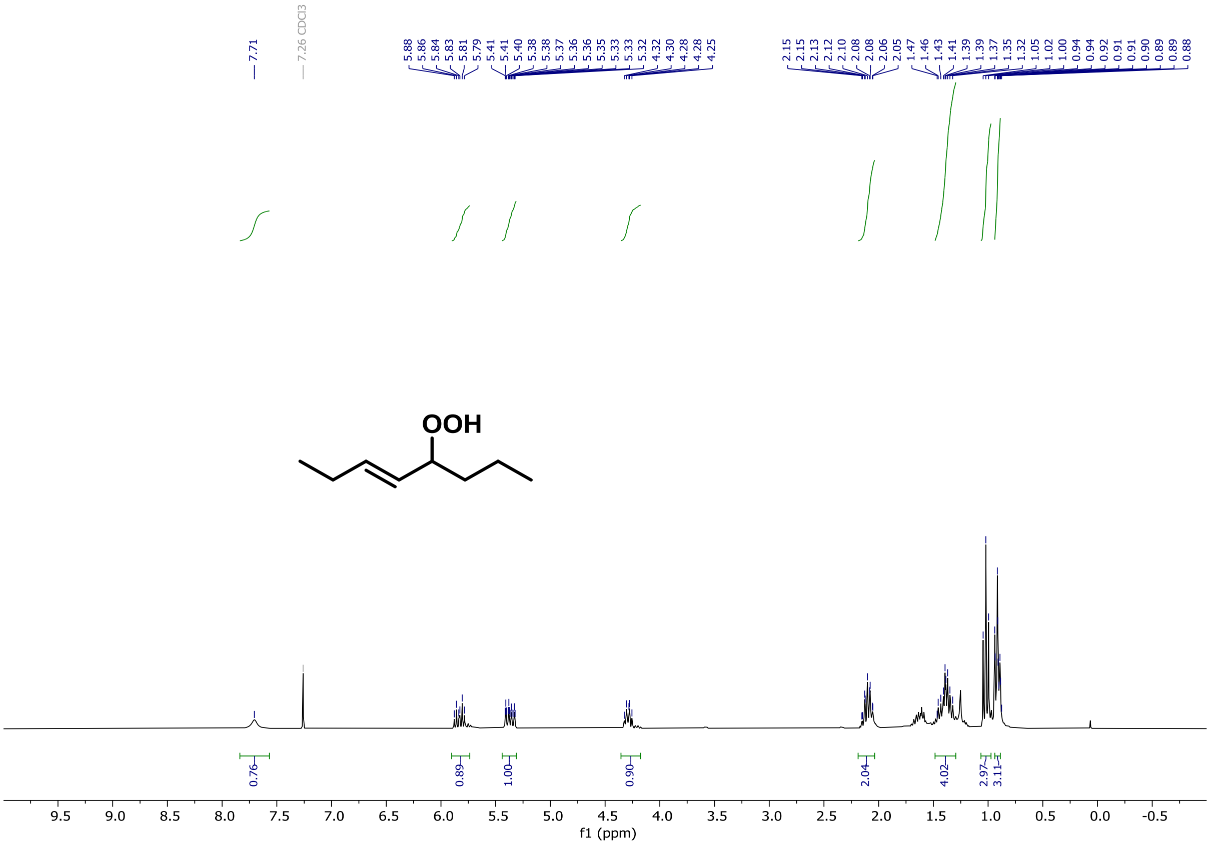

^{1}H NMR (300 MHz, $CDCl_3$) of 1-HYP.

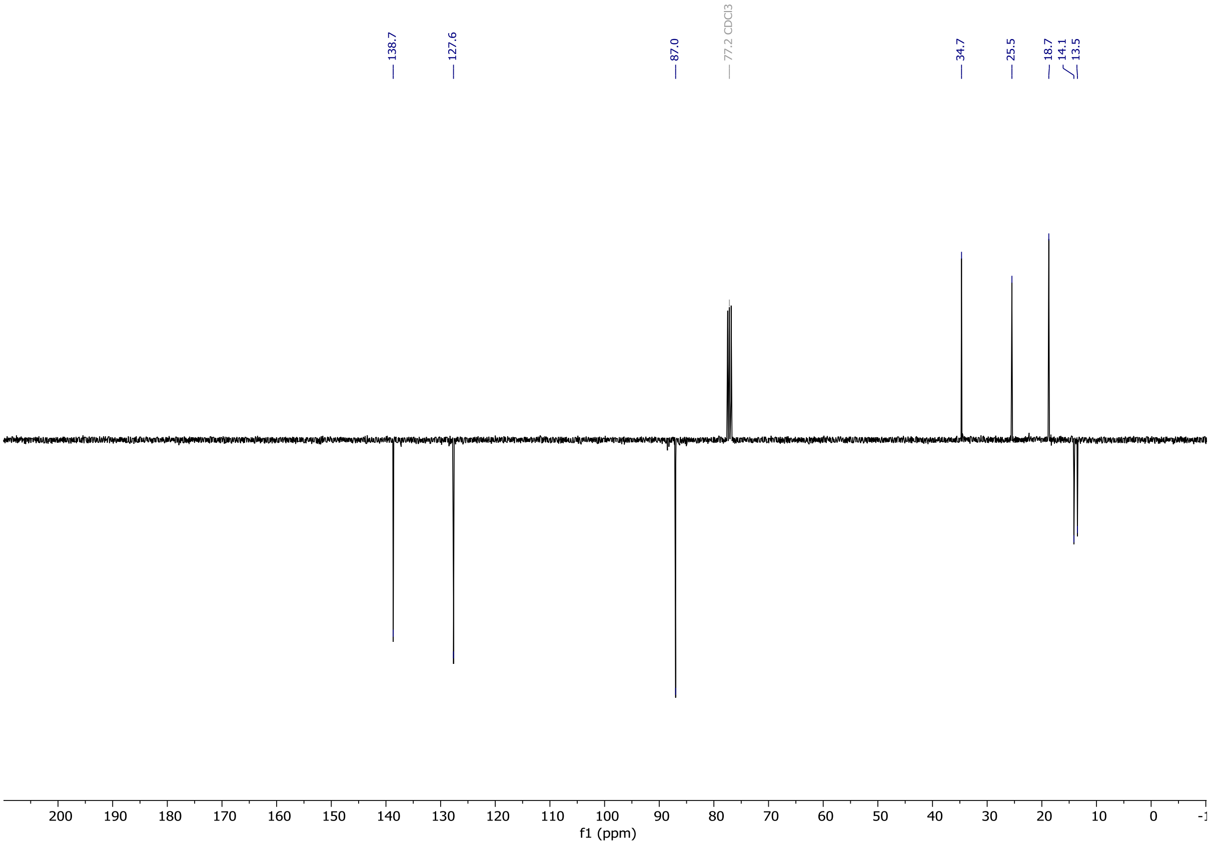

^{13}C NMR (100 MHz, $CDCl_3$) of 1-HYP.

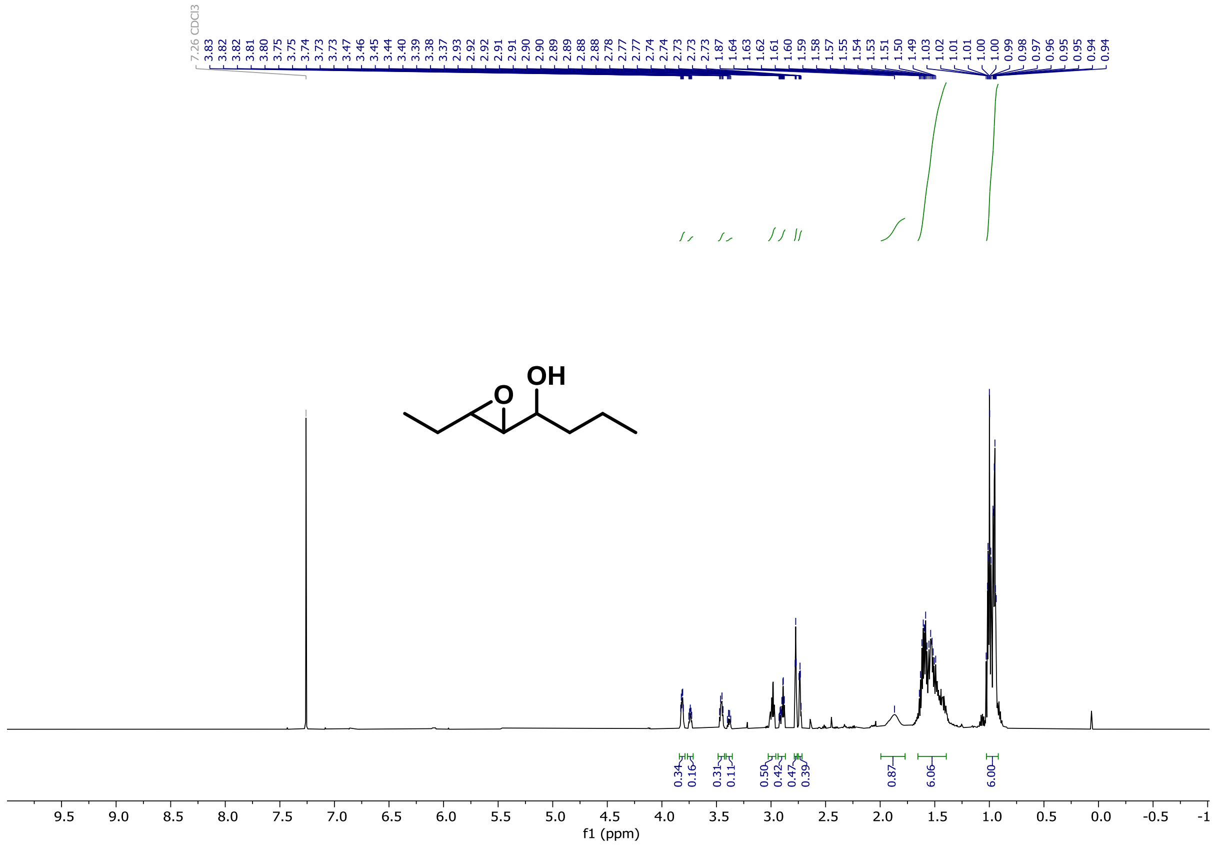

^{1}H NMR (300 MHz, $CDCl_3$) of 1-EpAlc.

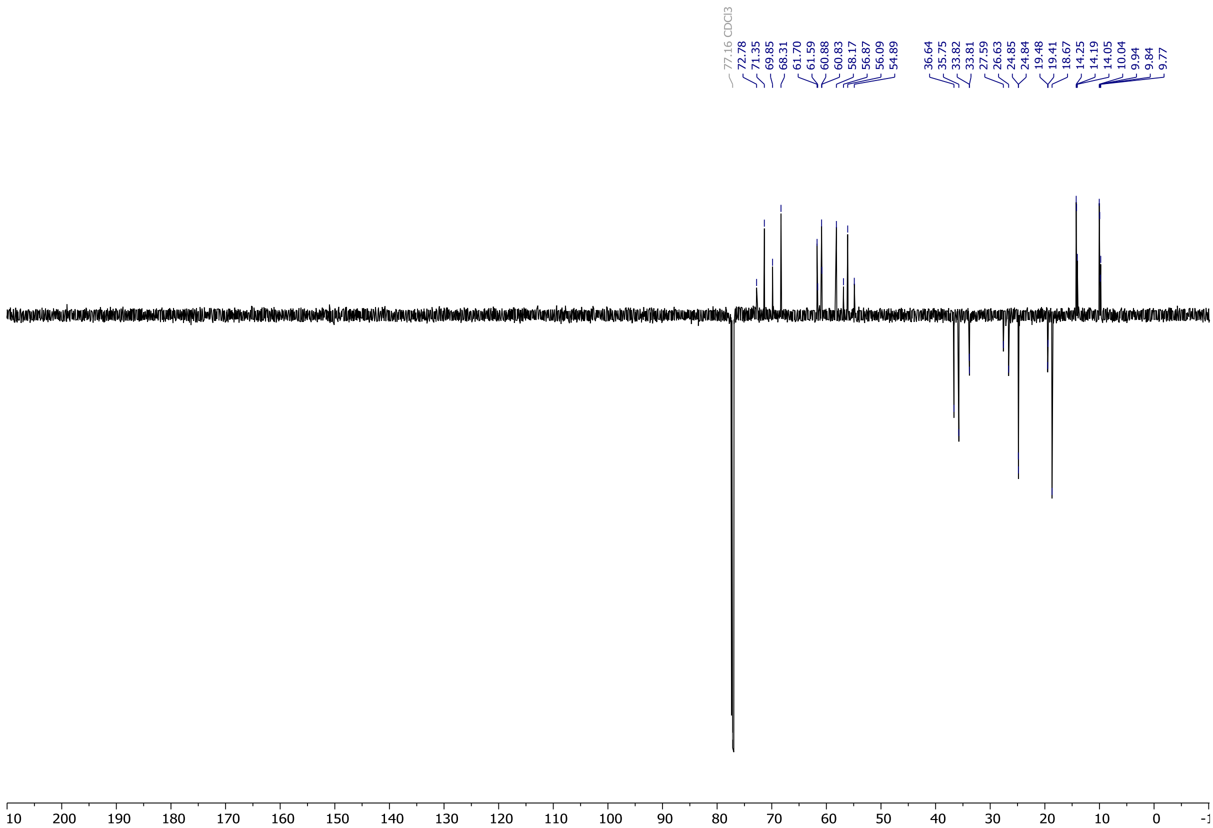

^{13}C NMR (151 MHz, $CDCl_3$) of 1-EpAlc.

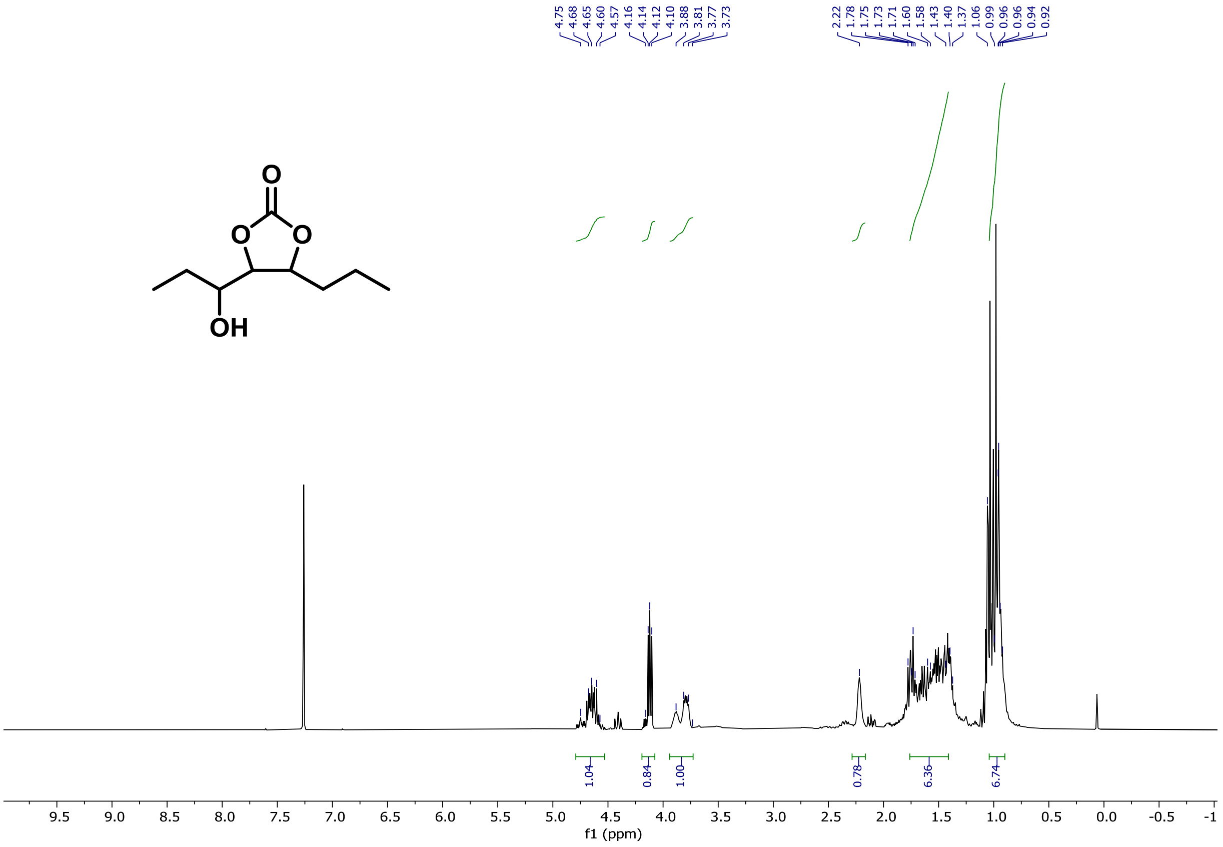

¹H NMR (300 MHz, $CDCl_3$) of 1-CC.

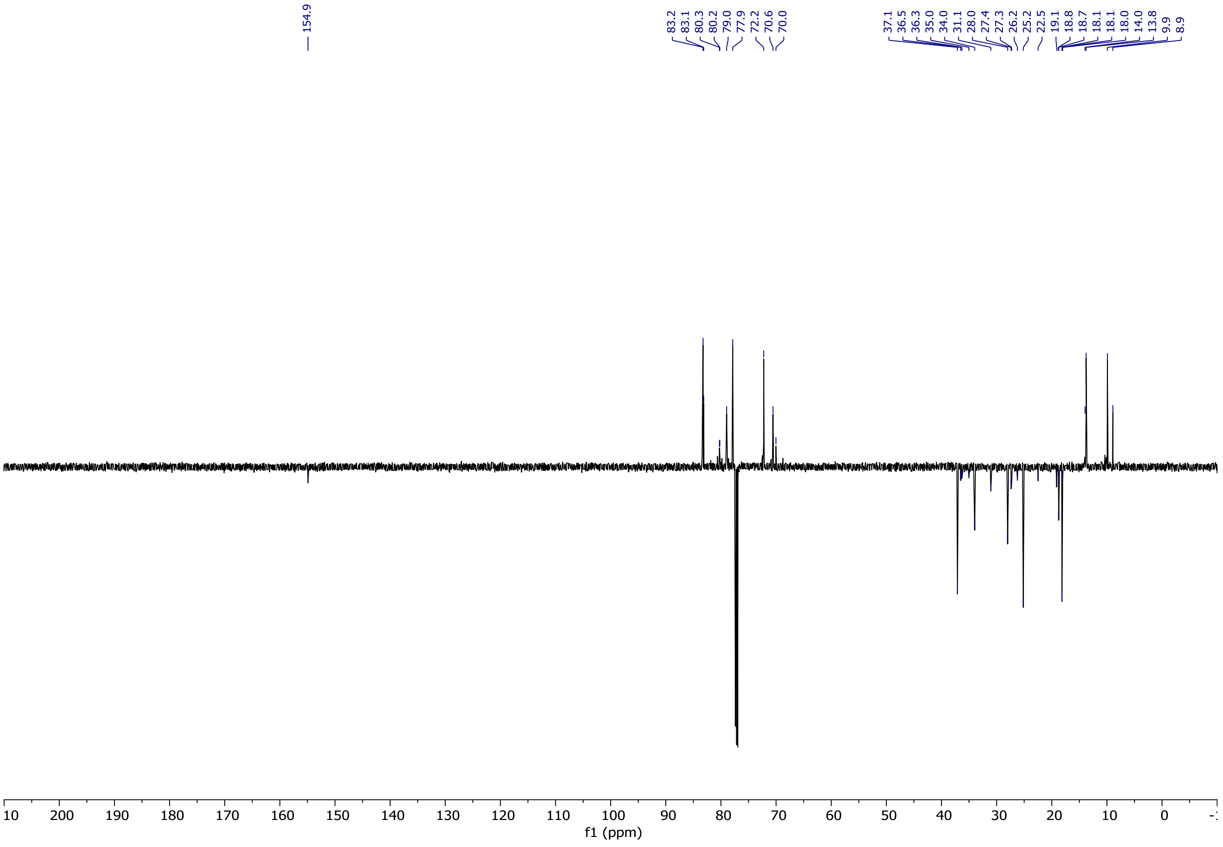

^{13}C NMR (151 MHz, $CDCl_3$) of 1-CC.

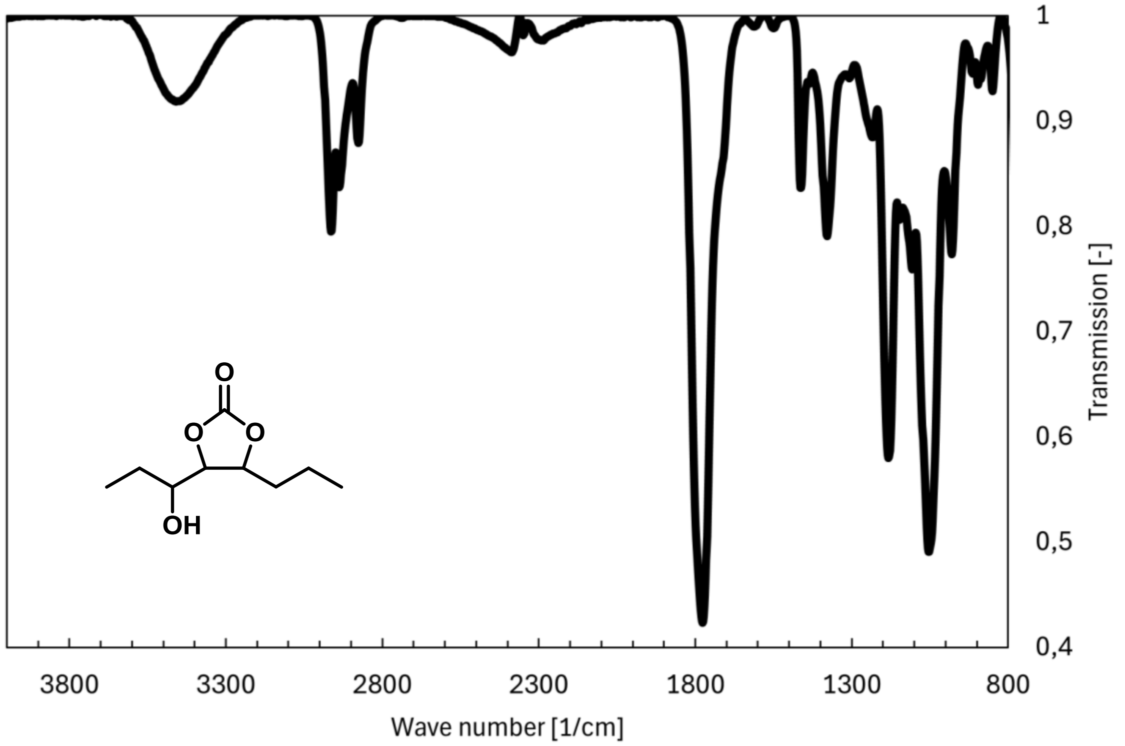

IR spectrum of 1-CC.

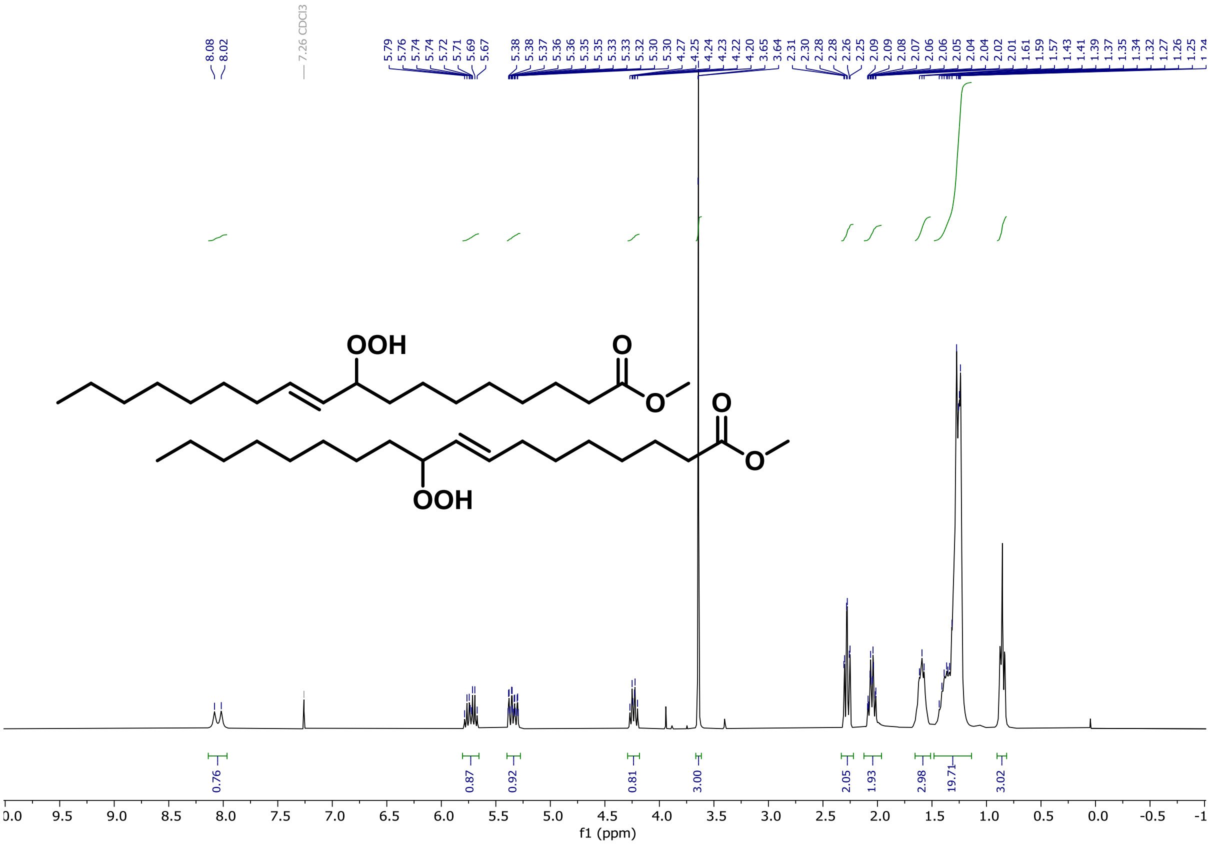

¹H NMR (300 MHz, $CDCl_3$) of 2-HYP.

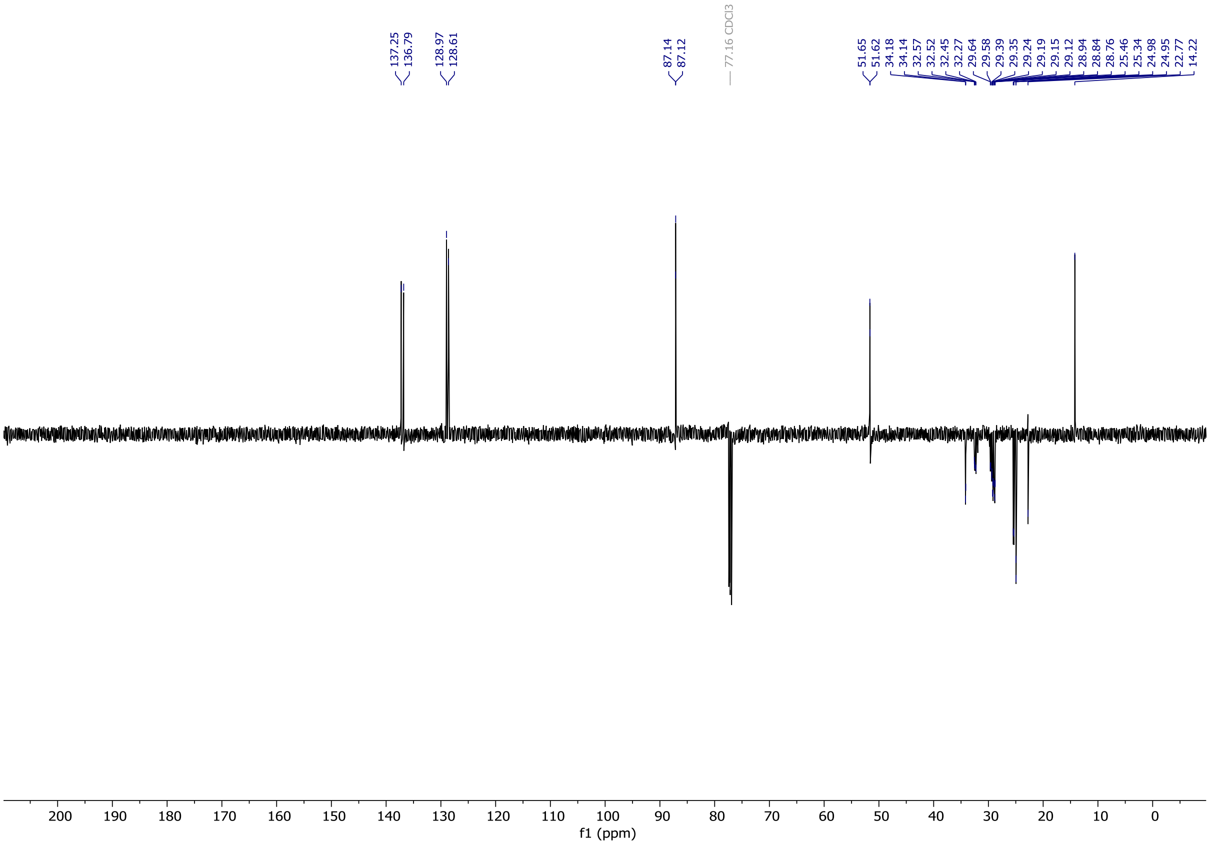

¹³C NMR (126 MHz, $CDCl_3$) of 2-HYP.

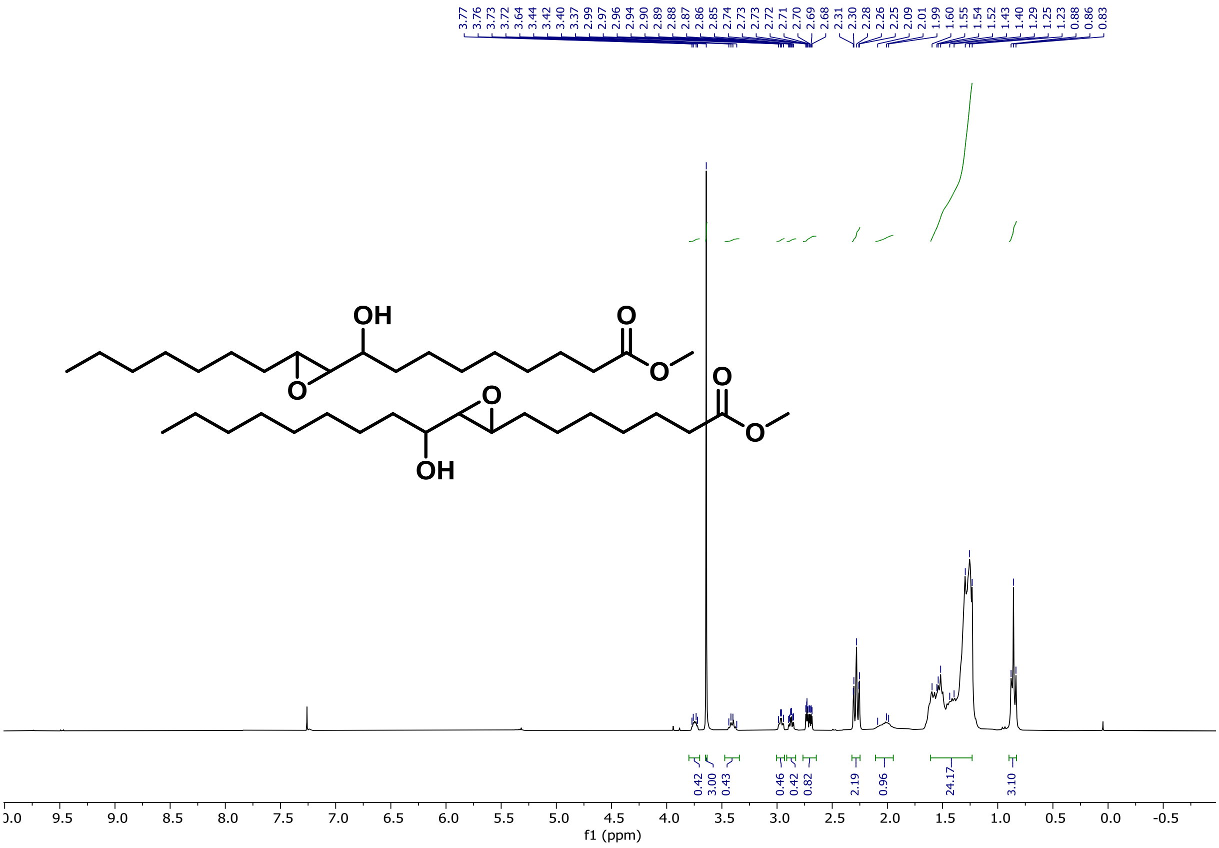

¹H NMR (300 MHz, $CDCl_3$) of 2-EpAlc.

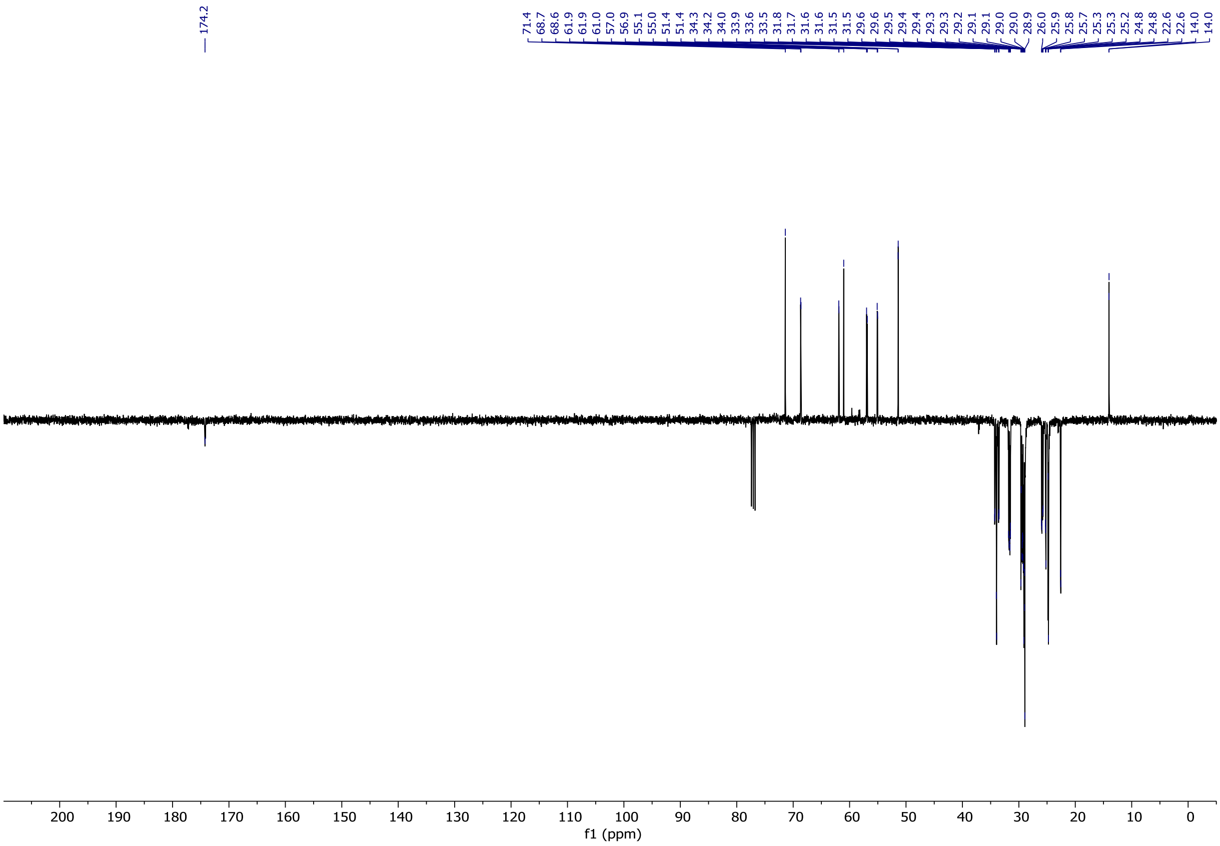

13C NMR (126 MHz, CDCl3) of 2-EpAlc.

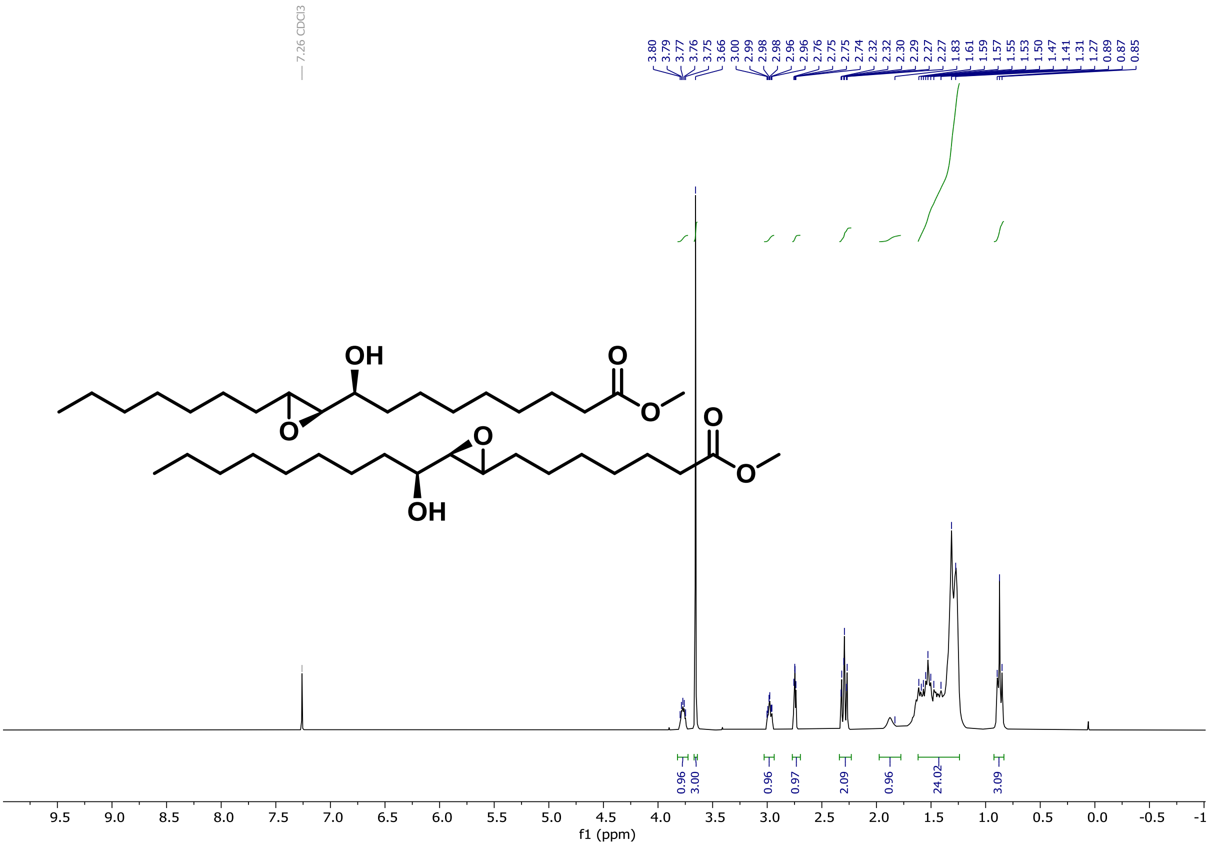

1H NMR (300 MHz, CDCl3) of 2-*syn*-EpAlc.

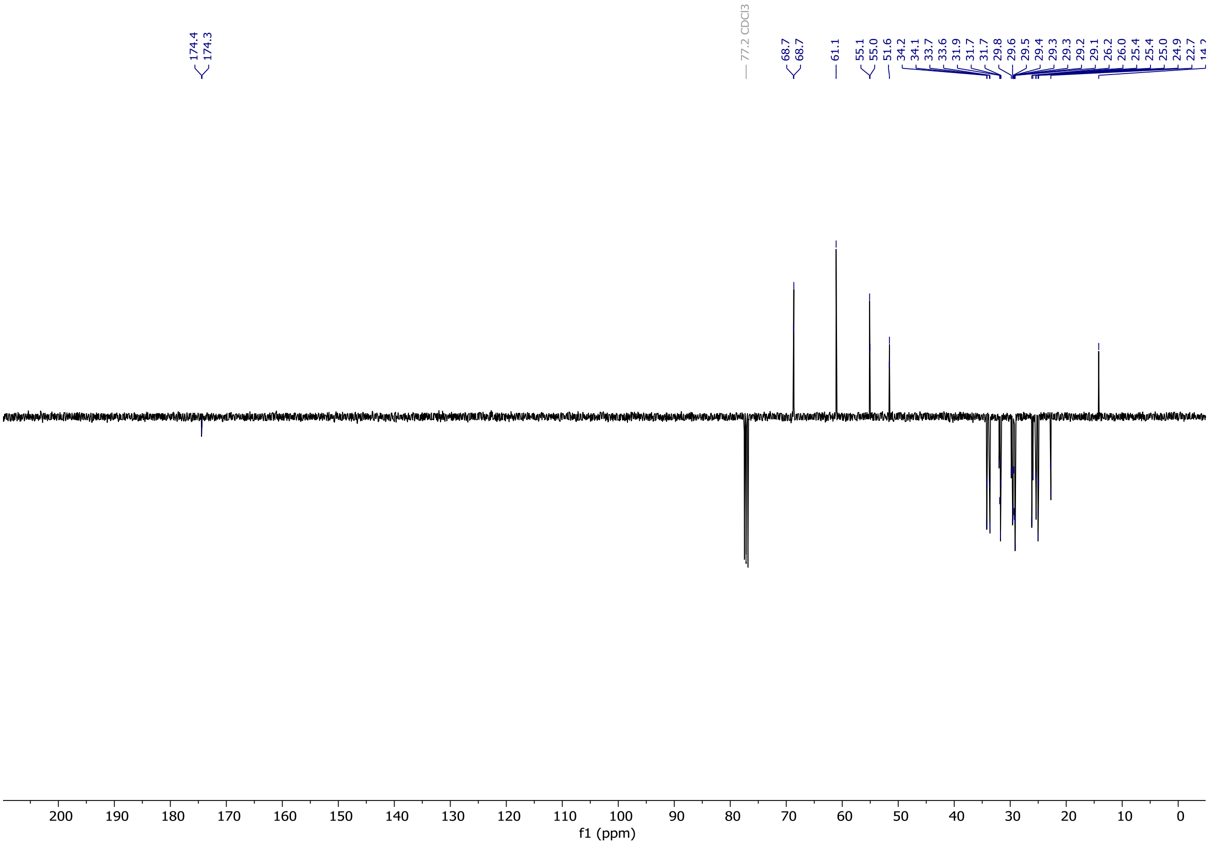

^{13}C NMR (101 MHz, $CDCl_3$) of 2-*syn*-EpAlc.

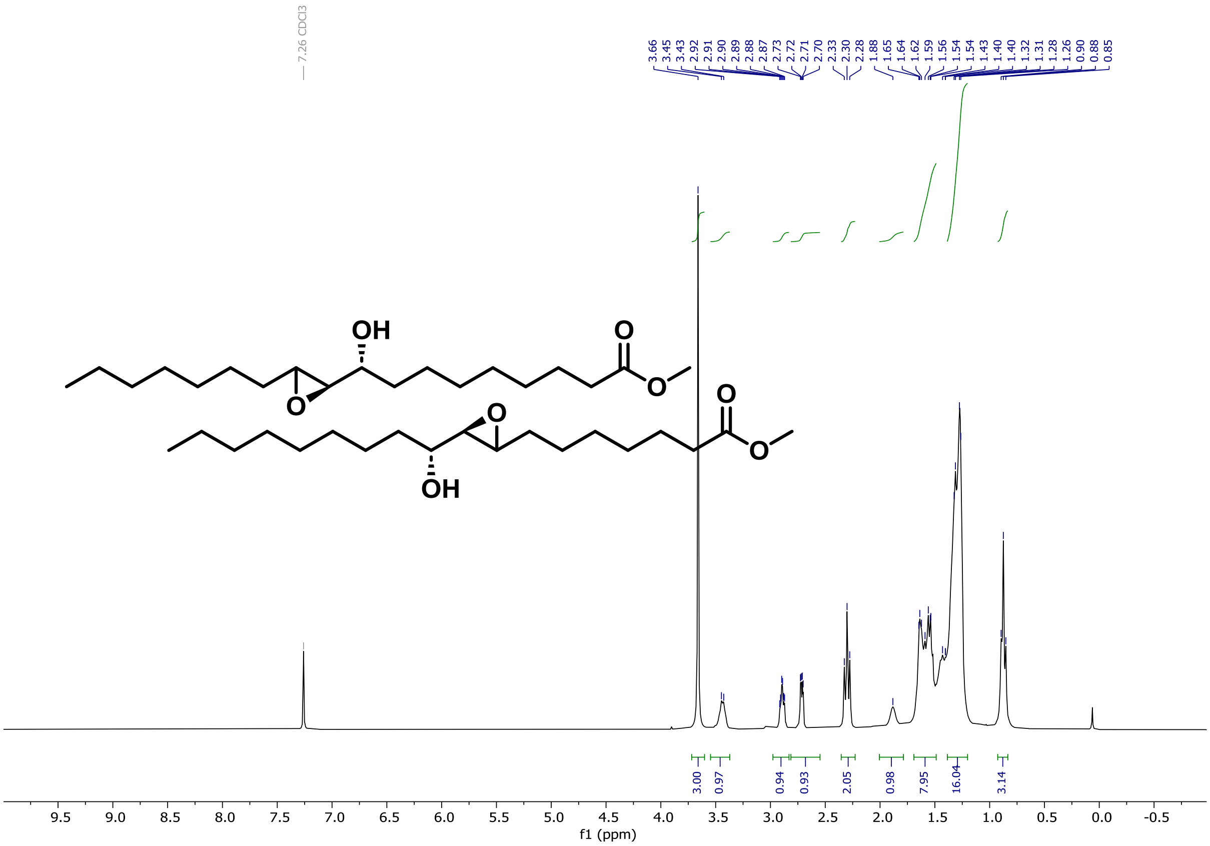

^{1}H NMR (300 MHz, $CDCl_3$) of 2-*anti*-EpAlc.

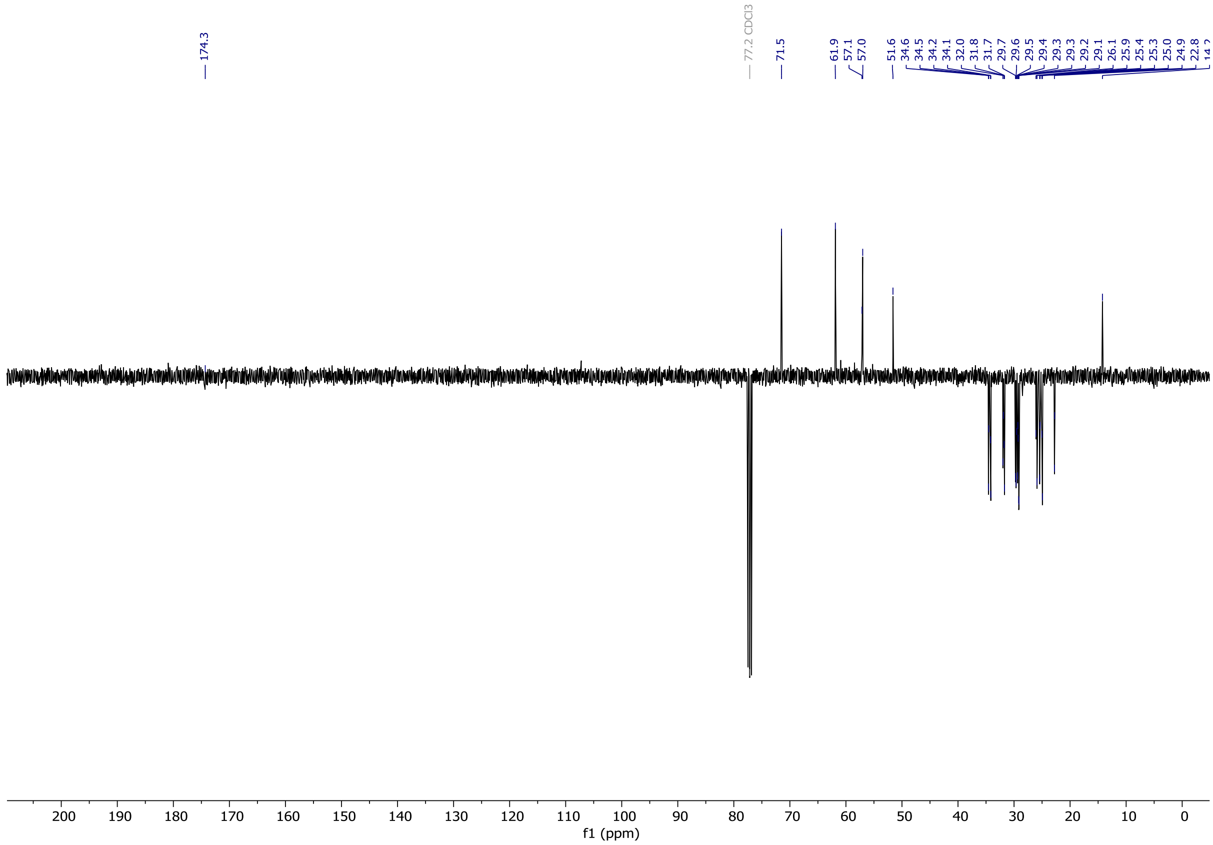

^{13}C NMR (101 MHz, $CDCl_3$) of 2-*anti*-EpAlc.

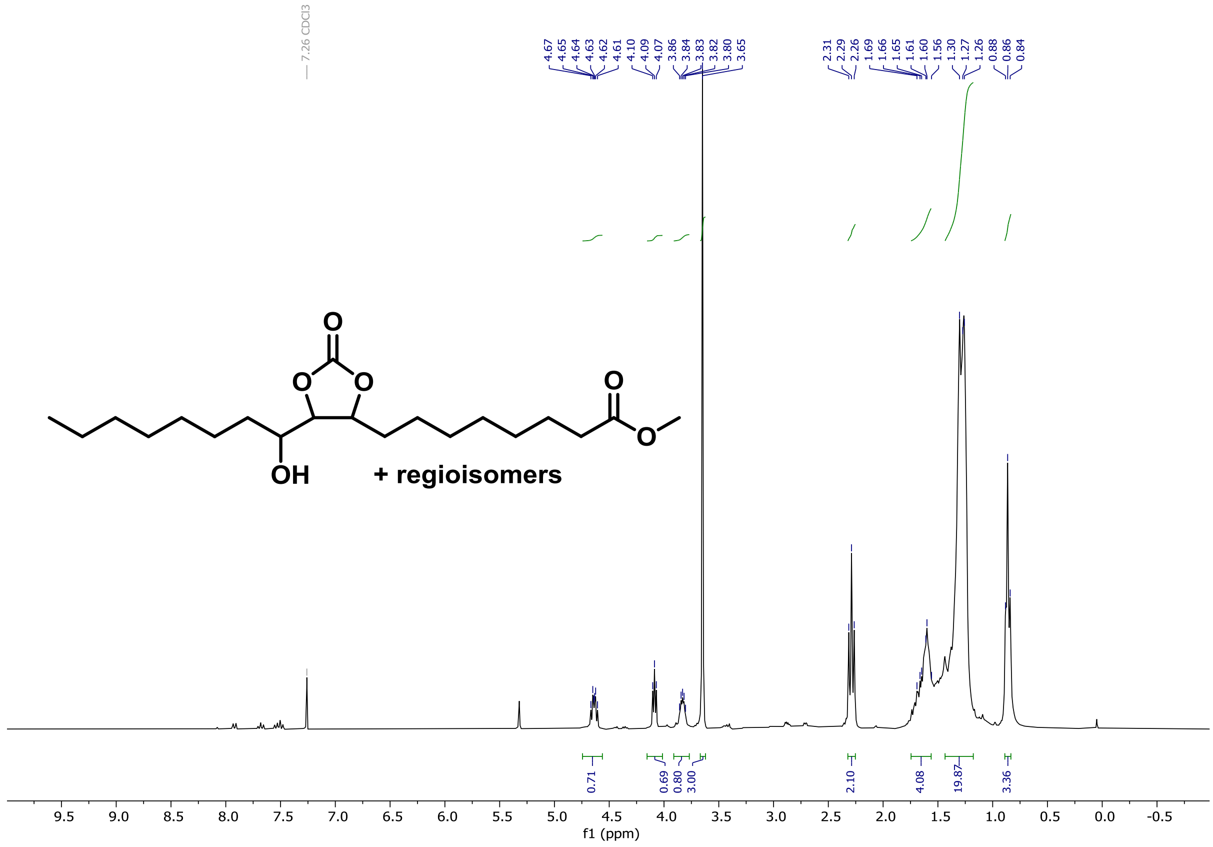

^{1}H NMR (300 MHz, $CDCl_3$) of 2-CC.

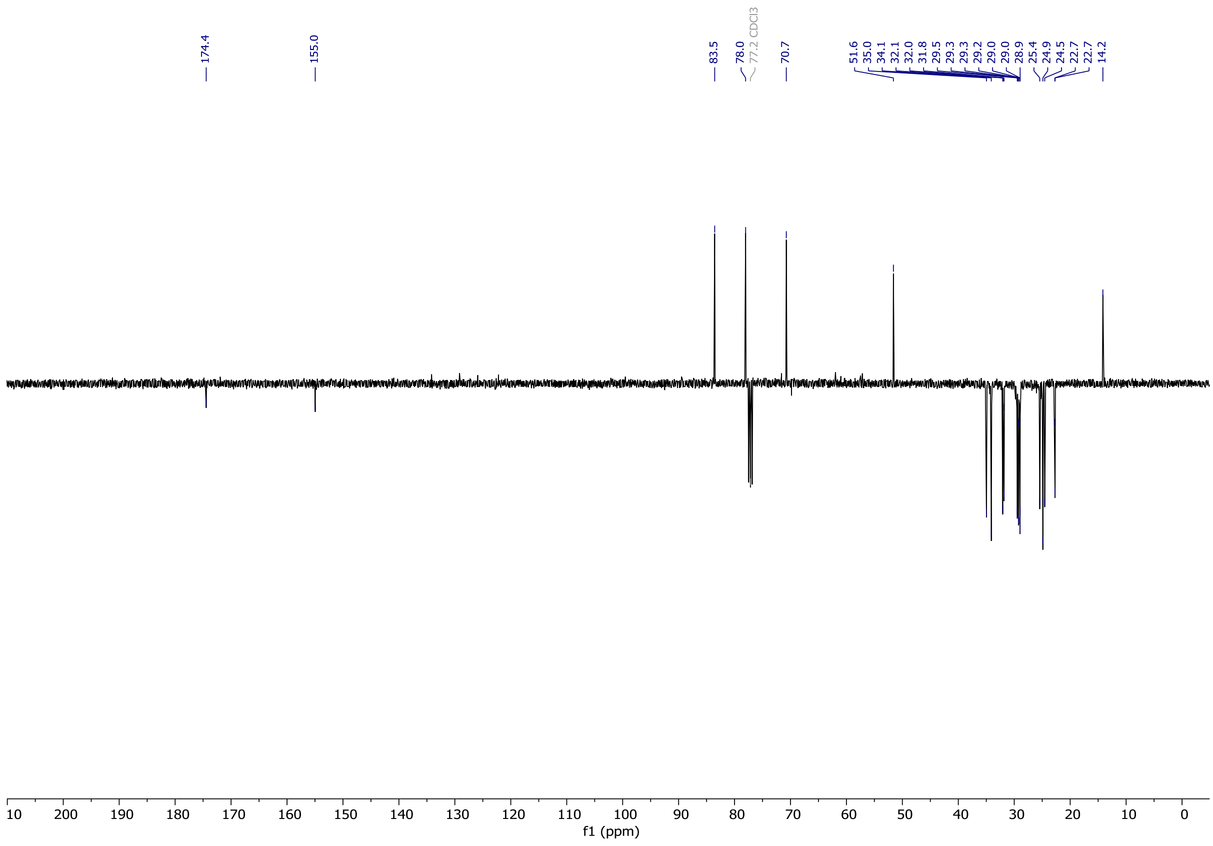

^{13}C NMR (101 MHz, $CDCl_3$) of 2-CC.

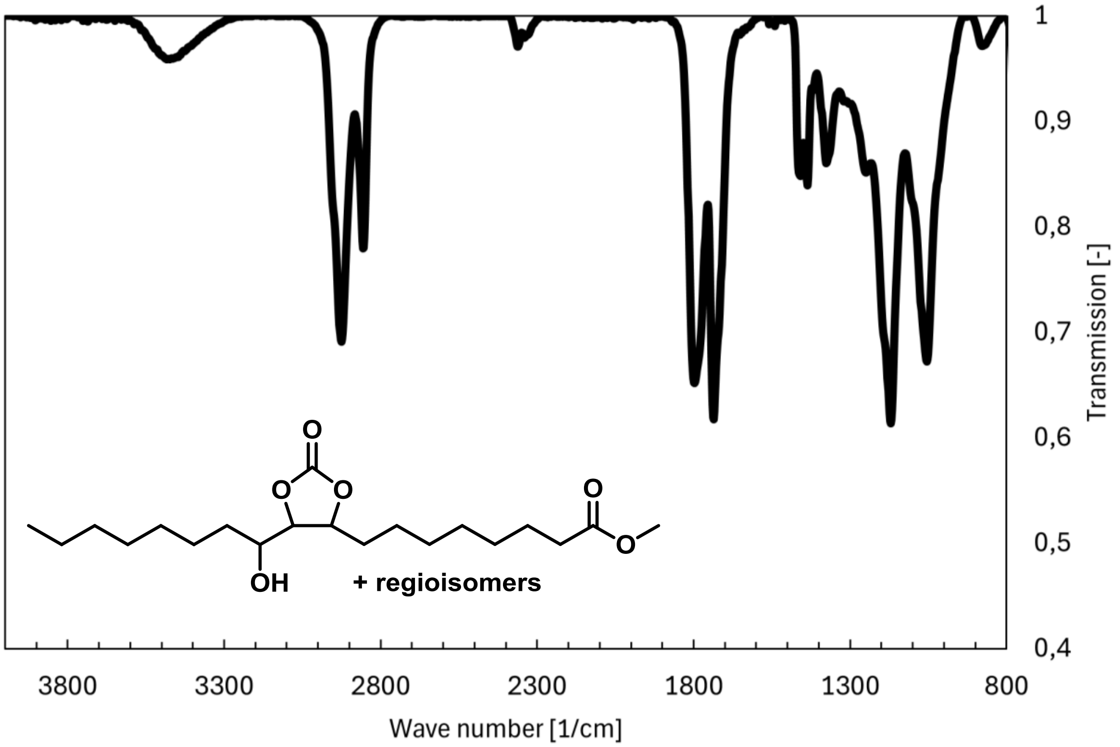

IR spectrum of 2-CC.

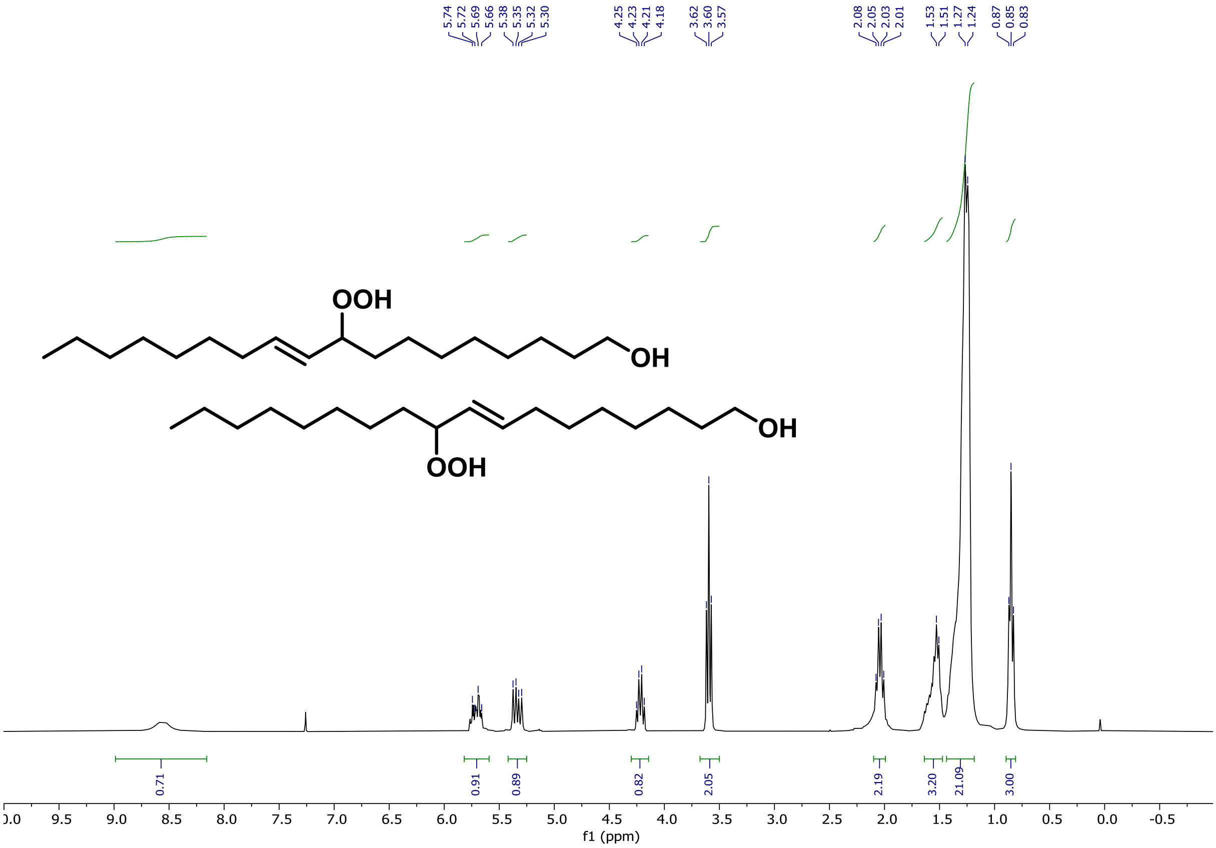

^{1}H NMR (300 MHz, $CDCl_3$) of 3-HYP.

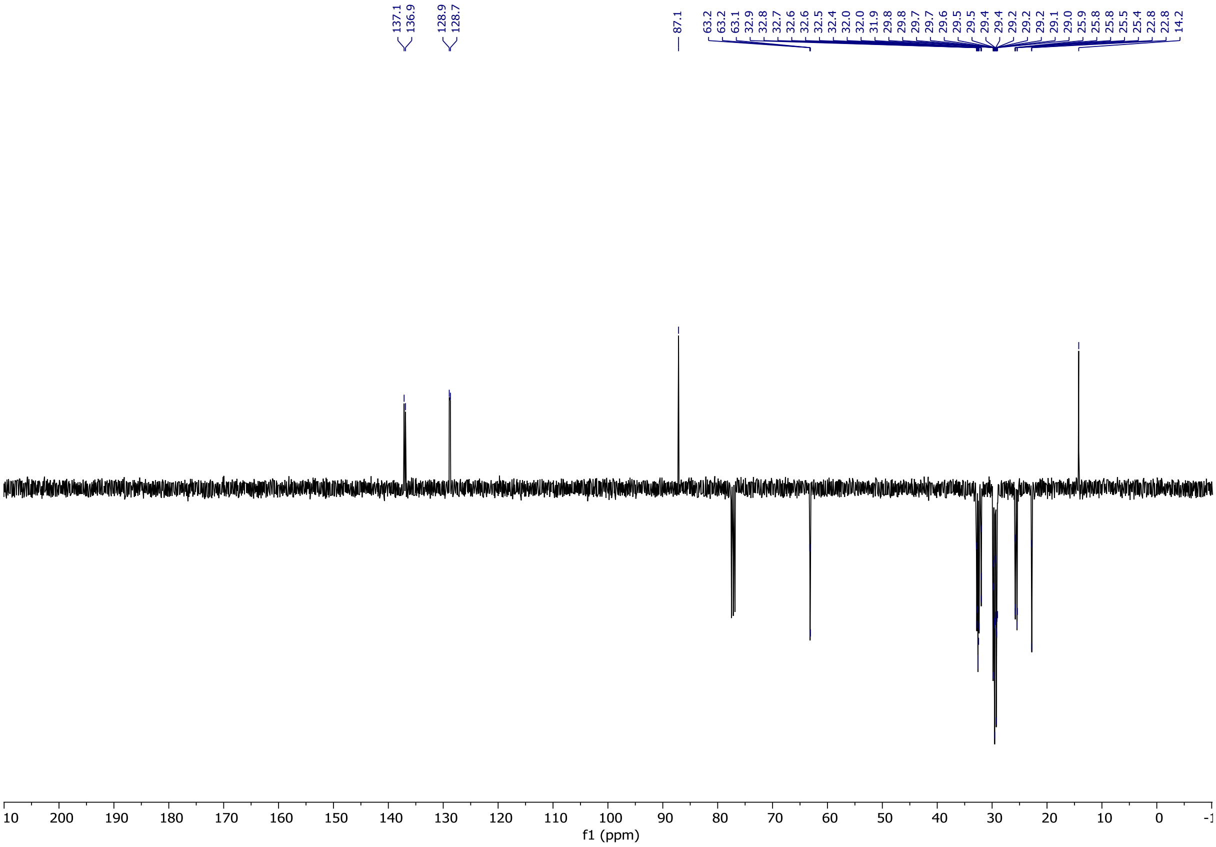

^{13}C NMR (101 MHz, $CDCl_3$) of 3-HYP.

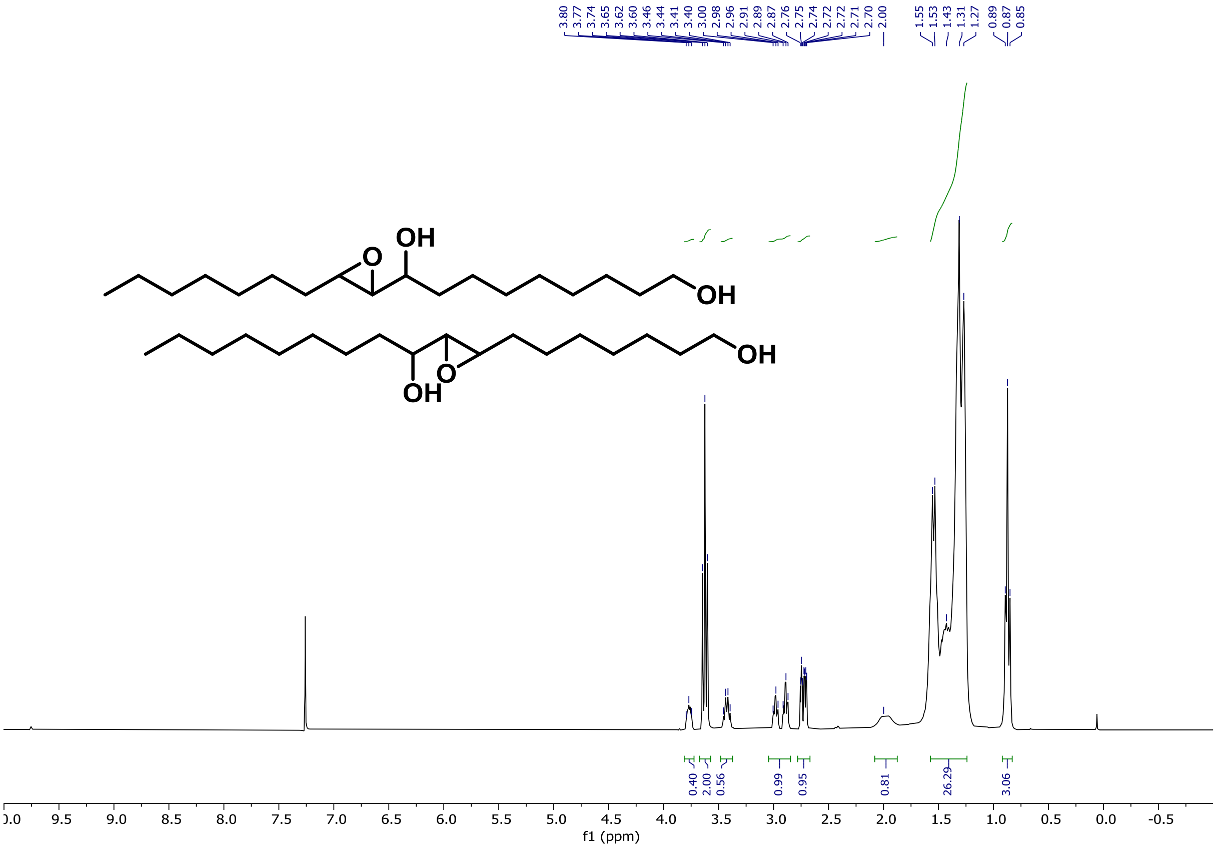

¹H NMR (300 MHz, $CDCl_3$) of 3-EpAlc.

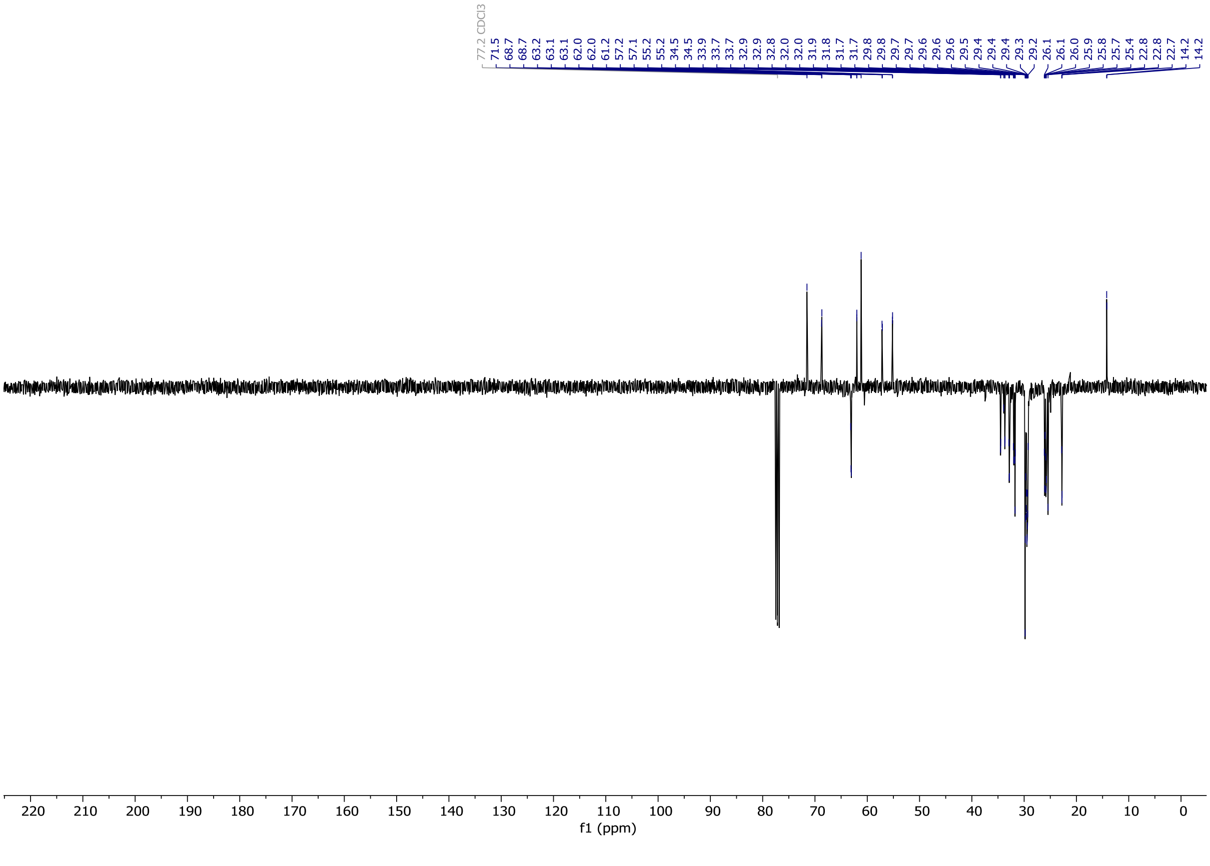

¹³C NMR (101 MHz, $CDCl_3$) of 3-EpAlc.

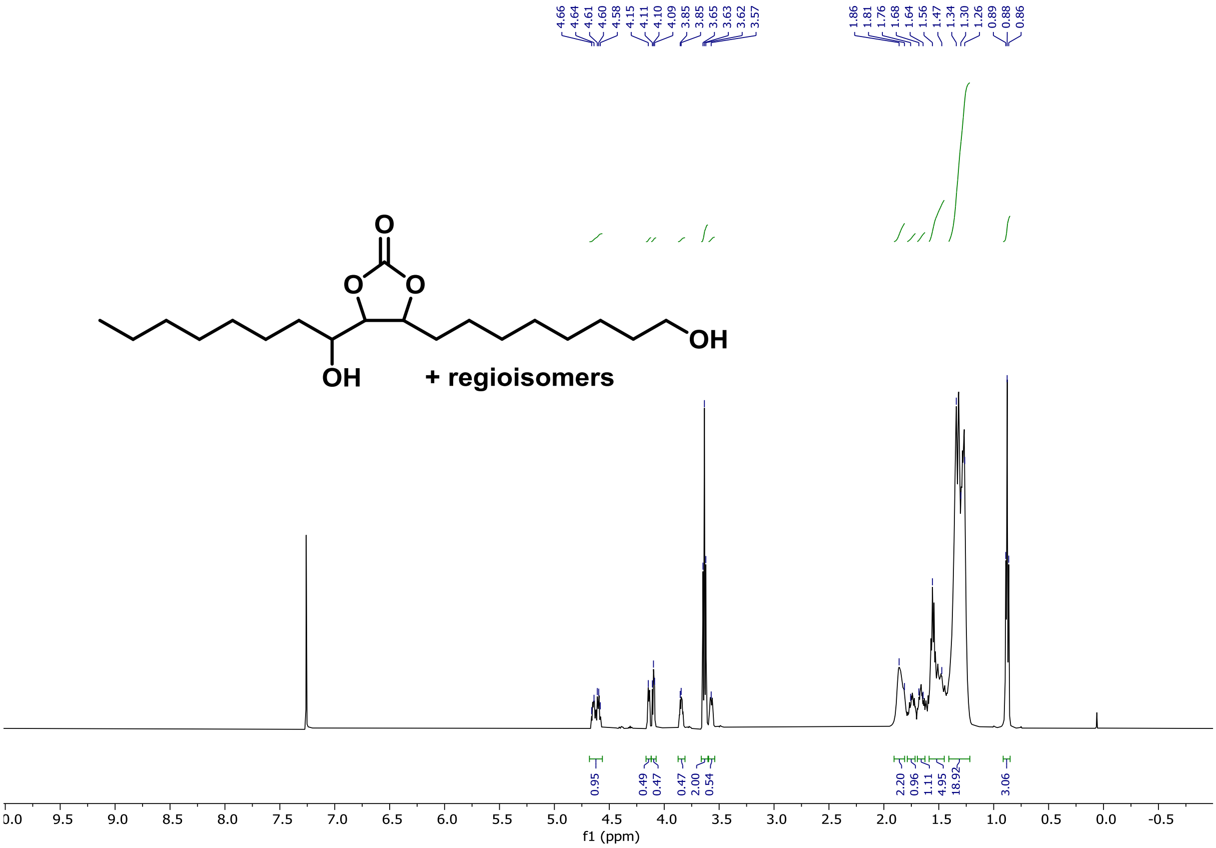

^{1}H NMR (300 MHz, $CDCl_3$) of 3-CC.

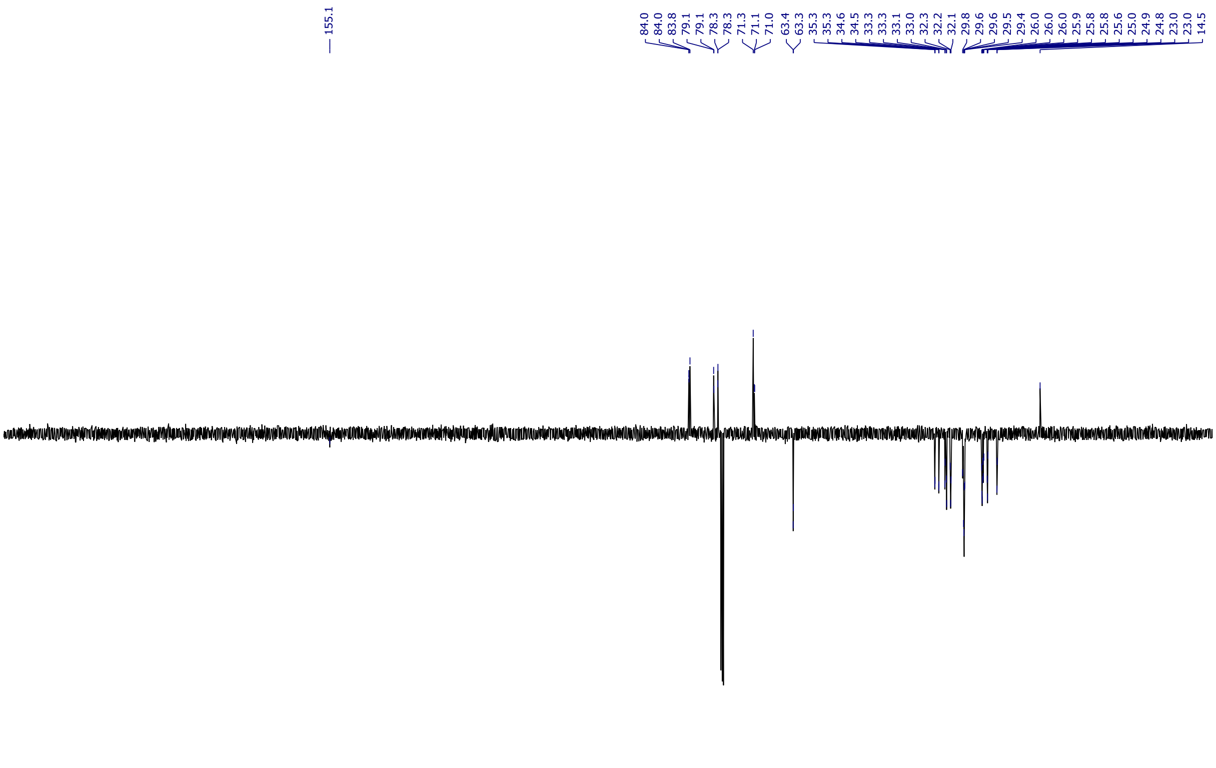

^{13}C NMR (101 MHz, $CDCl_3$) of 3-CC.

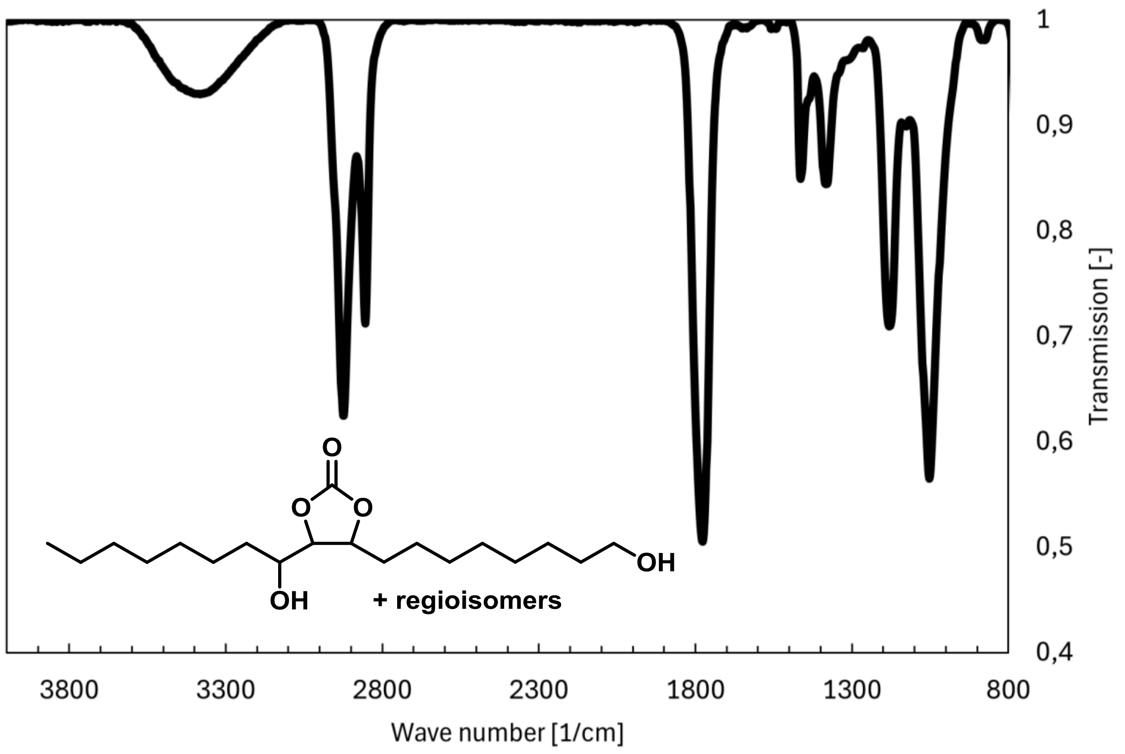

IR-spectrum of 3-CC.

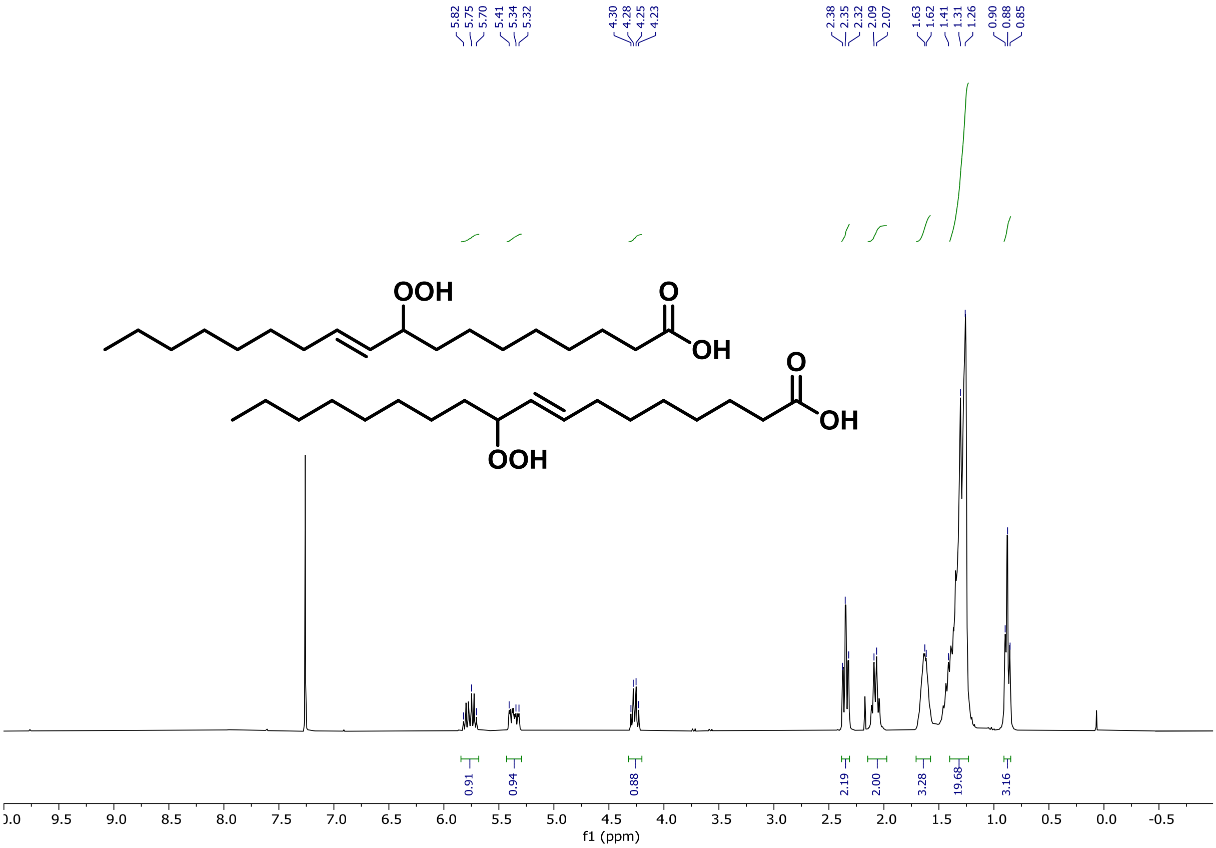

^{1}H NMR (300 MHz, $CDCl_3$) of 4-HYP.

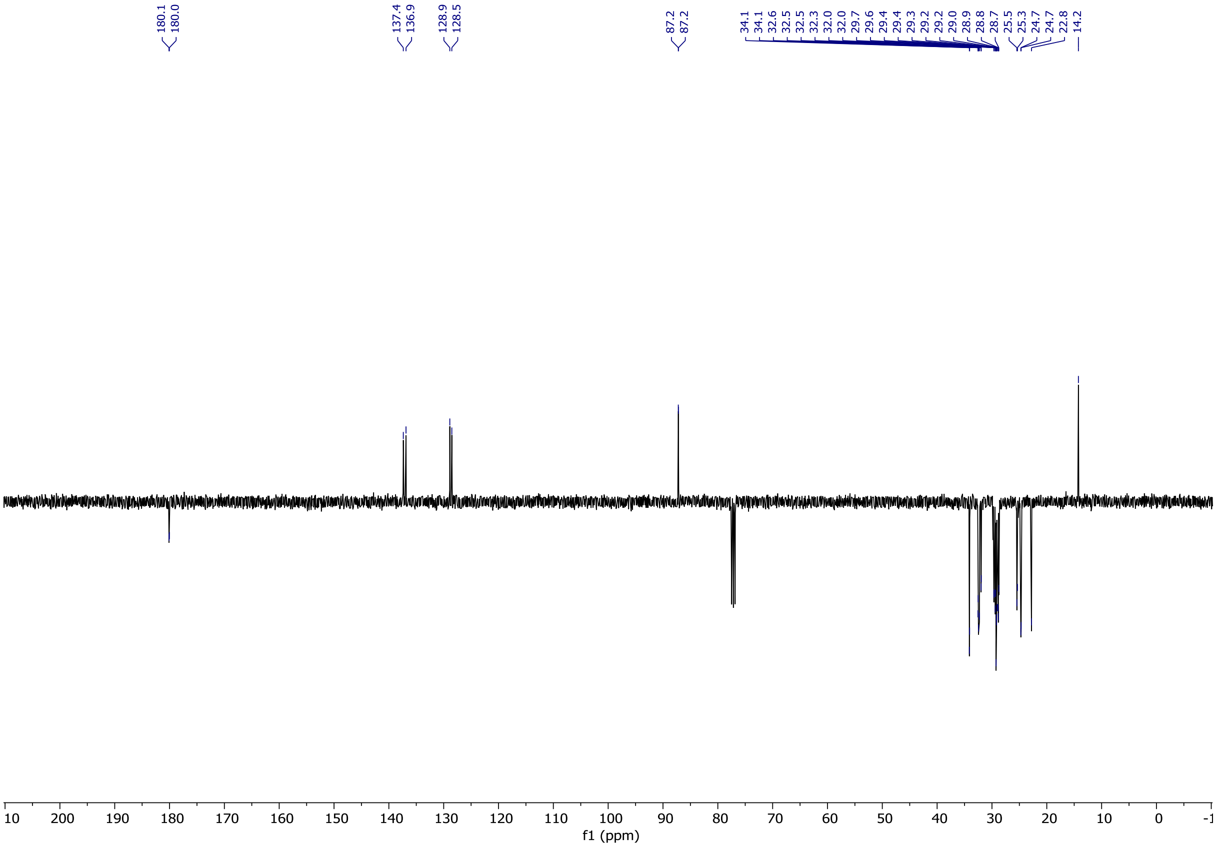

^{13}C NMR (101 MHz, $CDCl_3$) of 4-HYP.

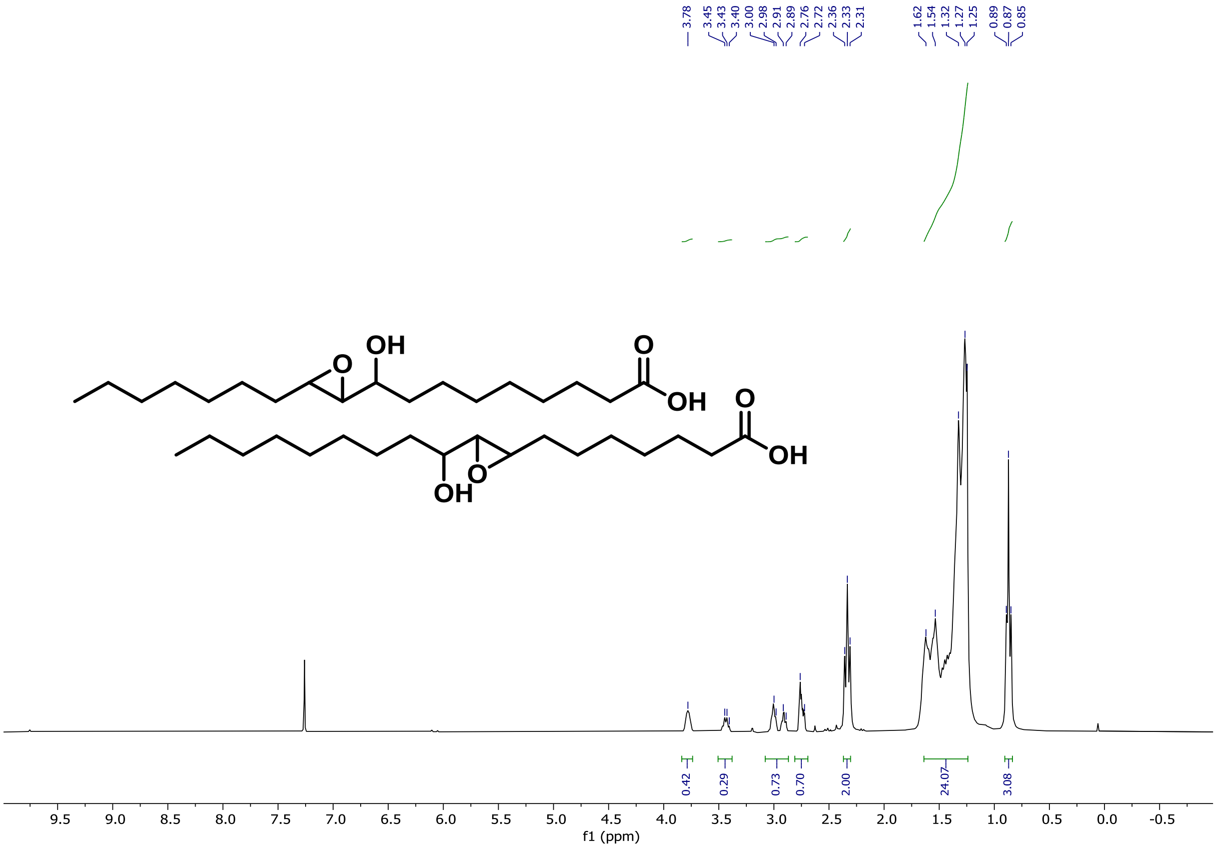

^{1}H NMR (300 MHz, $CDCl_3$) of 4-EpAlc.

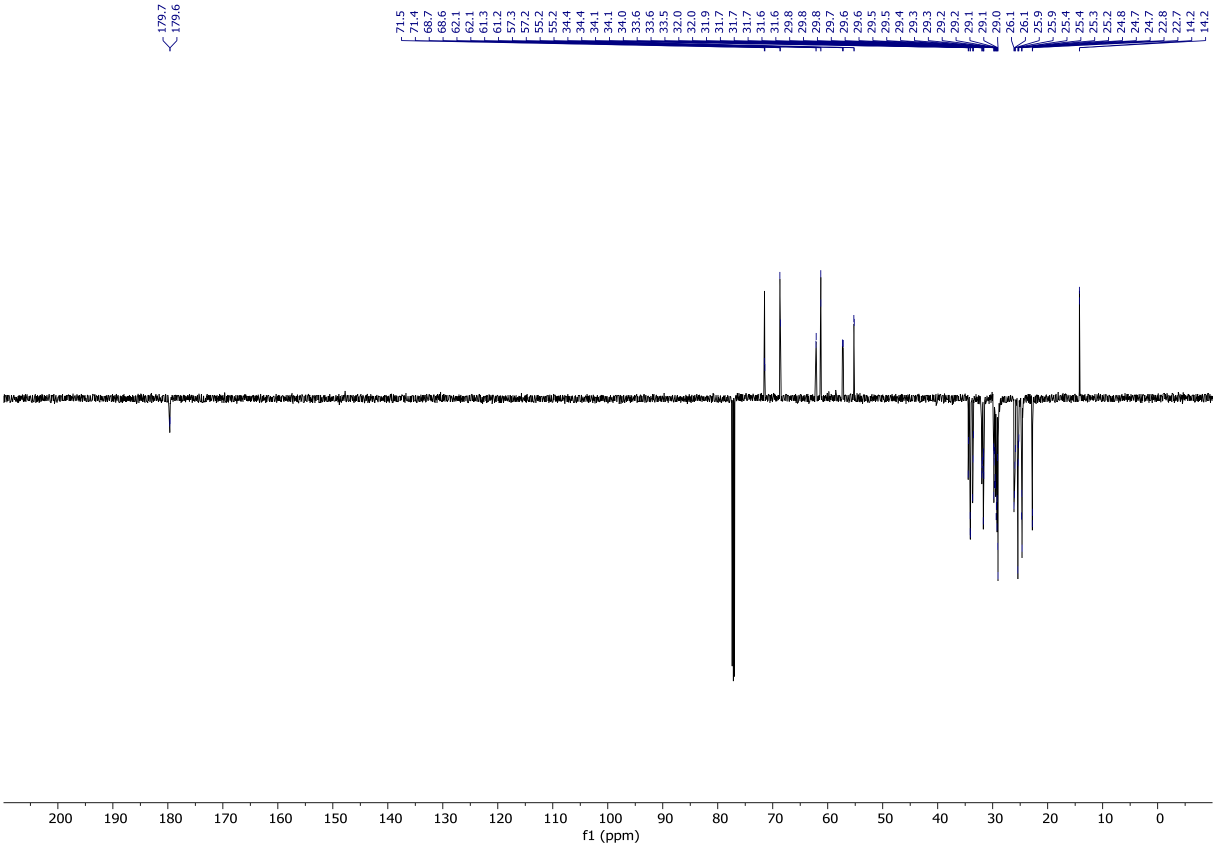

^{13}C NMR (151 MHz, $CDCl_3$) of 4-EpAlc.

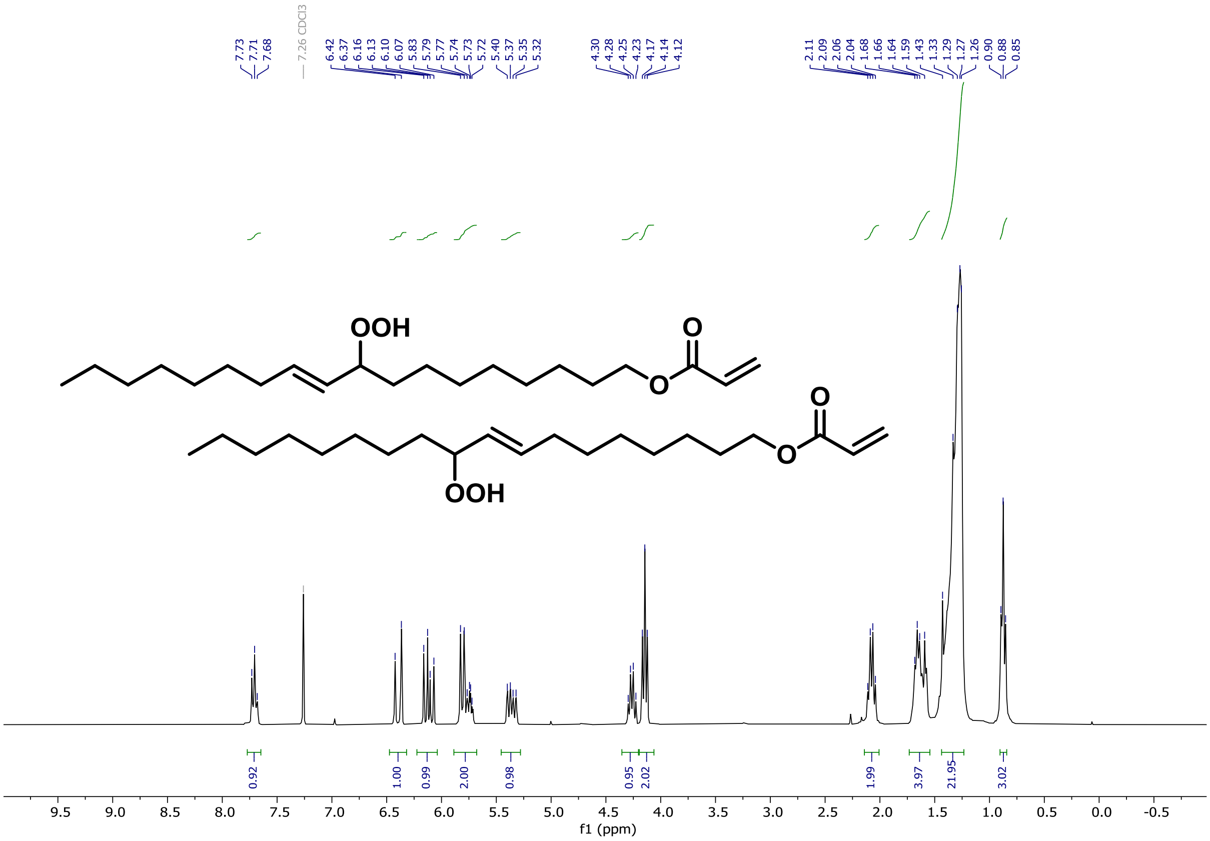

^{1}H NMR (300 MHz, $CDCl_3$) of 6-HYP.

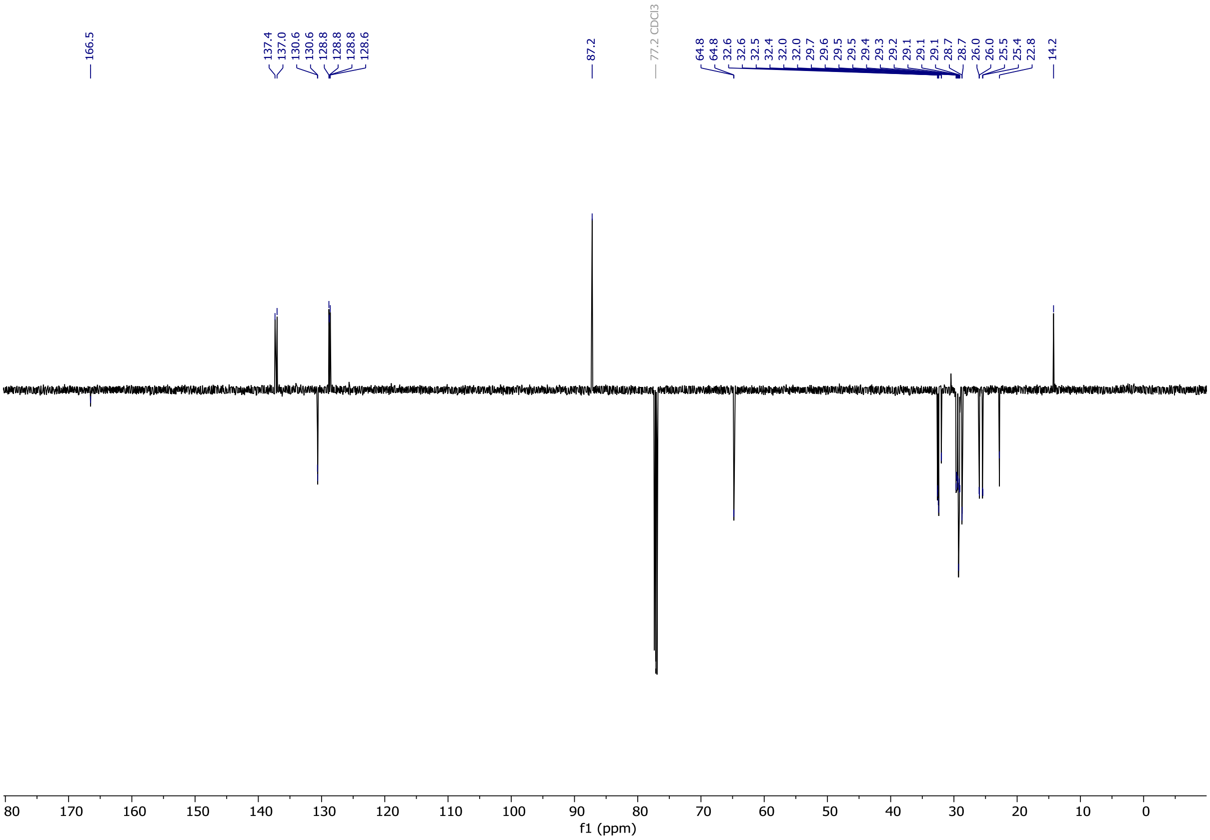

^{13}C NMR (151 MHz, $CDCl_3$) of 6-HYP.

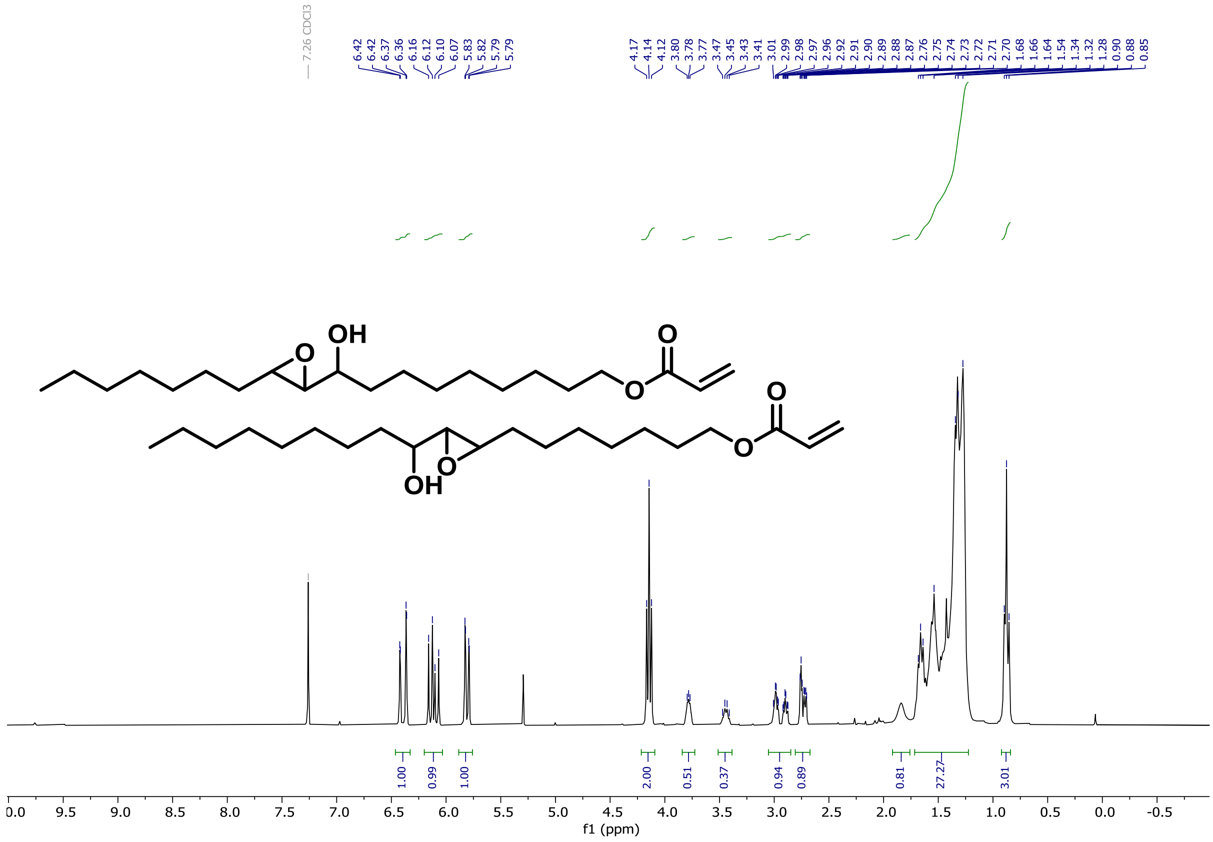

^{1}H NMR (300 MHz, $CDCl_3$) of 6-EpAlc.

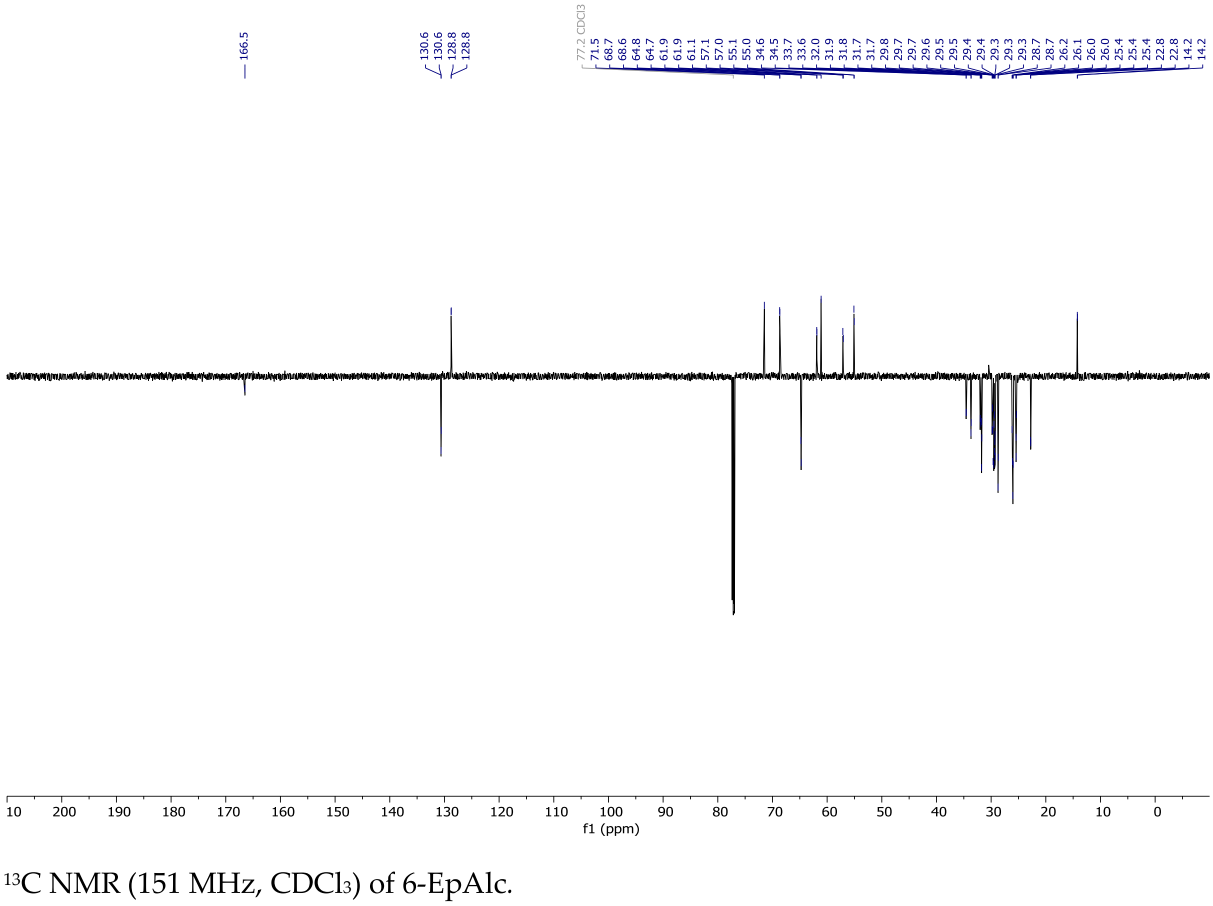

^{13}C NMR (151 MHz, $CDCl_3$) of 6-EpAlc.

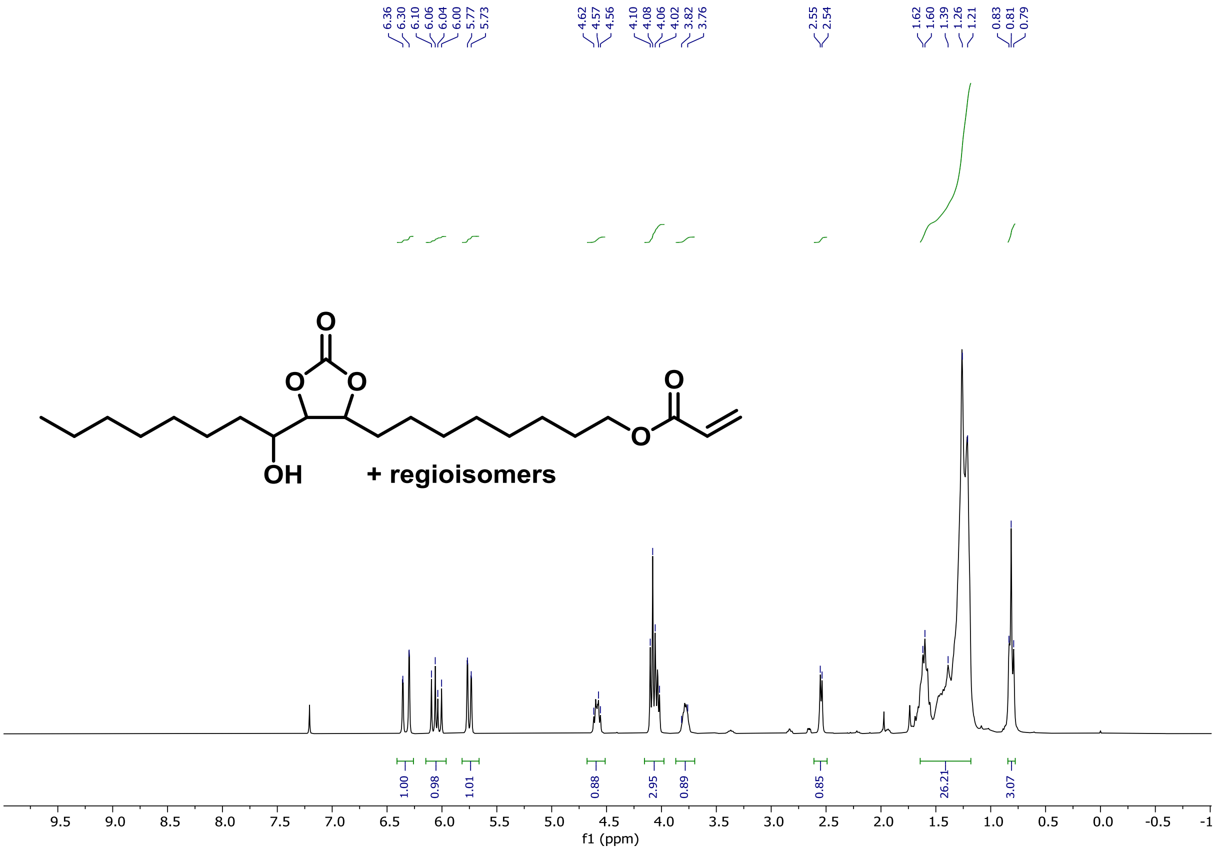

¹H NMR (300 MHz, $CDCl_3$) of 6-CC.

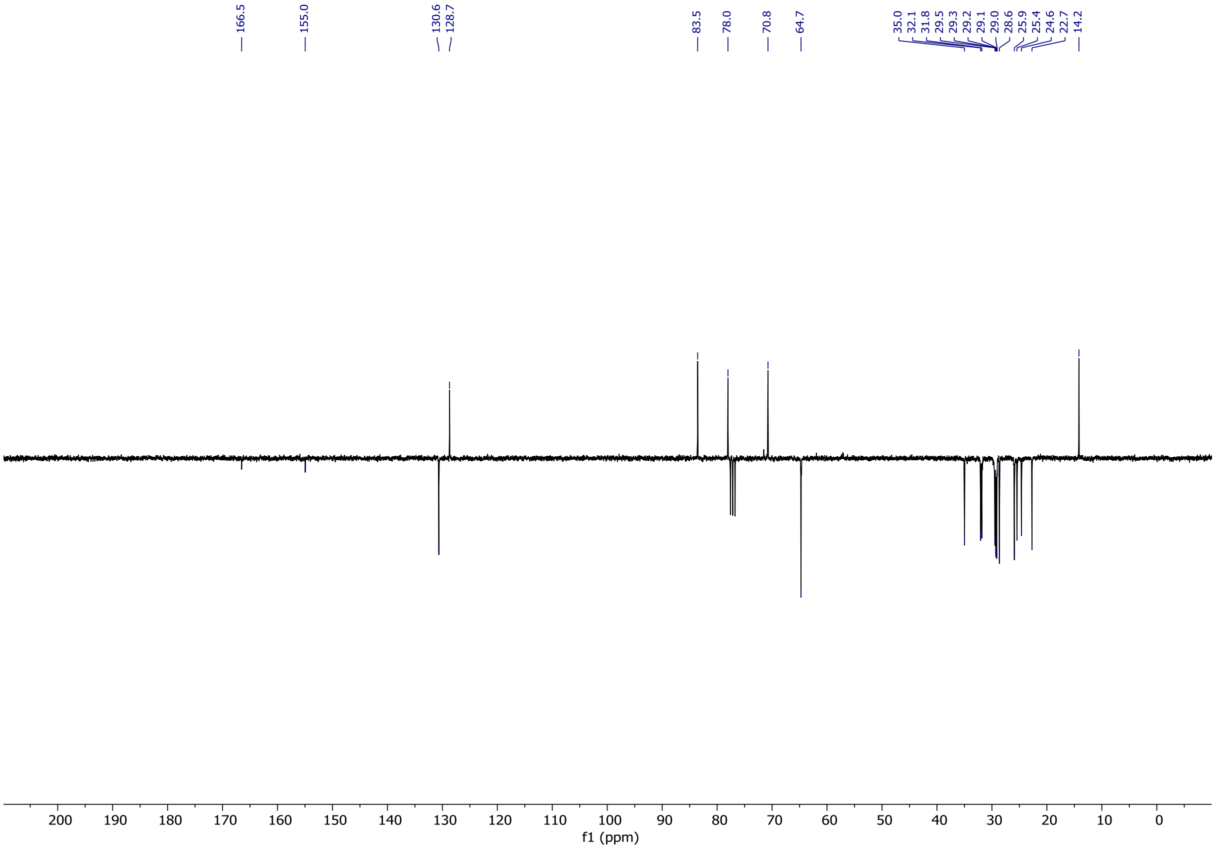

^{13}C NMR (101 MHz, $CDCl_3$) of 6-CC.

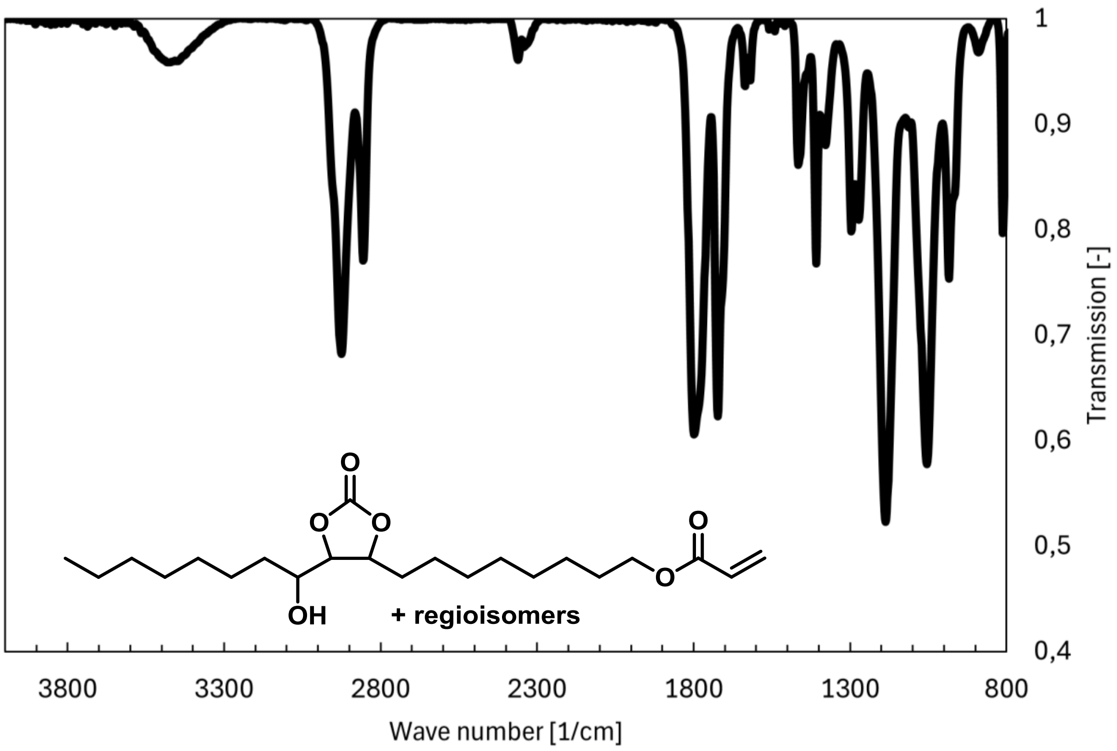

IR-spectrum of 6-CC.

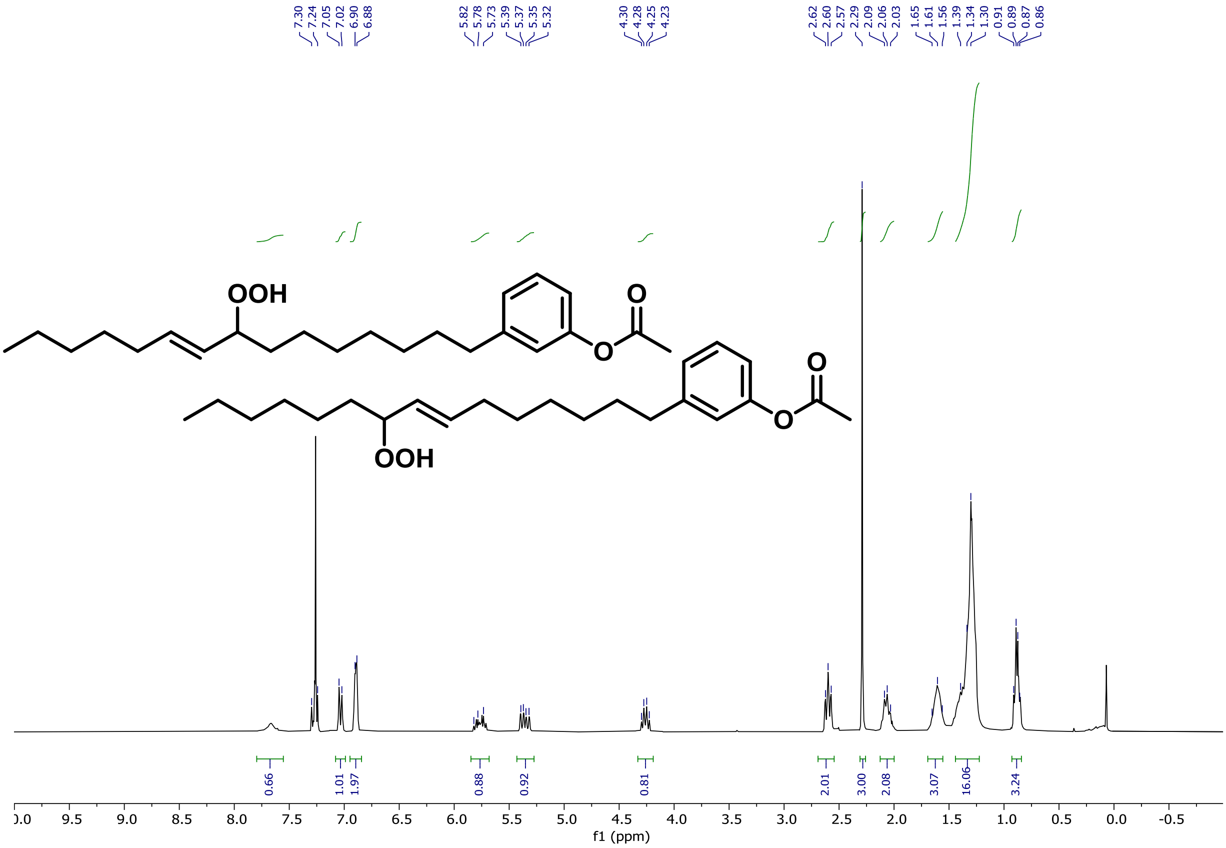

¹H NMR (300 MHz, $CDCl_3$) of 5-HYP.

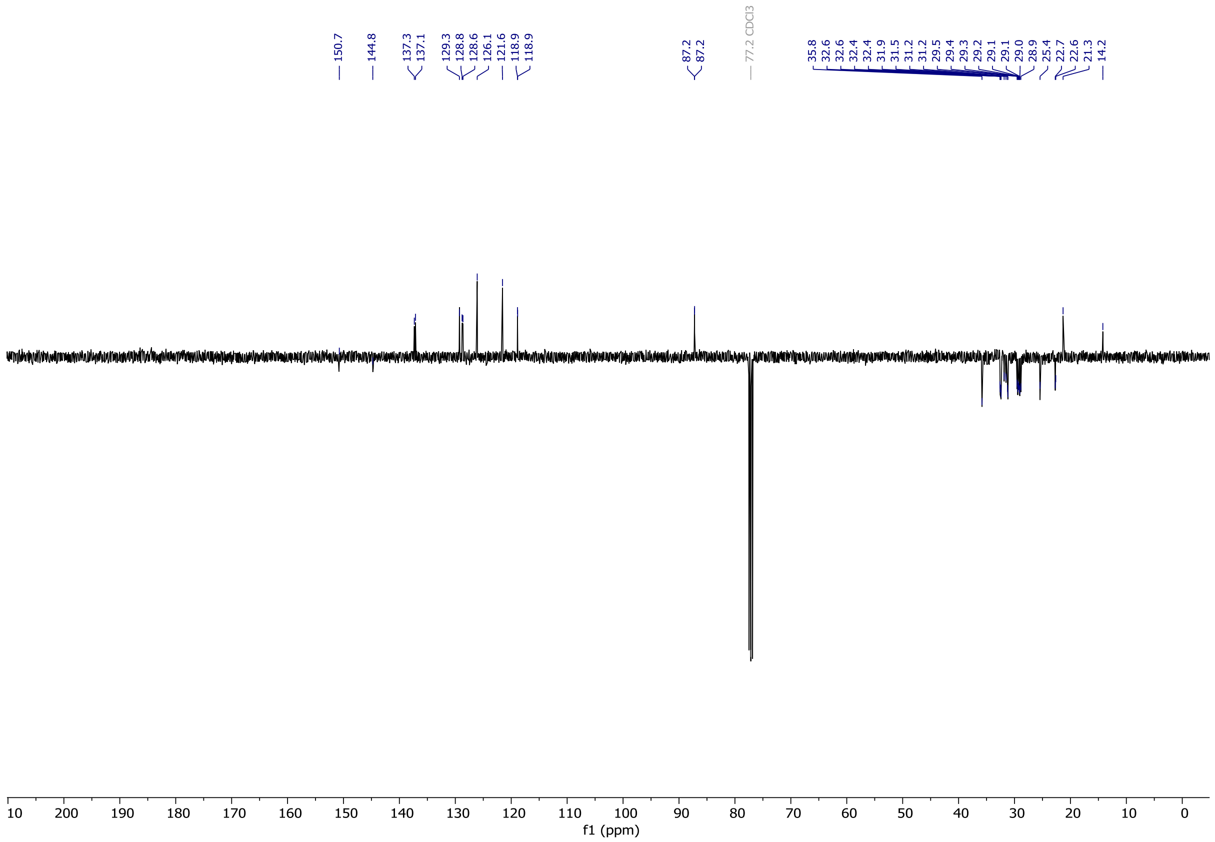

¹³C NMR (101 MHz, $CDCl_3$) of 5-HYP.

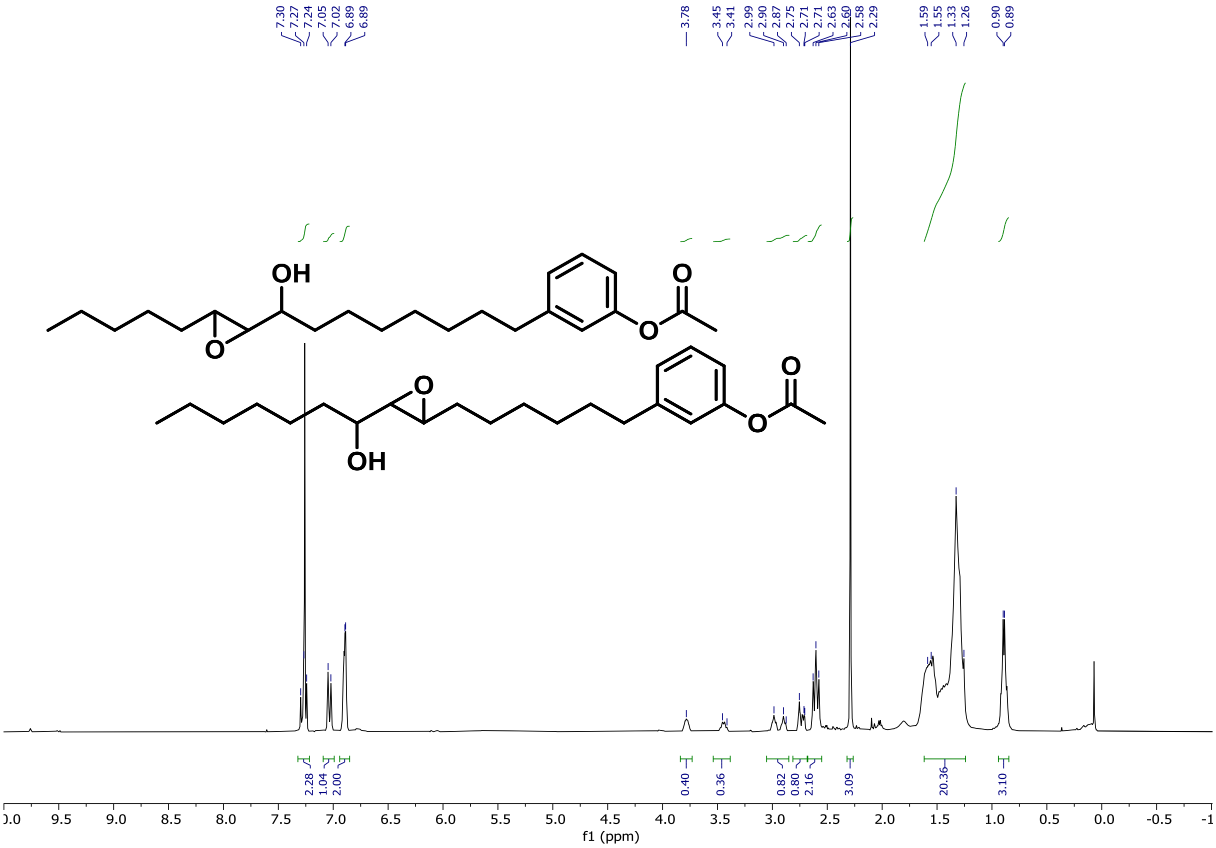

^{1}H NMR (300 MHz, $CDCl_3$) of 5-EpAlc.

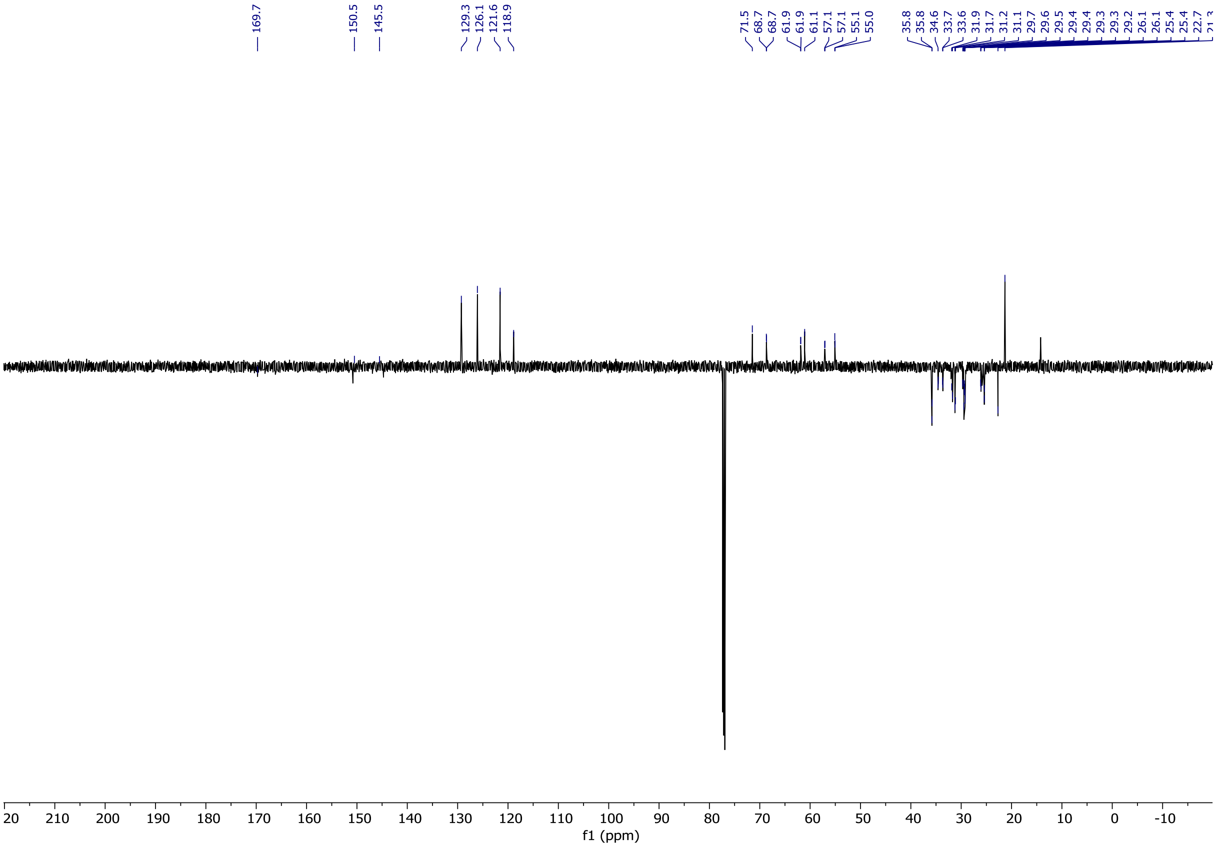

^{13}C NMR (151 MHz, $CDCl_3$) of 5-EpAlc.

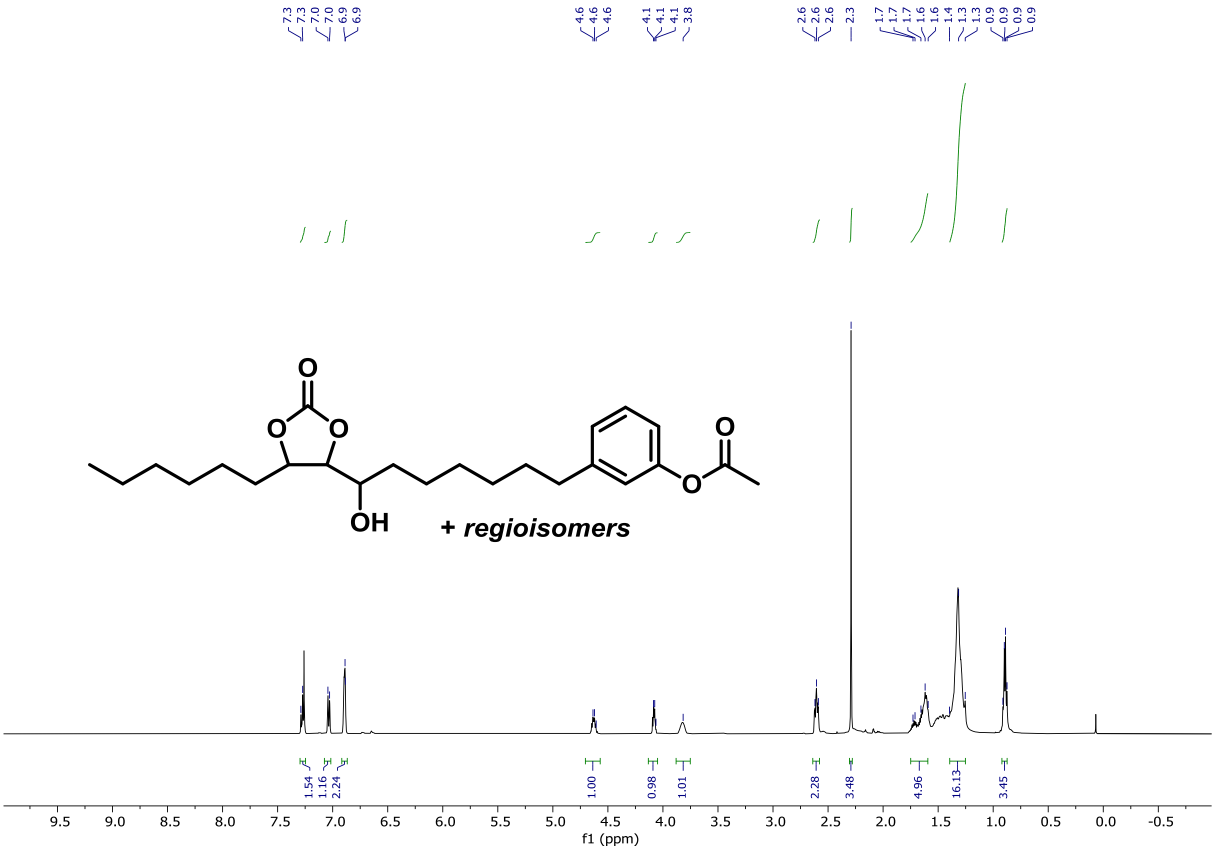

^{1}H NMR (300 MHz, $CDCl_3$) of 5-CC.

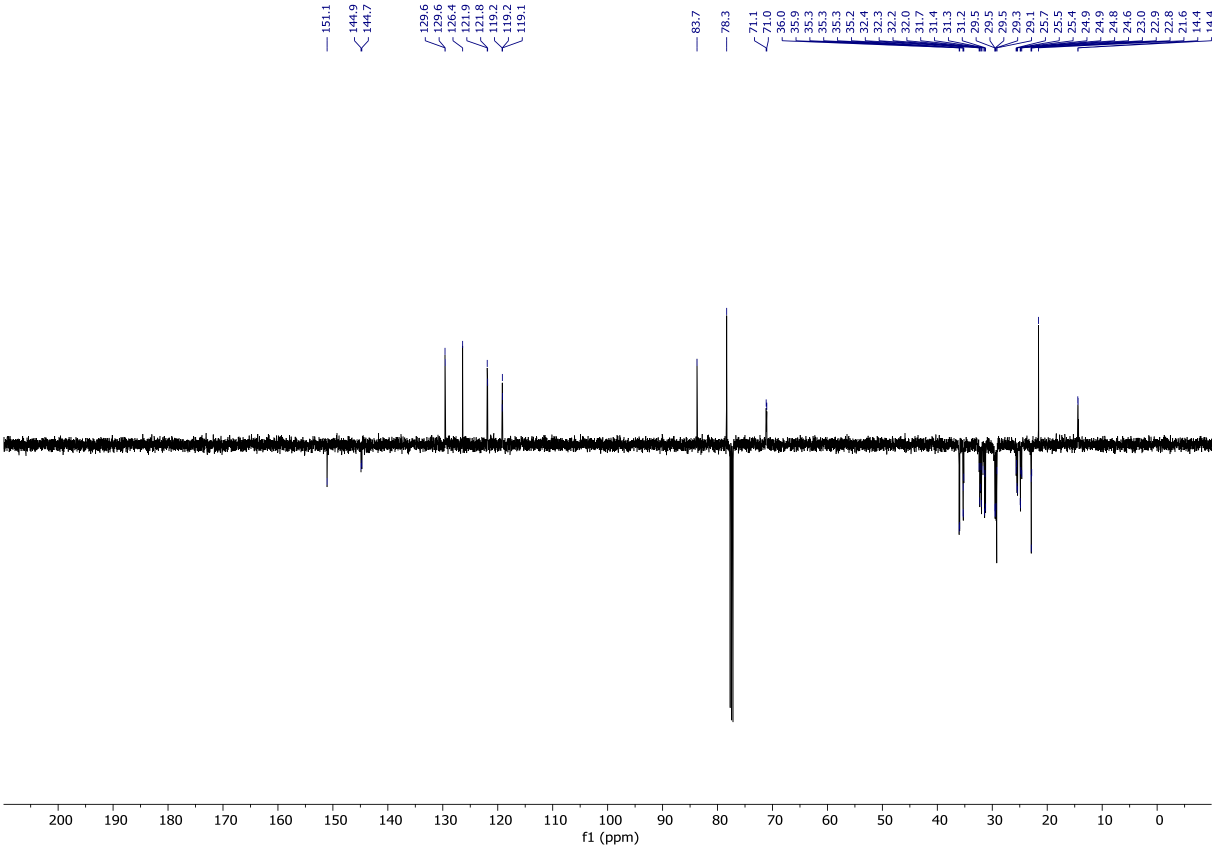

^{13}C NMR (101 MHz, $CDCl_3$) of 5-CC.

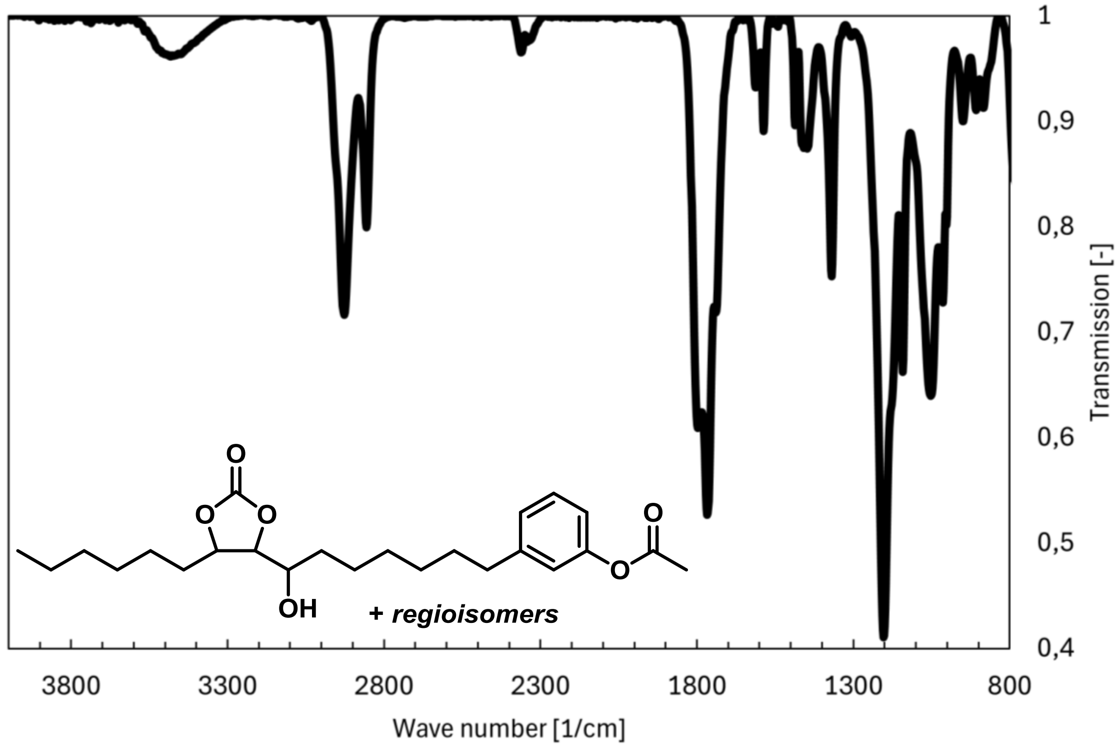

IR-spectrum of 5-CC.

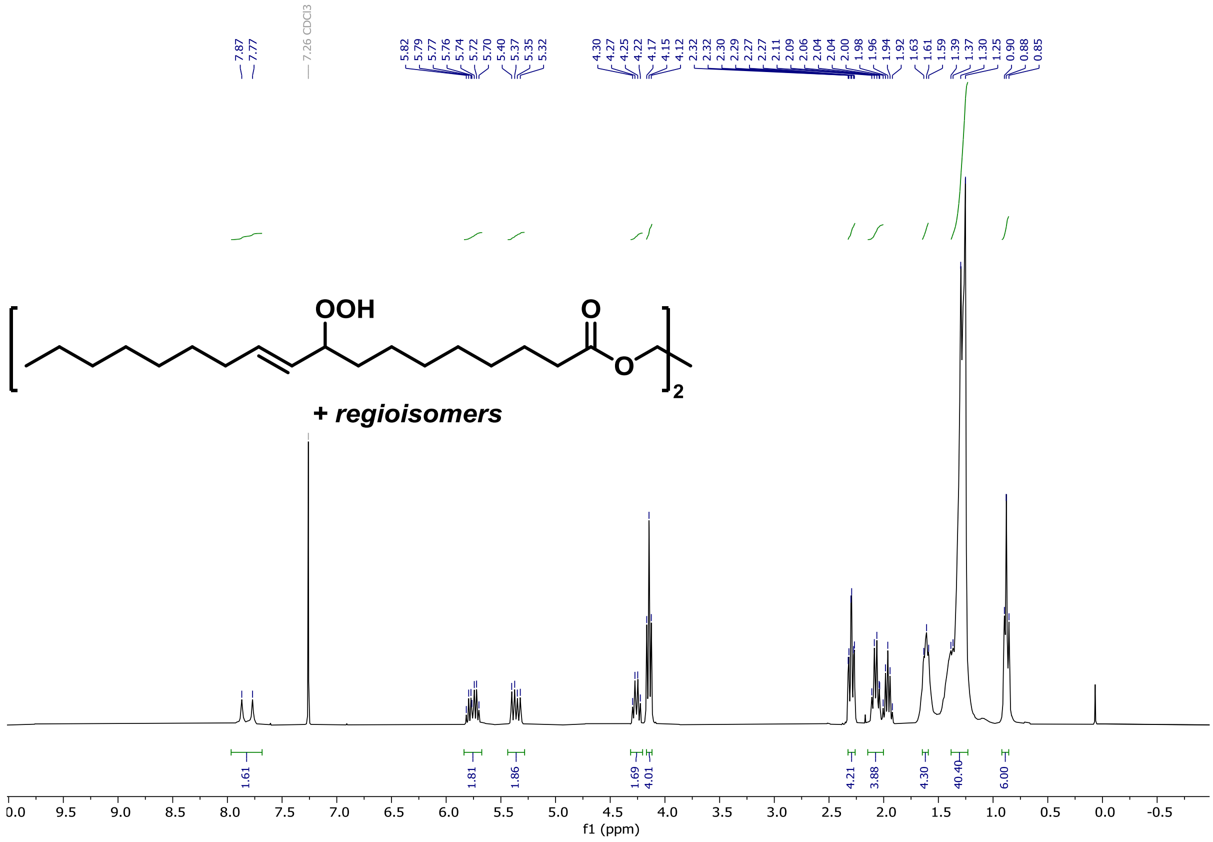

^{1}H NMR (300 MHz, $CDCl_3$) of 7-HYP.

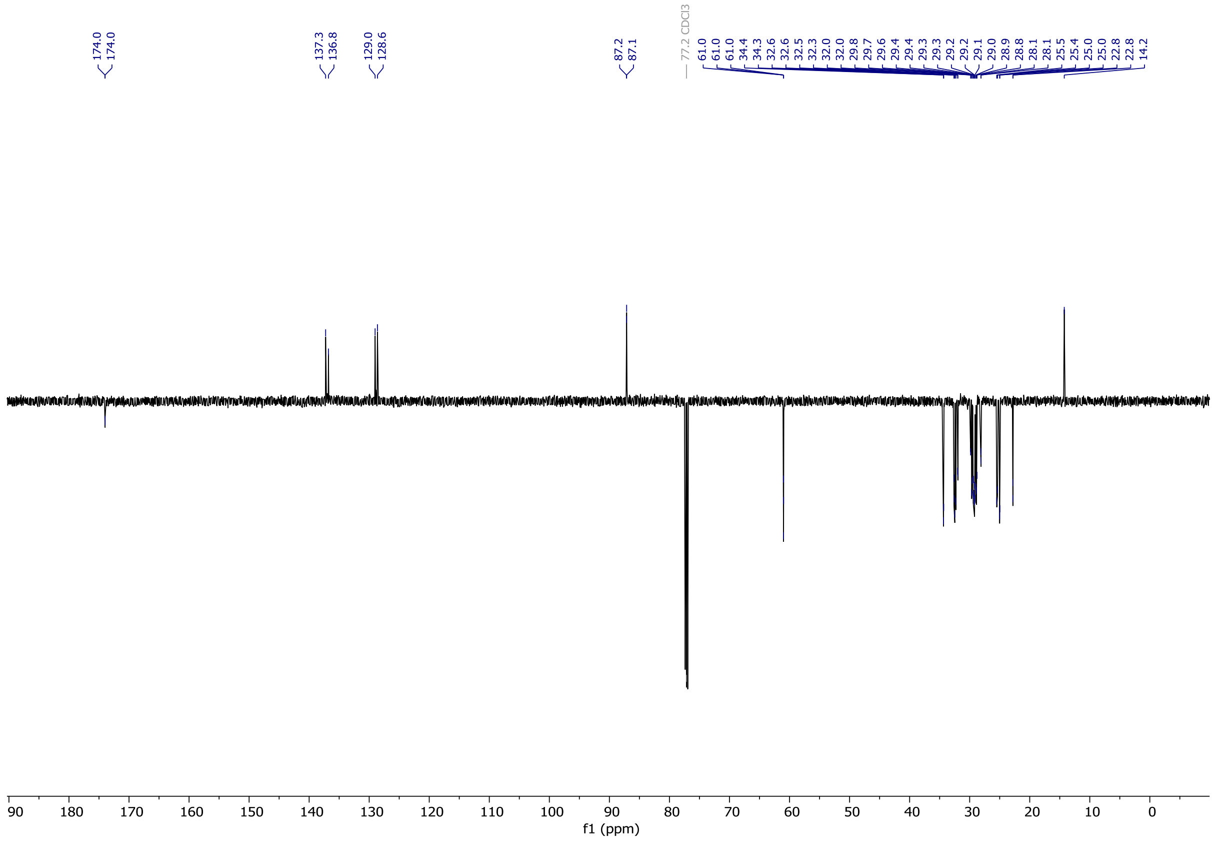

^{13}C NMR (151 MHz, $CDCl_3$) of 7-HYP.

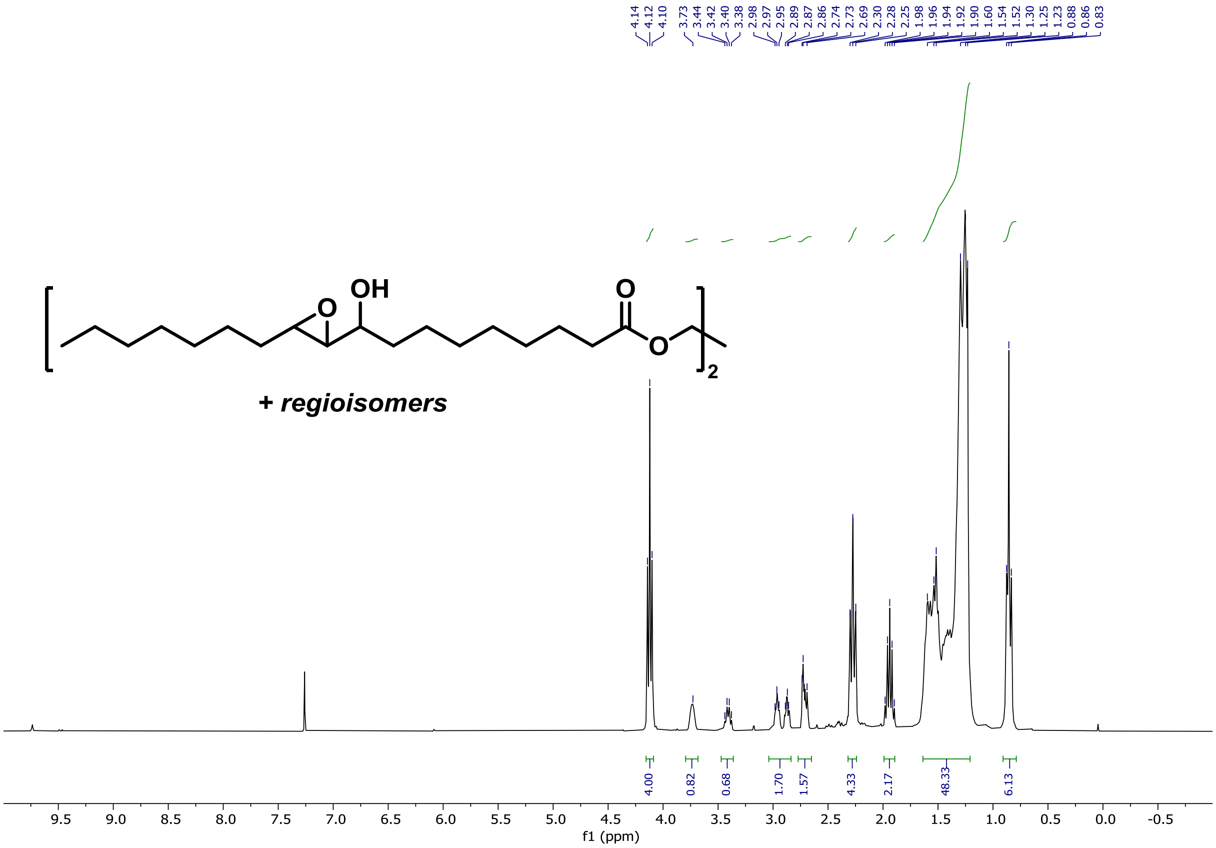

^{1}H NMR (300 MHz, $CDCl_3$) of 7-EpAlc.

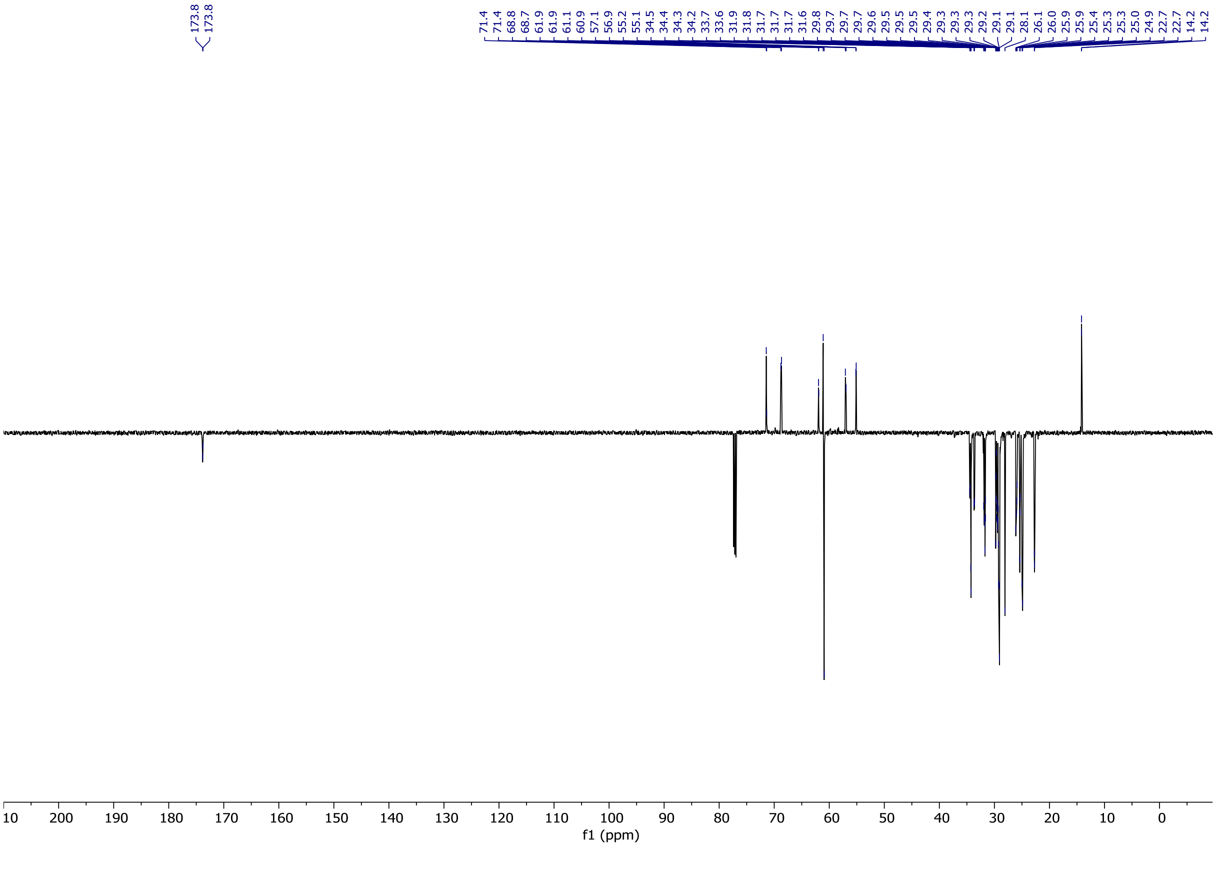

^{13}C NMR (151 MHz, $CDCl_3$) of 7-EpAlc.

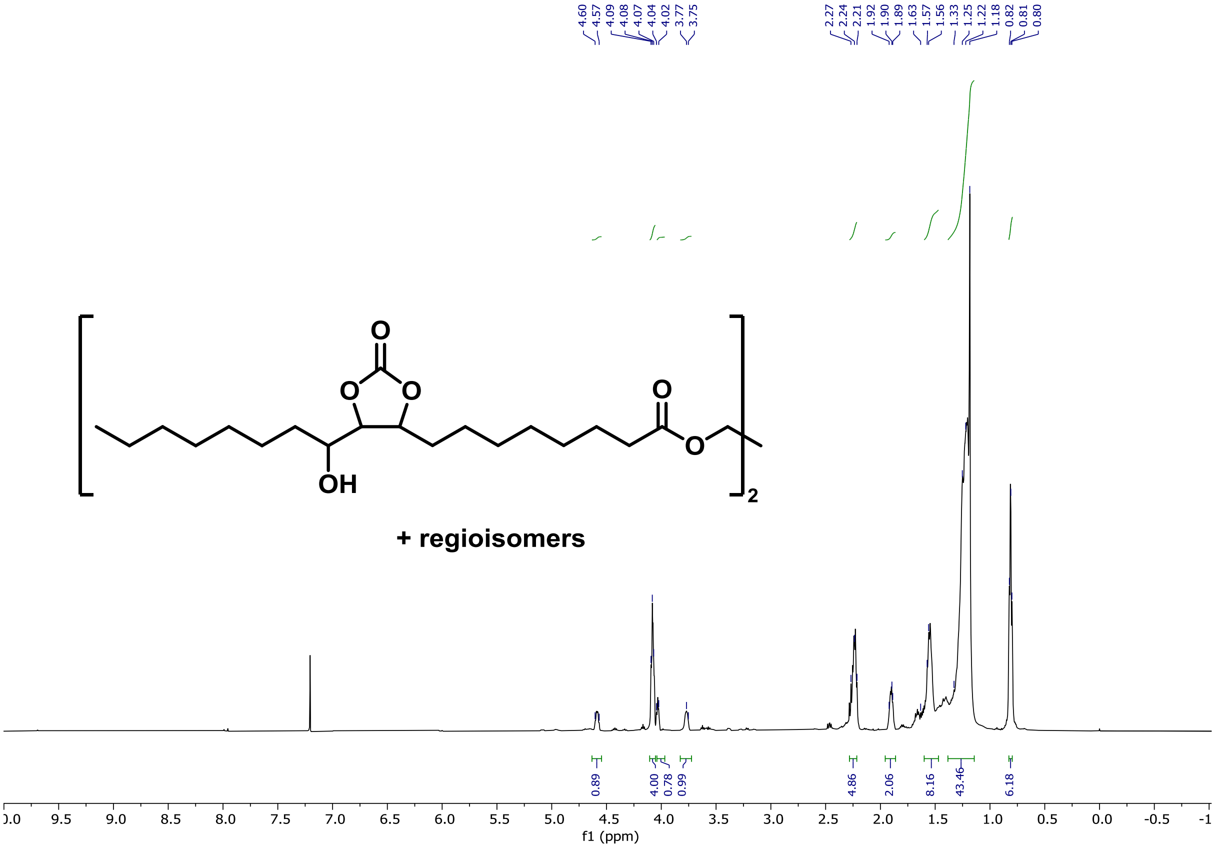

^{1}H NMR (300 MHz, $CDCl_3$) of 7-CC.

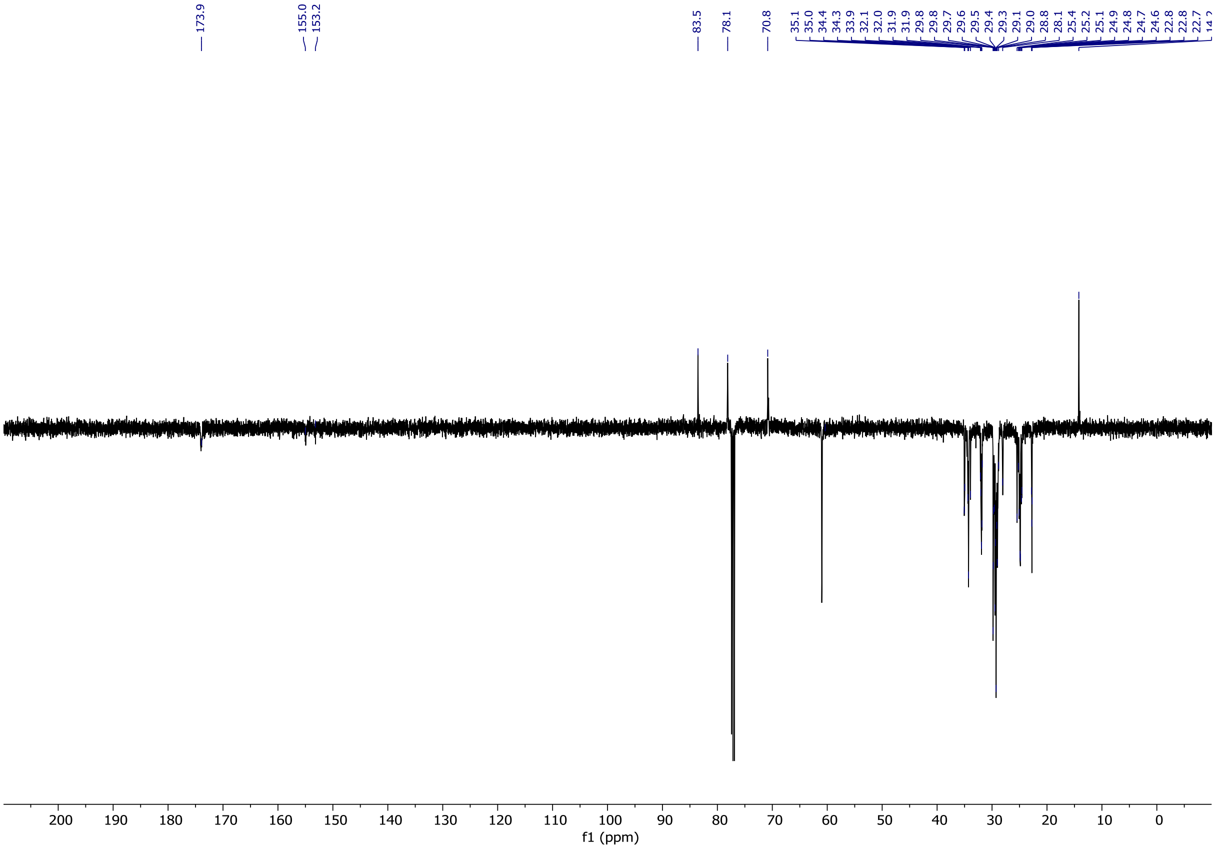

^{13}C NMR (101 MHz, $CDCl_3$) of 7-CC.

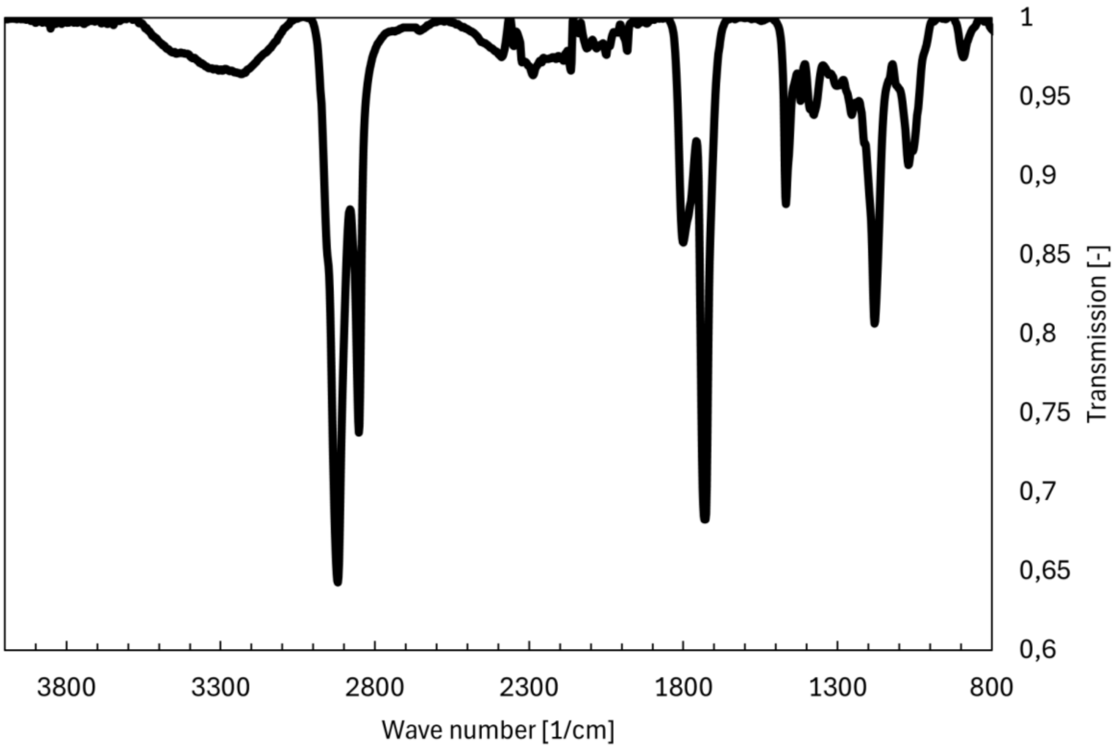

IR spectrum of 7-CC.

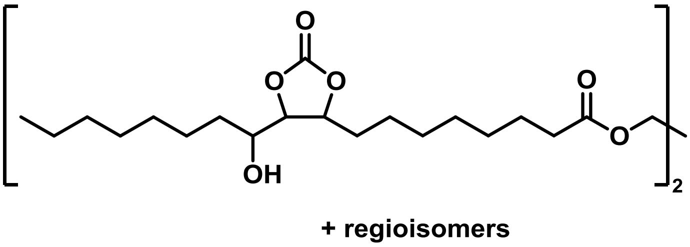

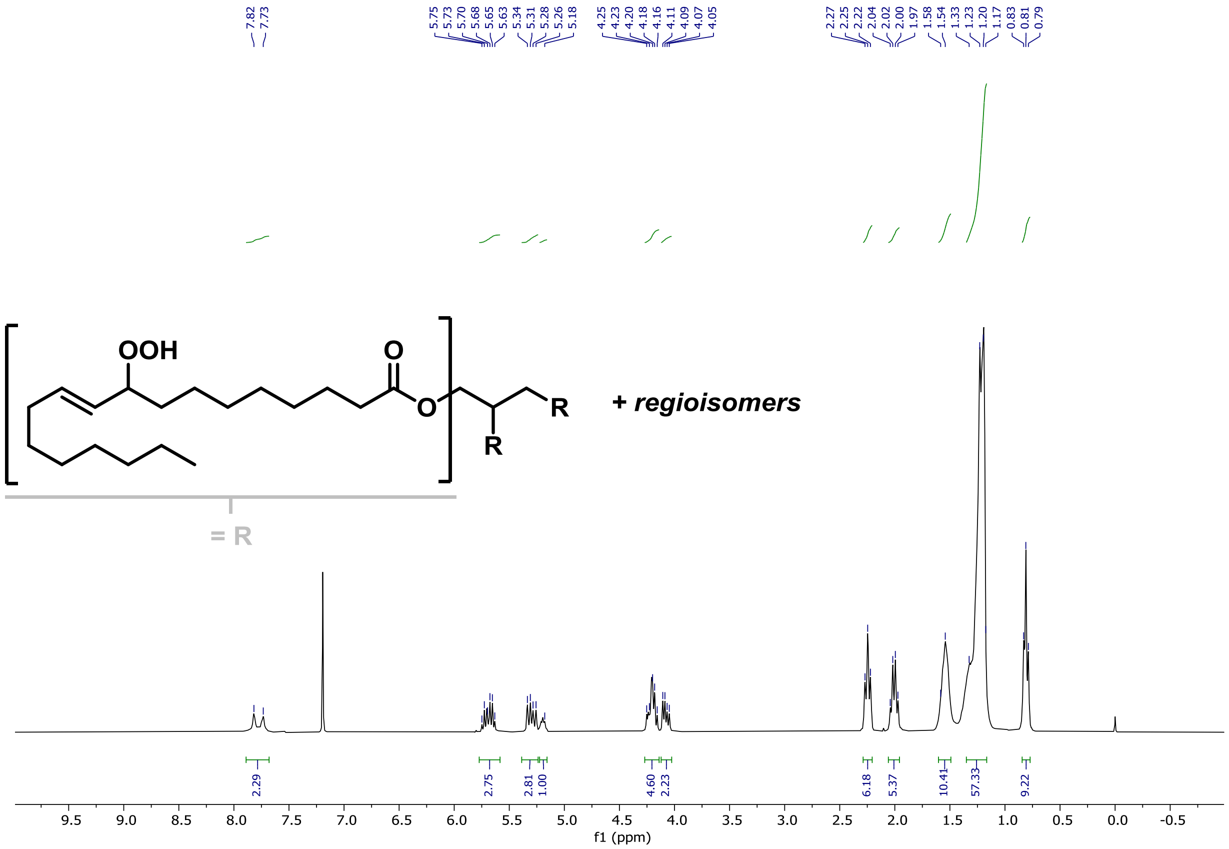

^{1}H NMR (300 MHz, $CDCl_3$) of 8-HYP.

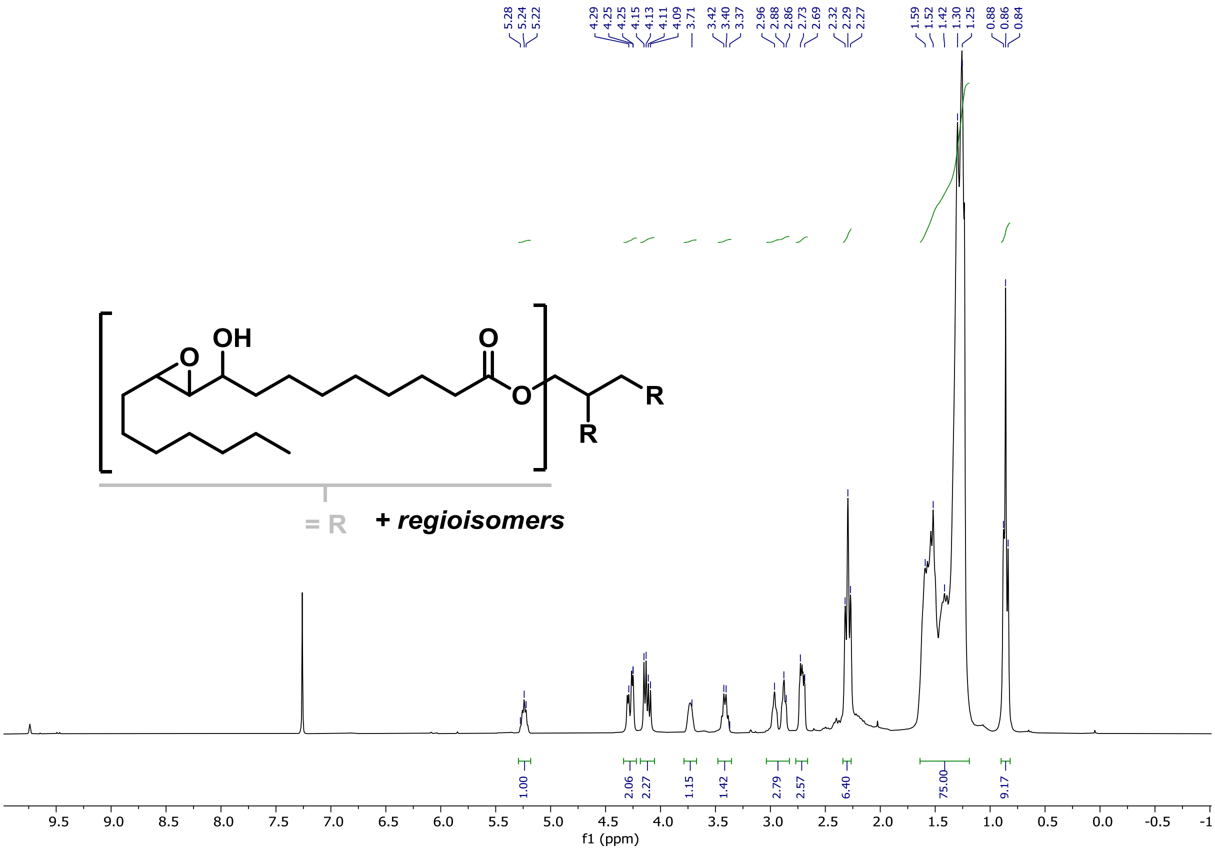

^{1}H NMR (300 MHz, $CDCl_3$) of 8-EpAlc.

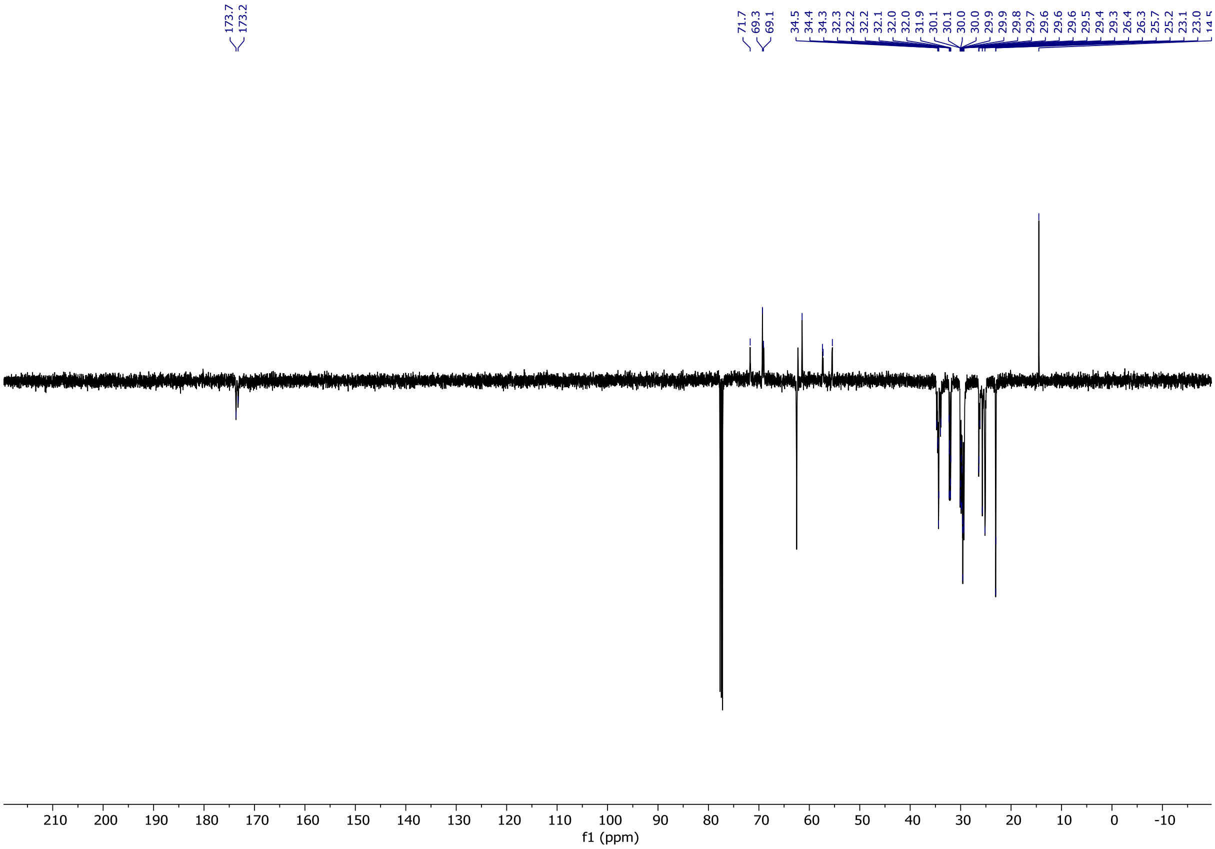

^{13}C NMR (151 MHz, $CDCl_3$) of 8-EpAlc.

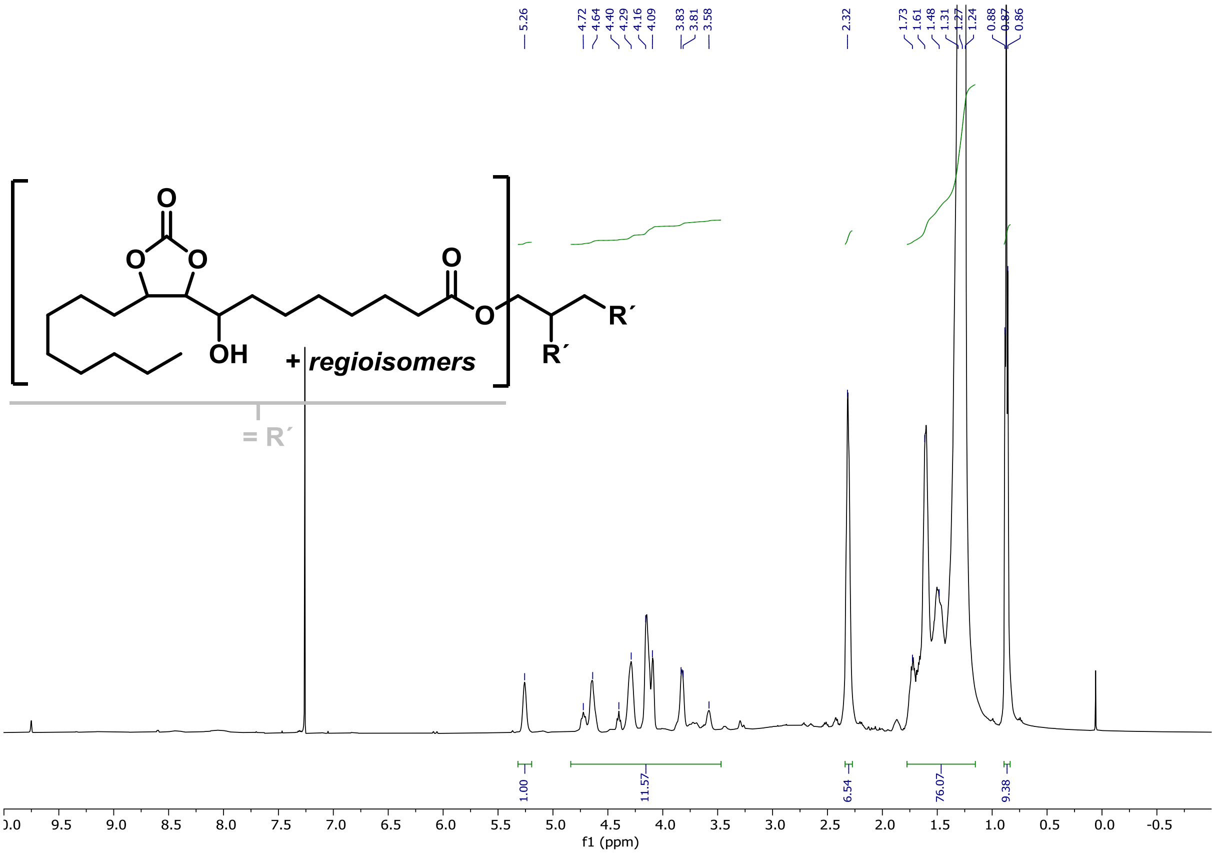

^{1}H NMR (300 MHz, $CDCl_3$) of 8-CC.

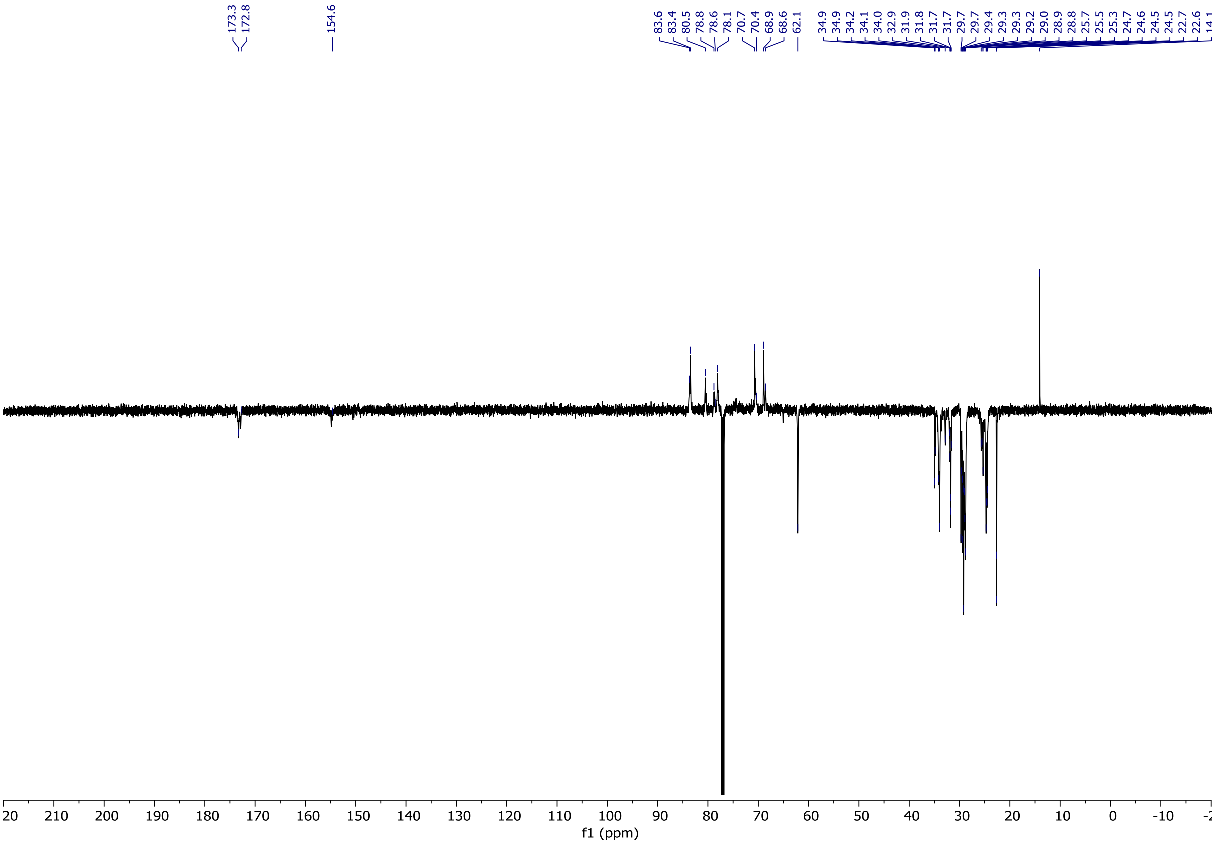

^{13}C NMR (151 MHz, $CDCl_3$) of 8-CC.

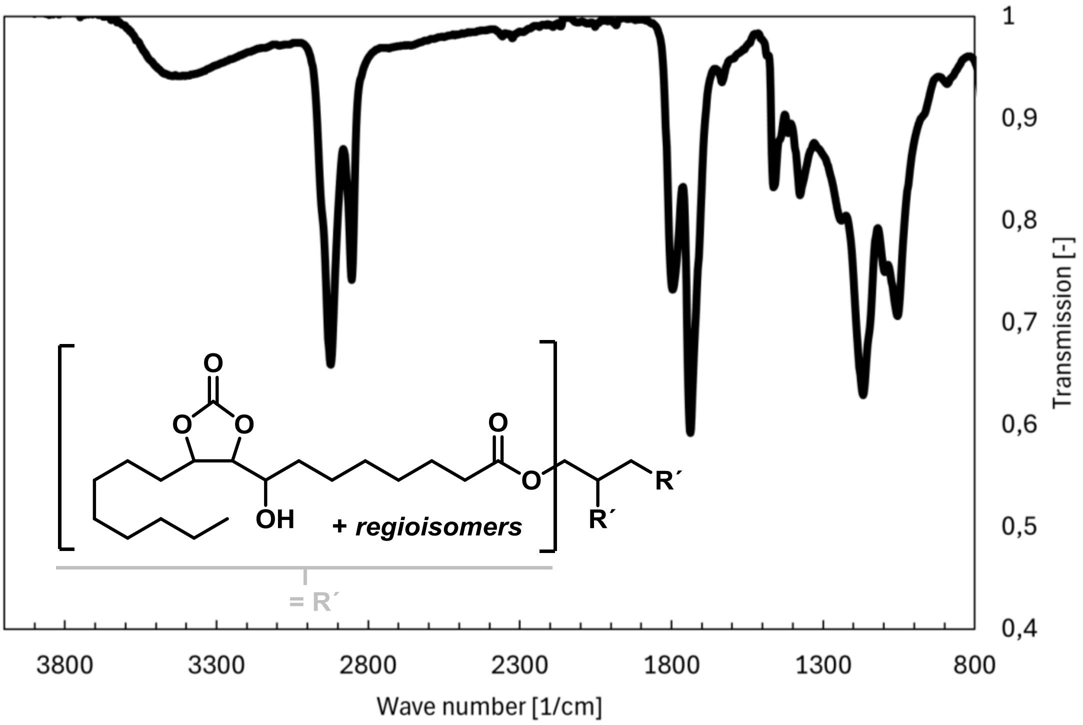

IR-spectrum of 8-CC.
